# Structural insights into a shared mechanism of human STING activation by a potent agonist and an autoimmune disease-associated mutation

**DOI:** 10.1038/s41421-022-00481-4

**Published:** 2022-12-13

**Authors:** Zuoquan Xie, Zhen Wang, Fengying Fan, Jinpei Zhou, Zhaoxue Hu, Qingxia Wang, Xiyuan Wang, Qingzhong Zeng, Yan Zhang, Jiaxuan Qiu, Xiaoqian Zhou, Hui Xu, Hudagula Bai, Zhengsheng Zhan, Jian Ding, Huibin Zhang, Wenhu Duan, Xuekui Yu, Meiyu Geng

**Affiliations:** 1 State Key Laboratory of Drug Research, Shanghai Institute of Materia Medica, Chinese Academy of Sciences, Shanghai, China; 2 Cryo-Electron Microscopy Research Center & The CAS Key Laboratory of Receptor Research, Shanghai Institute of Materia Medica, Chinese Academy of Sciences, Shanghai, China; 3 Department of Medicinal Chemistry & Center of Drug Discovery, China Pharmaceutical University, Nanjing, China; 4 School of Life Science and Technology, ShanghaiTech University, Shanghai, China; 5 University of Chinese Academy of Sciences, Beijing, China; 6 Small-Molecule Drug Research Center, Shanghai Institute of Materia Medica, Chinese Academy of Sciences, Shanghai, China

**Keywords:** Cryoelectron microscopy, Immunosurveillance

## Abstract

Stimulator of interferon gene (STING) is increasingly exploited for the potential in cancer immunotherapy, yet its mechanism of activation remains not fully understood. Herein, we designed a novel STING agonist, designated as HB3089 that exhibits robust and durable anti-tumor activity in tumor models across various cancer types. Cryo-EM analysis reveals that HB3089-bound human STING has structural changes similar to that of the STING mutant V147L, a constitutively activated mutant identified in patients with STING-associated vasculopathy with onset in infancy (SAVI). Both structures highlight the conformational changes of the transmembrane domain (TMD), but without the 180°-rotation of the ligand binding domain (LBD) previously shown to be required for STING activation. Further structure-based functional analysis confirmed a new STING activation mode shared by the agonist and the SAVI-related mutation, in which the connector linking the LBD and the TMD senses the activation signal and controls the conformational changes of the LBD and the TMD for STING activation. Together, our findings lead to a new working model for STING activation and open a new avenue for the rationale design of STING-targeted therapies either for cancer or autoimmune disorders.

## Introduction

Stimulator of interferon gene (STING, also known as TMEM173, MITA, ERIS and MPYS) is an endoplasmic reticulum (ER) membrane protein that serves as a sensor of cyclic dinucleotides (CDNs) derived from infected bacteria or produced by the cyclic GMP-AMP synthase (cGAS)^1–10^. Upon binding with CDNs, STING translocates from the ER to the Golgi apparatus, wherein STING is polymerized to recruit and activate downstream TANK-binding kinase 1 (TBK1). The activation of TBK1 in turn activates the transcription factor IRF3 to induce the production of type 1 interferons (IFNs), leading to the spontaneous immune responses^11–13^.

The activity of STING is tightly regulated^14,15^ and its dysfunction could result in autoinflammatory disorders and autoimmune syndromes, such as multiple sclerosis, lupus erythematosus, and Aicardi syndrome^16–22^. In fact, several gain-of-function mutants of STING have been identified in patients with STING-associated vasculopathy with onset in infancy (SAVI) or those with lupus-like syndromes. Structural insights into these mutants that are constitutively activated without CDNs stimulation^20–22^, may provide important information to understand the mechanism of STING activation.

Recently, substantial progress has been made in understanding the mechanism of STING activation. Both structural and biochemical studies revealed that CDN binding induced closed conformational changes of the ligand binding domain (LBD) of STING^23–29^. Non-nucleotide compounds such as diABZI could effectively activate STING signaling, whereas the LBD of STING still has an open conformation^29,30^. However, it remains elusive how the conformational change of LBD leads to STING activation.

In the present study, we designed a new diABZI-based STING agonist HB3089 that triggers substantial anti-tumor immunity and exhibits robust anti-tumor activity. We resolved the cryo-electron microscopy (cryo-EM) structure of full-length human STING bound with HB3089, which allows us to reveal the conformational changes of STING transmembrane domain (TMD) during agonist-induced STING activation. Importantly, this finding was corroborated in the structural analysis of a SAVI-related STING V147L mutant. Our findings lead to a new working model for STING activation and demonstrate HB3089 as a promising drug candidate currently undergoing preclinical development.

## Results

### A new STING agonist HB3089 exhibits robust anti-tumor activity

**Fig. 1:**
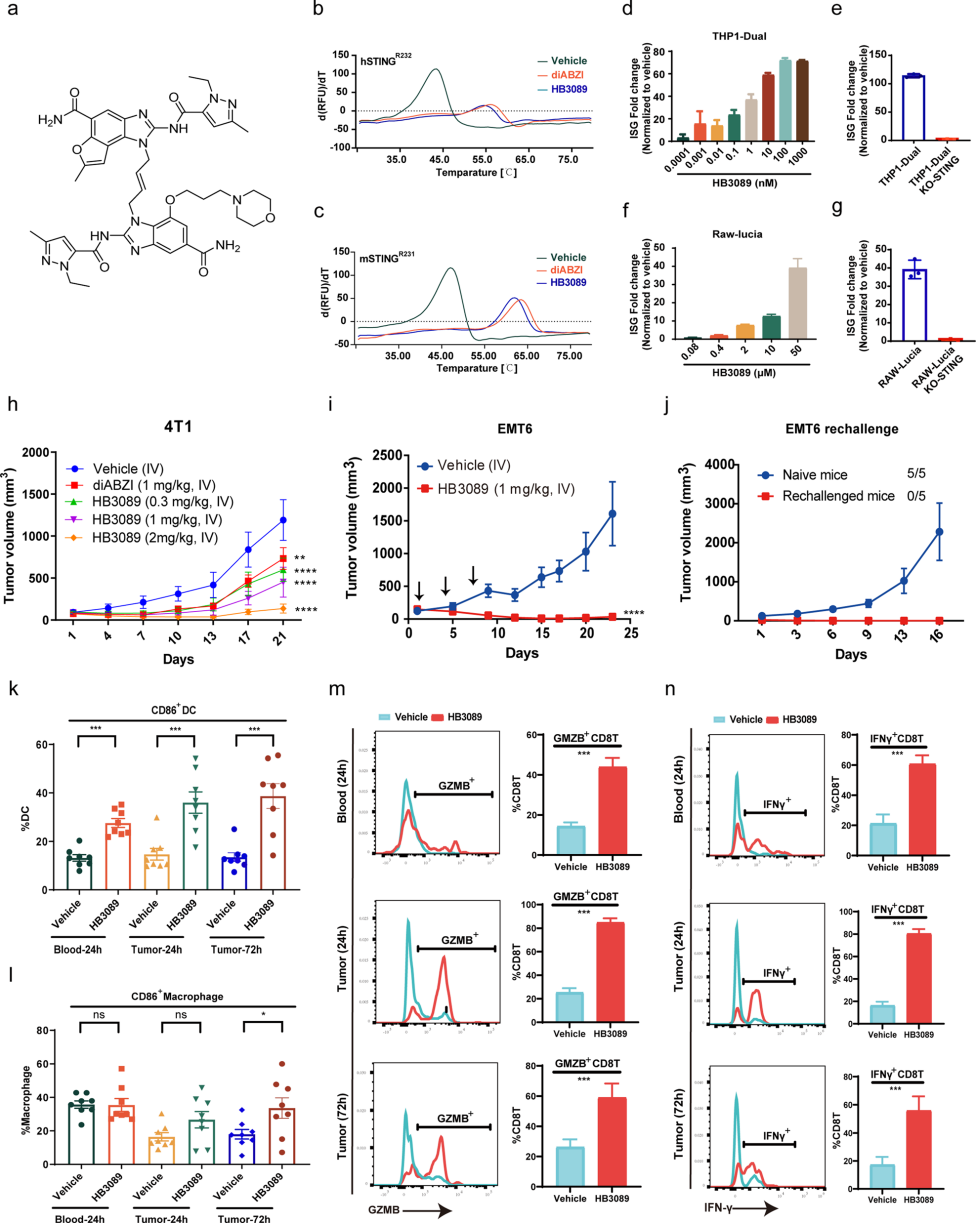
Discovery of a novel potent STING agonist HB3089. **a** Chemical structure of HB3089. **b, c** The thermal stability of human STING (R232) and mouse STING (R231) bound with HB3089 or diABZI (compound 3) in DSF assay. The data were shown as the mean values of d(RFU)/dt, *n* = 3. **d** HB3089 dose-dependently activated the ISG reporter in THP1-Dual cells after 24 h treatment. **e** The activation of ISG reporter by HB3089 (1 μM) was abolished in THP1-Dual-KO-STING cells after 24 h treatment. **f** HB3089 activated ISG reporter in RAW-Lucia cells after 24 h treatment. **g** The activation of ISG reporter by HB3089 (50 μM) was abolished in RAW-Lucia KO-STING cells after 24 h treatment. **h** Anti-tumor effect of HB3089 and diABZI (compound 3) in 4T1 breast tumor of Balb/c mice by intravenous (IV) administration on days 1, 4, 7, *n* = 6. **i** Anti-tumor effect of HB3089 in EMT6 breast tumor of Balb/c mice by IV administration, *n* = 6. Arrows indicate the days (1, 4, 7) of administration. ^**^P < 0.01, ^****^P < 0.0001, Two-way ANOVA. **j** EMT6 tumor growth in naïve mice and rechallenged mice, the number of EMT6 tumor-bearing mice and total mice were shown, respectively. **k**–**n** Frequency of CD86^+^DC, CD86^+^macrophages, GMZB^+^CD8T cells and IFNγ^+^CD8T cells in blood (24 h) and tumor tissues (24 h and 72 h) of 4T1 tumor-bearing Balb/c mice, *n* = 8. HB3089 was administrated by IV at 2 mg/kg. ns, not significant; ^*^P < 0.05, ^***^P < 0.001^,^ two-tailed Student’s *t-*test.

To design a new STING agonist with improved potency and drug-like properties, we started from dimeric amidobenzimidazoles (diABZIs), a representative of the most potent non-nucleotide STING agonists as reported by Ramanjulu and coworkers^30^. It has been noted that asymmetric diABZIs, with a morpholinopropoxyl substitution at one of two benzimidazole moieties in diABZIs, were more potent than symmetric diABZIs^30^, suggesting that the increase of diABZIs asymmetry could benefit the efficacy in STING activation. With these insights, we designed a new tricyclic scaffold by incorporating a furan ring into benzimidazole to replace one benzimidazole moiety in diABZIs (compound 3), leading to a more asymmetric new structure designated as HB3089 (Fig. 1a; Supplementary Fig. S1a).

Using differential scanning fluorimetry (DSF) assay, we found that HB3089 showed comparable ability to diABZI in increasing the thermal stability of various human STING isoforms and mouse STING (Fig. 1b, c; Supplementary Fig. S1b, c). Cellular studies further confirmed that HB3089 dose-dependently activated the interferon-stimulated gene (ISG) signaling in THP1-Dual reporter cells (Fig. 1d). This effect was abolished in STING knockout cells (Fig. 1e), suggesting a STING-dependent effect. HB3089 treatment activated the STING downstream TBK1/IRF3 signaling in dose-dependent and time-dependent manners (Supplementary Fig. S2a, b), associated with the increased production of cytokines IP-10 and IFN-β (Supplementary Fig. S2c, d). Further, the impact of HB3089 on two major human STING isoforms, STING-R232 and STING-H232, was examined in 293T-dual reporter cell lines overexpressing either STING-R232 or STING-H232, showing the wide activation on STING isoforms (Supplementary Fig. S2e, f). In addition to human STING, HB3089 also specifically activated mouse STING, as indicated by a reporter assay in Raw-Lucia and Raw-Lucia-KO-STING cells (Fig. 1f, g). These results demonstrated HB3089 is a highly specific STING agonist.

We next evaluated its anticancer activity in vivo. HB3089 showed a longer half-life (T_1/2_) and plasma exposure (AUC_0→∞_) than diABZI (compound 3) in mice (Supplementary Table S1), which may ensure its advantage over diABZI (compound 3) in anti-tumor activities. We found that HB3089, administered either by direct intra-tumoral or intravenous injection, exhibited a striking anticancer activity in a collection of allograft mouse models across different tumor types, including 4T1 and EMT6 breast cancer, B16F10 melanoma, CT26 colon cancer, LLC lung cancer, H22 liver cancer, U14 cervical cancer and RENCA renal cancer (Fig. 1h, i; Supplementary Fig. S3c, g, i, k, m, o), without causing apparent mice body weight loss (Supplementary Fig. S3a, b, d, h, j, l, n, p). HB3089 exhibited higher anti-tumor activity than diABZI (compound 3) in 4T1 breast cancer and U14 cervical cancer (Fig. 1h; Supplementary Fig. S3m). To test whether the therapeutic activity of HB3089 is due to STING activation, we compared its efficacy against B16F10 tumors implanted in wild-type (WT) and STING deficient mice (STING-KO) in parallel. Of note, the antitumor activity of HB3089 was completely abolished in STING-KO mice (Supplementary Fig. S3e, f), confirming a STING-dependent anticancer activity.

STING activation triggers CD8 T-cell-mediated antitumor immunity that benefits antitumor memory. We hence investigated whether HB3089 treatment strengthens the anti-tumor immune memory. To this end, mice being experienced a complete tumor regression by HB3089 treatment in both EMT6 and LLC tumor models were rechallenged with the same tumor cells after 2 months of tumor-free survival. We found that all the rechallenged mice rejected the tumor growth, in great contrast to those naïve mice (Fig. 1j; Supplementary Fig. S3q), suggesting that HB3089 enabled to induce anti-tumor immune memory.

Furthermore, we evaluated the impact of HB3089 on peripheral and tumor-infiltrated immune cells. As expected, the proportion of both peripheral and tumor infiltrated activated dendritic cells (CD86^+^DC) were increased in 4T1 allograft tumors treated with HB3089 (Fig. 1k). CD86^+^ macrophages were significantly increased in tumor after 72 h treatment with HB3089, while no significant increase observed in blood and tumor of 24 h treatment (Fig. 1l). In addition, monocytes and neutrophils in tumor tissues were increased as well (Supplementary Fig. S4a, b). Concordantly, peripheral and tumor infiltrated CD8^+^T cells (Supplementary Fig. S4c), as well as GMZB^+^CD8T and IFNγ^+^CD8T were increased by HB3089 treatment (Fig. 1m, n), suggesting the activation of antitumor immunity.

### Structure determination of HB3089-bound STING

**Fig. 2:**
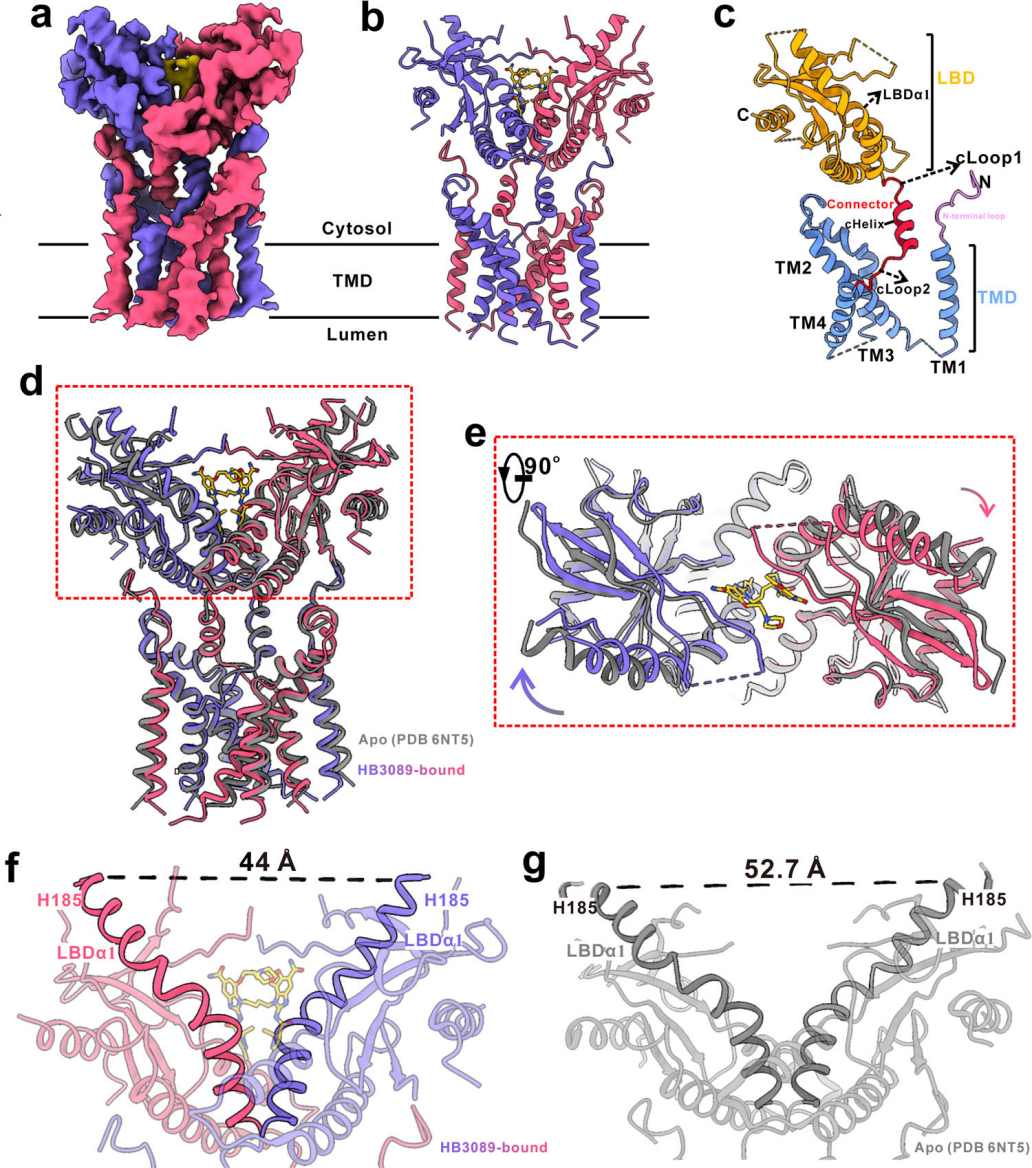
The HB3089-induced conformational changes of ligand-binding domain of STING. **a** A side view showing the density map of the HB3089-bound STING. The two monomers and the HB3089 are colored in purple, red and yellow, respectively. **b** A ribbon representation of the HB3089-bound STING. The two monomers and HB3089 are colored as in **a**. **c** The atomic model of a STING monomer. The LBD, the connector, the TMD and the N-terminal loop are colored in orange, red, blue and pink, respectively. The connector links LBDα1 of the LBD to the first transmembrane helix (TM1) of the TMD through the C- and N- terminal loop of the connector (cLoop1 and cLoop2, respectively). **d** Structural comparison between the apo full-length STING (PDB 6NT5) and the HB3089-bound full-length STING (this study). The HB3089-bound STING is colored as that in **b**. The apo STING is colored in grey. **e** A top view of the boxed region in **d**, showing the conformational change of the LBD. **f**, **g** The inter-Cα distance between residues His185 at LBDα1 of the two STING forms in **d**.

To understand how HB3089 activates STING, we took an approach of structural biology. Full-length human STING was purified and then incubated with excessive HB3089. We prepared the cryo-EM sample and collected the data of full-length human STING bound with HB3089 using a Titan Krios G3 electron microscope. Through data processing as illustrated in Supplementary Fig. S5a–c, we obtained a 3.47 Å reconstruction of the agonist-bound STING with local resolutions ranging from 3.0–5.0 Å (Supplementary Figs. S5d, S6). At this resolution, most of the residue side-chains were well resolved (Supplementary Fig. S6b), which were refined against the EM density map (Supplementary Table S2). The two monomers of the HB3089-bound STING interact intensively across all the three domains of the LBD, the TMD, and the connector (Fig. 2a–c). Each monomer of the connector consists of a helix (connector helix, cHelix) and two loops. The LBD and TMD are linked together through the two connector loops (cLoop1 and cLoop2) (Fig. 2c).

### Unique conformational changes of LBD and connector in HB3089-bound STING

We next illustrated how the conformational changes occurred upon HB3089 binding. The cryo-EM analysis revealed that the asymmetric agonist HB3089 rested into the pocket of the LBD (Supplementary Fig. S7a), which showed a two-fold symmetry appearance due to the imposed C2 average in the 3D auto refinement (Supplementary Fig. S7c). LBD bound to the agonist HB3089 mainly through hydrophobic interactions and hydrogen bonds (Supplementary Fig. S7d, e). While the hydrogen bonds between the Ser162 and Tyr240 on the first helix of LBD (LBDα1) and the nearby chemical groups of the agonist were identical to the isolated LBD bound with compound 2, a symmetrical diABZI (PDB 6DXL)^30^, the amino group of Val239 formed an extra hydrogen bond with the furyl ring of the agonist HB3089 (Supplementary Fig. S7e). Guided by the structure, we reconstructed several mutants (L159A, S162A, Y163A, Y167A, R238A, T263A, P264A), aiming to disrupt the interactions between the corresponding residues and HB3089. We found that these mutations remarkedly weakened the expression of the ISG reporter by STING activity assays (Supplementary Fig. S7f), indicating that these residues indeed played an important role for the binding of HB3089.

Previous studies have shown that the contraction of the isolated LBD induced by compound 2 is very subtle (Supplementary Fig. S8)^30,31^. However, the structural comparison revealed that the LBD in HB3089-bound STING contracts much more than the isolated one bound with compound 2, though most of interaction patterns are similar between the two structures (Supplementary Fig. S8e–g). Notably, incorporation of a furan ring in diABZI increased asymmetry in HB3089 and an extra hydrogen bond was formed between incorporated furan ring and Val239. As compared with the counterpart of the full-length human STING in apo state (PDB 6NT5), the two monomers of LBD from the HB3089-bound STING rotated inward towards the ligand-binding pocket (Fig. 2d, e), leading to a significantly contracted but open conformation, which was in contrast to the closed conformation induced by the cGAMP binding^25,32,33^. As such, the inter-Cα distances between residues His185 at LBDα1s C-terminal, which is used as the measurement of the LBD conformational change^23^, is reduced from 52.7 Å in the apo state to 44.0 Å in the HB3089-bound state (Fig. 2f, g). Meanwhile, the LBD of the full-length apo STING has a slightly more compact conformation than that of the isolated apo LBD (PDB 4EF5) (Supplementary Fig. S8). Put together, these results indicate that the LBD within the confine of full-length STING has the intrinsic force to push the two monomers away from each other, which, however, is countered by another force imposed by the other parts (the connector, the TMD and the N-terminal loop) of full-length STING. Upon binding with the ligand, this dynamic balance between the expansion and the contraction of the LBD is interrupted, leading to the contraction of the LBD.

**Fig. 3:**
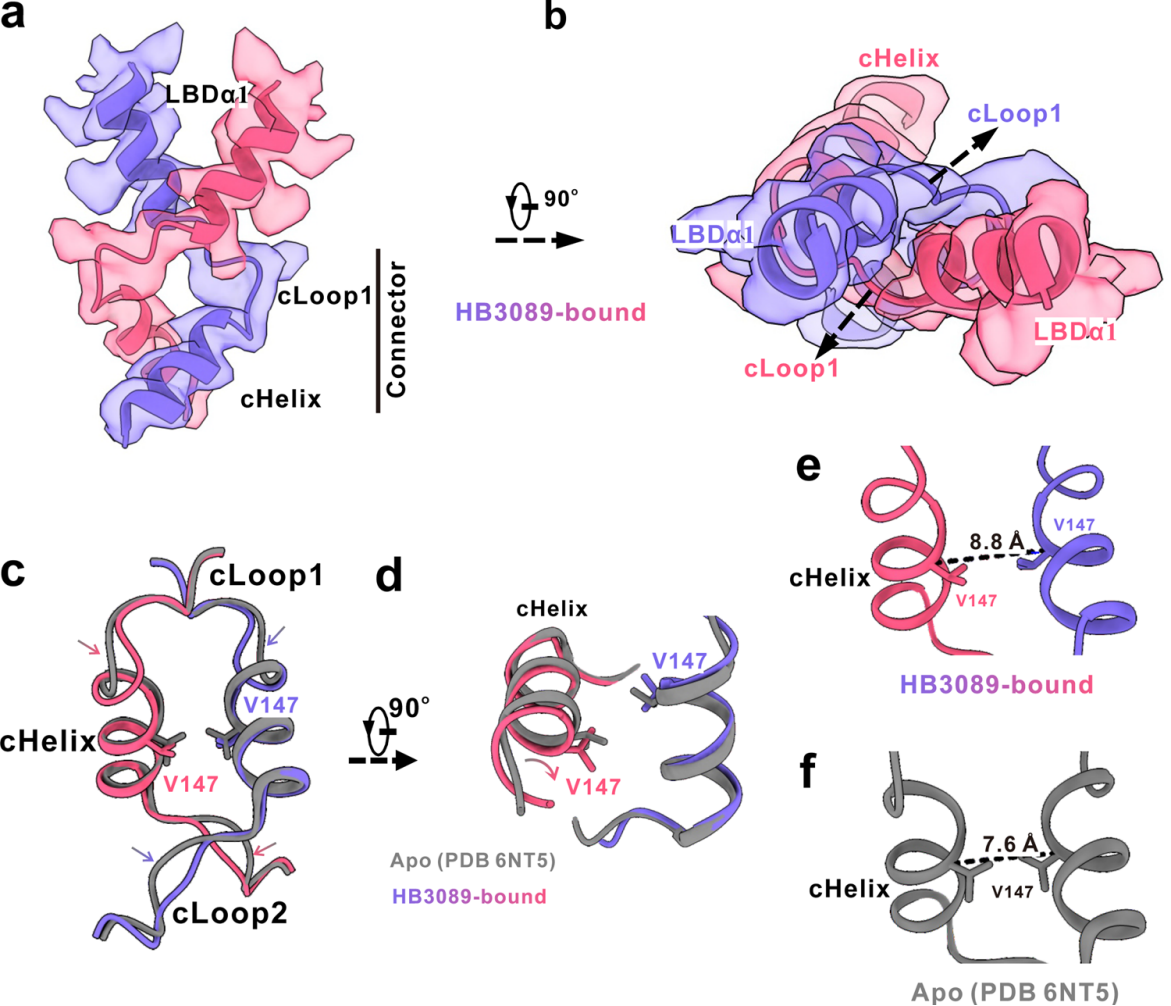
The conformation change of the connector induced by HB3089 binding. Side (**a**) and top (**b**) views of the connector from HB3089-bound STING, showing the two monomers of the connector and the LBDα1 form the right-handed crossover. For clarify, the cLoop2 of the connector is not shown. The map and the model are displayed in transparent surface and ribbon, respectively, and are colored as in Fig. 2b. **c**, **d** Structural comparison of the connectors between the HB3089-bound STING (red and purple) and the apo STING (grey), showing the helices and the loops of the connector from the HB3089-bound STING revolved (curved arrows) and moved downwards (arrows), respectively. The residues of Val147 are indicated by side chain showing. **e**, **f** The atomic models of the connector helices from the HB3089-bound STING (**e**, red and purple) and the apo STING (**f**, grey), showing the inter-Cα distance between residues Val147 at the connectors.

We further observed that the connector of the HB3089-bound STING remained the same right-handed crossover conformation as that of the apo human STING (Fig. 3a, b) rather than the un-crossover one of the full-length chicken STING bound with cGAMP^25^. This finding was not consistent with the conception that 180° rotation of LBD relative to TMD was necessary for STING activation, implying a distinctive STING activation mechanism by the agonist HB3089. Meanwhile, the two connector helices of the HB3089-bound STING revolved about 45^°^ on their own axis in comparison to that of the apo human STING (Fig. 3c, d), and the two helices slightly moved away from the two-fold axis. As such, the inter-Cα distance between residues Val147 on the connector helices increased to 8.8 Å from 7.6 Å (Fig. 3e, f). In addition, parts of the cLoop1 and the cLoop2 slightly move downwards (Fig. 3c, d).

### STING agonistic activation requires conformational change of the TMD

**Fig. 4:**
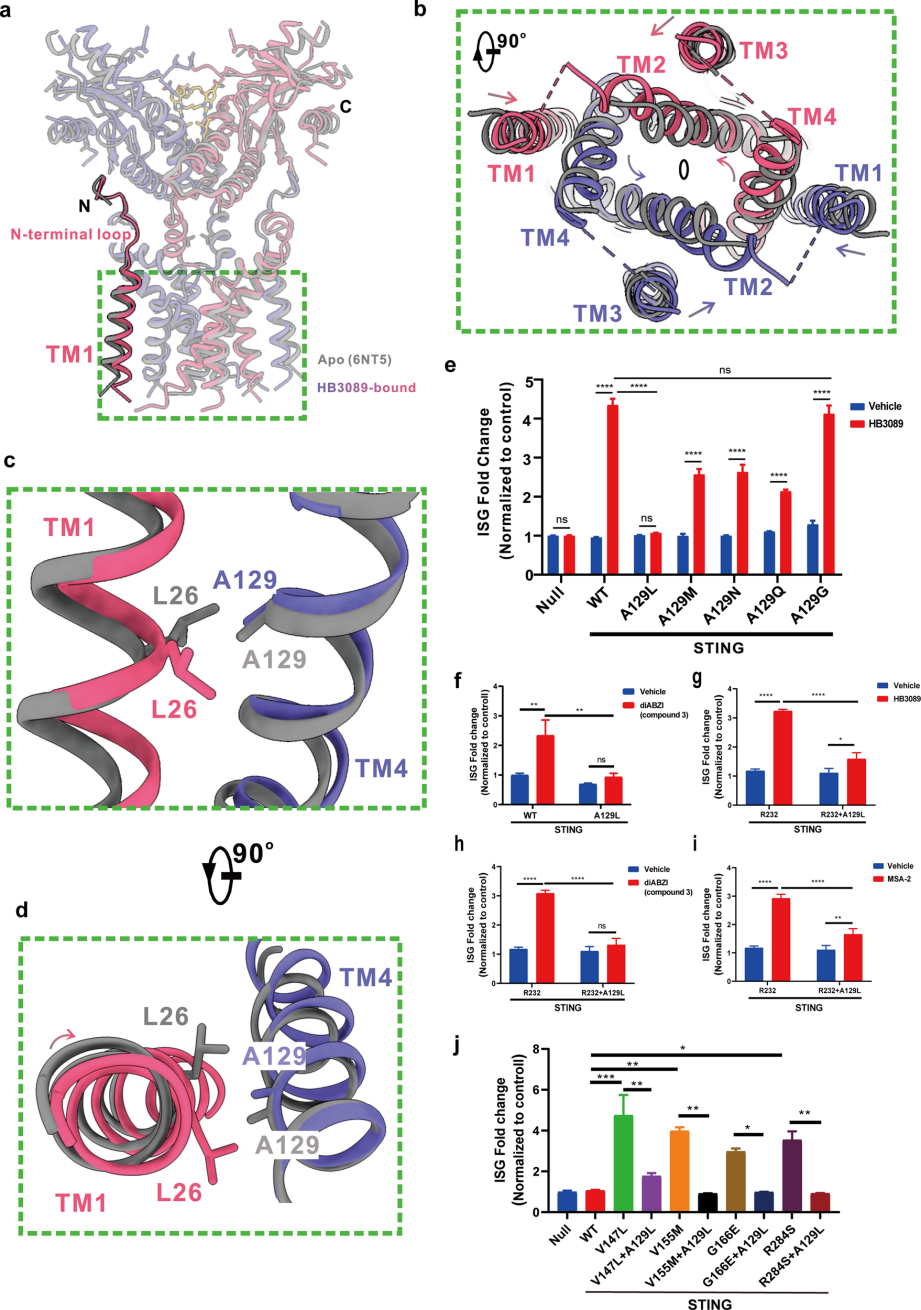
The conformational change of the TMD. **a** A structural comparison between the apo STING (PDB 6NT5) and the HB3089-bound STING (this study). The two STING forms are colored as in Fig. 2d. The TMD is indicated by the green box. The N-terminal loop and the TM1 are highlighted. **b** A top view of the boxed region in **a**. Each helix of the TMD rotated anticlockwise with somewhat different extents as indicated by the curved arrows. The TM1 and the TM4 also moved toward the two-fold axis (oval). The side (**c**) and top (**d**) views of the boxed view in **a**, showing that the TM1 revolved on its own axis as compared that of the apo STING. Obviously, the residue Leu26 on the TM1 from one monomer has to cross the Ala129 on TM4 from the other monomer during the process of revolution. For clarify, only the TM1 and TM4 are shown. **e**–**i** The effects of the STING residue A129 mutations on HB3089/diABZI (compound3)/MSA-2-induced expression of the ISG reporter. **j** The effects of the STING residue A129L mutation on SAVI-related mutations-induced expression of the ISG reporter. Data are presented as the means ± SD of triplicate experiments, significance is determined with One-Way ANOVA test; ns, not significant, ^*^P < 0.05, ^**^P < 0.01, ^***^P < 0.001, ^****^P < 0.0001.

The interruption of the dynamic balance between the expansion and the contraction of the LBD not only contributed to the aforementioned conformational changes of the LBD and the connector, but also triggered the structural change of the TMD. The two monomers of the TMD rotated anticlockwise, which is accompanied by the slightly inward movement of the outer transmembrane helices (TM1s and TM4s) towards the symmetry axis (Fig. 4a, b). In addition, the TM1s themselves revolved significantly on their own axis (Fig. 4c, d). It is worth noting that the N-terminal loop preceding the TM1 maintained the same hydrophobic contact with the surface of the LBD (Fig. 4a) as that of the full-length apo STING (PDB 6NT5)^25^. Therefore, the contraction of the LBD would put pressures on the TMD through the two N-terminal loops, which likely contributed to the conformational change of the TMD, particularly the inward movement of the TM1. Conversely, the conformational change of the TMD also likely affected the structural change of the LBD.

Previous studies have revealed that the translocation was necessary for the activation of STING^24,34–37^. We thus reasoned that the revolution of the TM1 might be prerequisite for STING activation. Since the residue Leu26 on the TM1 from one monomer had to cross the Ala129 on TM4 from another monomer during the process of the TM1 revolution (Fig. 4c, d), we mutated the Ala129 to amino acids with larger side chain to limit the revolution of the TM1. The ISG reporter analysis showed that the mutants (A129L, A129M, A129N, and A129Q) had much weaker HB3089-stimulated STING signaling than that of WT STING (WT, H232), whereas the mutant A129G, the mutation of which would not affect the revolution of the TM1 at all, showed a comparable HB3089-stimulated ISG signaling as WT did (Fig. 4e). Echoing with this, the STING mutant-A129L also showed weaker activation by diABZI (compound 3) as comparing to that of WT STING (Fig. 4f). Moreover, the STING mutant-A129L showed weaker activation by different STING agonist (HB3089, compound 3, MSA-2) in another common wide-type STING (WT, R232) (Fig. 4g–i). These results indicated the revolution of TM1 is critical for the activation by STING agonists. Next, we ask whether Ala129 mutation could affect the constitutive activation of SAVI-related mutations, such as V147L, V155M, G166E, R284S. As shown, A129L mutation reduced the constitutive activation of all these mutants (Fig. 4j), substantiating that the revolution of TM1 is also critical for the activation of SAVI-related mutations. Combined these findings, we concluded that the conformational changes of the TMD were indispensable for STING activation.

### The connector controls the conformational changes of the LBD and the TMD

**Fig. 5:**
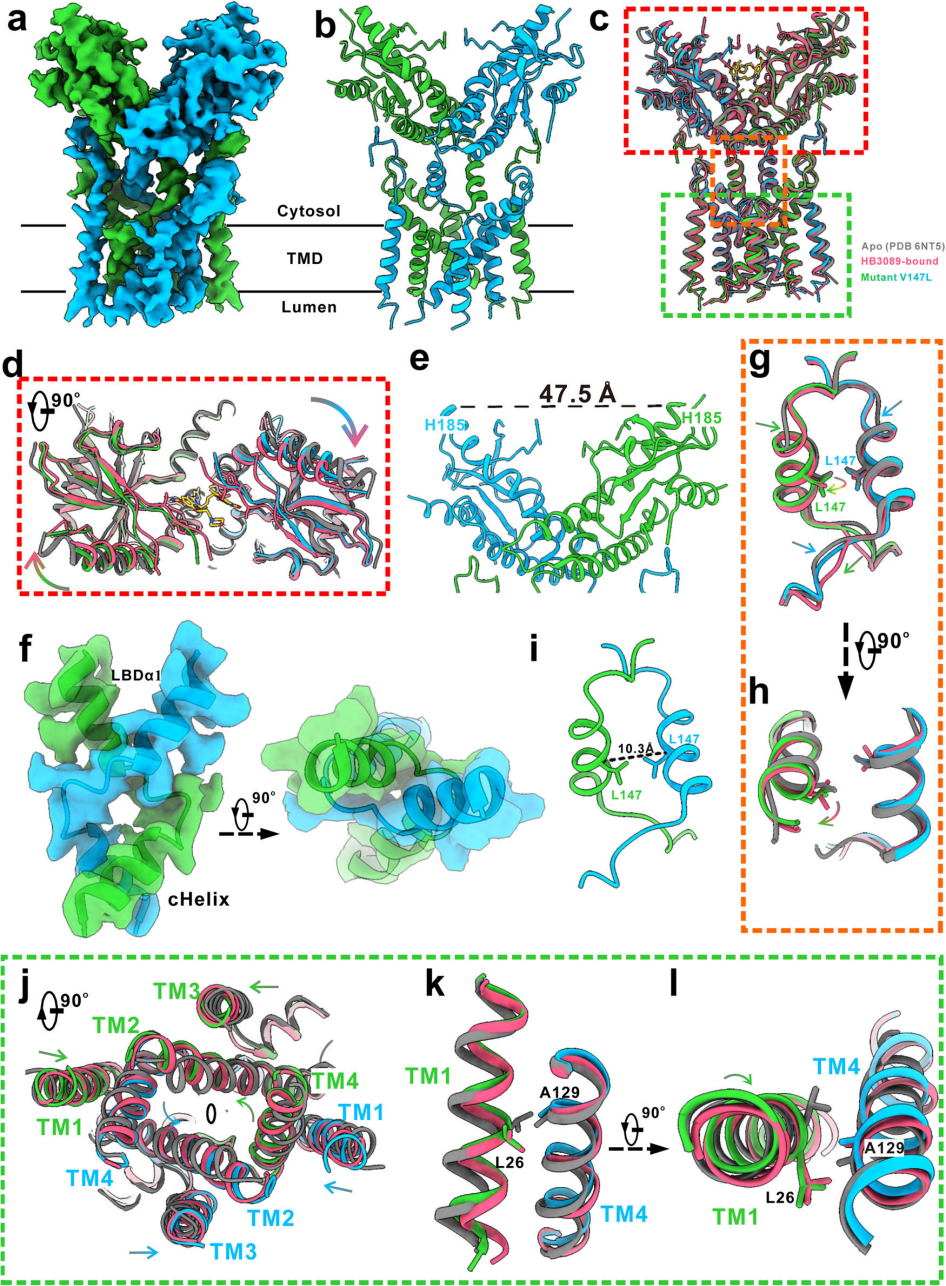
The conformational change of the SAVI-related mutant V147L. **a** The density map of the mutant V147L. The two monomers are colored in green and blue, respectively. **b** A ribbon representation of the mutant V147L. The two monomers are colored as in **a**. **c** Structural alignments of the apo STING (PDB 6NT5, grey), the HB3089-bound STING (this study, red), and STING mutant V147L (this study, blue and green). The proteins are displayed in ribbon. The HB3089 is in stick. **d** A top of the red boxed region in **c**, showing the LBD of the mutant is slightly less contracted than that of the HB3089-bound form. **e** The inter-Cα distance between residues His185 at LBDα1 of the mutant V147L. **f** Side (left) and top (right) view of the connector from HB3089-bound STING, showing the two monomers of the connector and the LBDα1 form the right-handed crossover. The map and model are in transparent surface and ribbon, respectively. Side view (**g**) and top (**h**) view of the orange boxed region in **c**, showing the helices and the loops of the connector from the mutant (green and blue) revolved (curved arrows) and moved downwards (arrows), respectively. The residues 147 are indicated by side chain showing. **i** The inter-Cα distance between residues Leu147 at the connector of the mutant V147L. **j** A top view of the boxed region in **c**. The conformational changes of the TMD of the mutant is essentially identical to that of the HB3089-bound form as describe in Fig. 2b. The side (**k**) and top (**l**) views of the boxed view in **c**, showing that the TM1 revolved on its own axis as compared that of the apo STING. It shows that the TM1 of the mutant V147L revolved on its own axis, which is similar to that of agonist-bound STING. For clarify, only the TM1 and TM4 are shown.

Given that the connector links the LBD and the TMD, and that many SAVI-related mutations bearing the constitutive translocation and signaling activities^22,23,38,39^ are located on the connector^22^, the conformational changes of the connector should play an important role for the structural changes of the LBD and the TMD. To test this possibility, we then resolved the cryo-EM structure of the SAVI-related mutant V147L at a resolution of 3.65 Å (Fig. 5a, b; Supplementary Figs. S9, S10). The major differences between the two STING forms were the extents of the structural changes of the LBD and the connector (Fig. 5c–i), whereas that of the TMDs were essentially identical at the current resolutions (Fig. 5j–l). First, the contraction of the LBD in the mutant was slightly smaller, as indicated by the larger inter-Cα distance (47.5 Å) between residues His185 as compared with that (44 Å) of HB3089 bound STING (Figs. 2f, 5e). Second, the revolution of the connector helices in the mutant was more obvious than that of the HB3089 bound STING, as indicated by the larger inter-Cα distance (10.3 Å) between residue 147 than that (8.8 Å) of the HB3089 bound STING (Figs. 3e, 5i). However, all the three domains of the SAVI-related mutant, including the LBD, the connector and the TMD, showed overall conformational change similar to that of the full-length STING bound with HB3089, implying a shared activation mode: the connector senses the activation signals and controls the conformational changes of the LBD and the TMD.

**Fig. 6:**
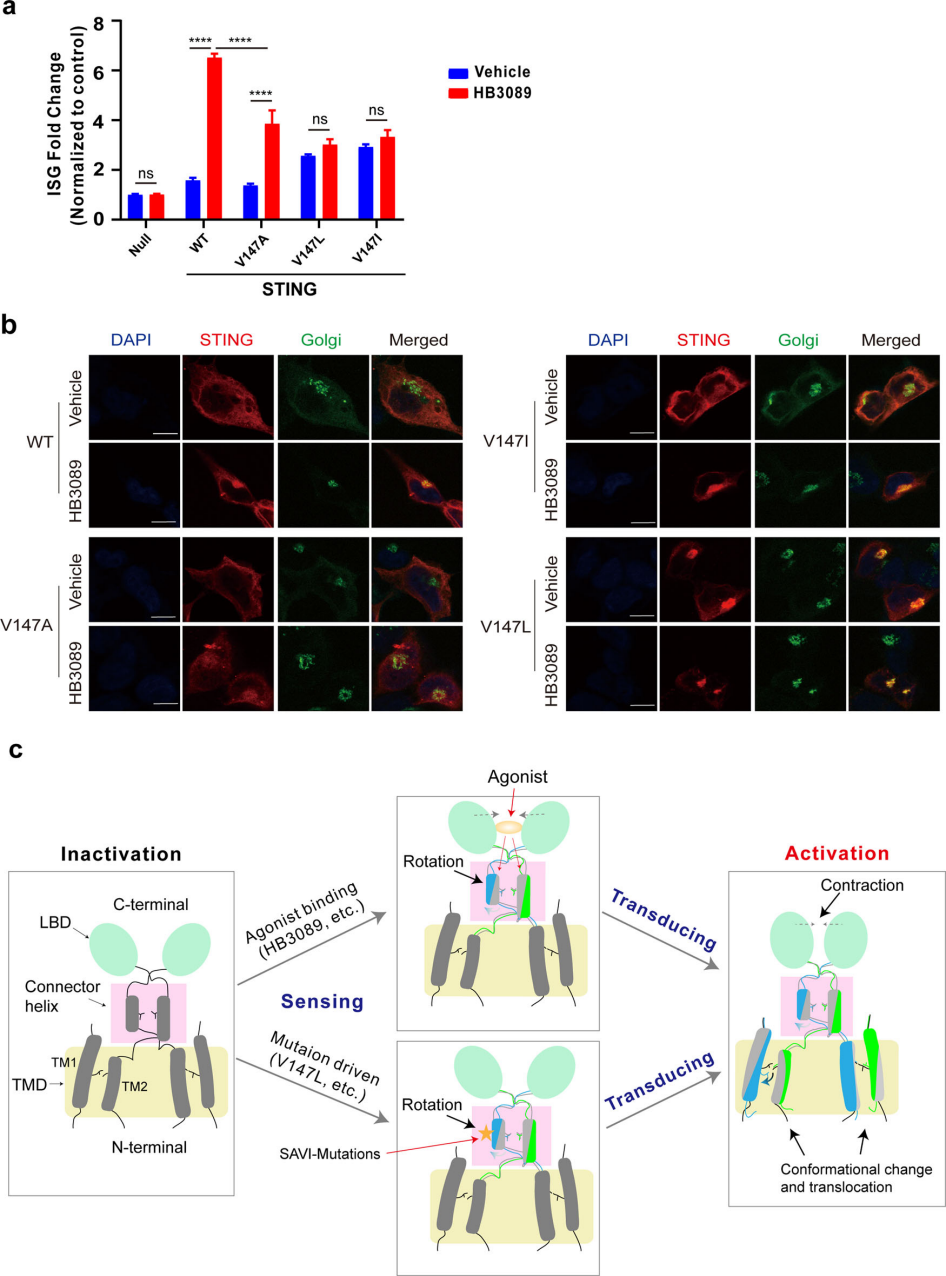
The effect of the sidechain size of residue 147 on STING activation. **a** The effects of the residue V147 mutations on HB3089-induced expression of the ISG reporter. Data are presented as the means ± SD of triplicate experiments, significance is determined with One-Way ANOVA test; ns, not significant, ^****^P < 0.0001. **b** The effects of the residue V147 mutation on the translocation of STING using confocal immunofluorescence assay. 293 T cells were stained with anti-STING to label STING (red), anti-GM130 (green) to label Golgi, and DAPI (blue) to label the nuclei. Scale bar: 10 μm. Representative images were shown from three independent experiments. **c** Schematic summarizing STING activation mechanism shared by HB3089 and SAVI-related mutant V147L. The connector helix senses the STING-activating signal including HB3089 binding (oval) or SAVI mutation (star) and rotates driving by the steric hindrance of the connector residues, which eventually leads to the significant contraction of the LBD and the conformational changes, especially TM1 revolution, of the TMD for STING activation. The connector and transmembrane helices in the inactive state are colored by grey for both monomers, while they are colored by blue and green for monomers respectively.

It is worth noting that the inter-Cα distance between residues 147 in the apo STING is so small that there is no room for residues with larger side chains (Fig. 3e). Therefore, the conformational change of the connector helix from the SAVI-related mutant must be large enough to eliminate the clash between the two larger residues of leucine. The intrinsic force of the connector of the mutant, which is somewhat correlated with the steric hindrance of the connector residues, is enough to drive the structural changes of the LBD and the TMD through the revolution of the connector helices. The revolution of the connector helices has two effects: avoid the clash between the two residues of leucine on one hand, and release the intrinsic force of the connector on the other hand. As in the case of WT, the intrinsic force of the connector is not enough to do so. Once with the help of the extra force produced by the agonist binding to the LBD, however, the intrinsic force of the connector is able to promote the conformational changes of the LBD and the TMD. We believe that the intrinsic force of the connector in the WT also comes from the steric hindrance of the connector residues. To further confirm the proposed mechanism, we carried out structure-based mutagenesis studies. First, we mutated Val147 to Ile that has larger side chain. The ISG reporter analysis showed that the mutant V147I has constitutive STING signaling, same as the mutant V147L (Fig. 6a). In addition, fluorescence colocalization study showed that the mutant V147I also has constitutive translocation activity (Fig. 6b). Second, we mutated Val147 to Ala that has a smaller side chain. The mutant V147A has no constitutive translocation and signaling activities (Fig. 6a, b). Furthermore, even under the condition with HB3089 existence, the mutant V147A shows a significantly lower activity than that of the WT (Fig. 6a). Consistently, the HB3089-stimulated translocation activity of the mutant V147A is weaker than that of the WT (Fig. 6b). These results indicate that the connector, through structural changes of the connector helices to prevent or reduce the steric hindrance, controls the conformational changes of the LBD and the TMD.

## Discussion

In the present study, we took an approach of rational design based on diABZI compounds and obtained a more asymmetric diABZI structure, HB3089. HB3089 could bind to various STING isoforms and specifically activated STING downstream signaling. HB3089 displayed the improved pharmacokinetic profile and potent anti-tumor activity in multiple tumor models of immune-competent mice by either systemic or local administration, associated with the activation of anti-tumor immunity. The anti-tumor activity of HB3089 could be abolished in STING-KO mice, confirming a STING-dependent anticancer activity. All these suggest that HB3089 is a potent STING agonist and the compound is currently undergoing preclinical development.

The currently recognized activation mechanism of STING was mainly based on the previously reported structures of the full-length chicken STING^25^. It has been revealed that the LBD in chicken STING rotated 180° relative to the TMD when STING is converted from the inactive to the active state, which was the prerequisite for STING activation. However, both structures of activated human STING obtained in this study did not show a 180° rotation of the LBD, implying that the rotation of the LBD relative to the TMD was dispensable for STING activation. The recent structure of human STING bound to the compound 53 in the active conformations shows a 180° rotated conformation of the LBD, suggesting that the 180° rotation mechanism is not species specific^40^. Given that the conformational change of the autoimmune-related mutant V147L is similar to that of agonist-bound STING, our results reveal a conservative mode for STING activation, which would revise or at least complement the current paradigm for the activation mechanism of human STING.

The distinct activation mode demonstrated in this study indicated the necessity of the conformational change of the TMD for STING activation, which was featured by the N-terminal transmembrane helix revolving significantly on its own axis. Noteworthy, a very recent study reported that compound 53 binds to the TMD and enhances the ability of cGAMP to activate STING^40^.

A previous crystal structure shows that the two monomers of LBD undergo asymmetric change upon binding with cyclic di-GMP^31^. To explore whether the full-length HB3089-bound STING undergoes the asymmetrically conformational change, we obtained the 3.72 Å C1 structure of the HB3089-bound STING (Supplementary Fig. S11c), which clearly displayed the asymmetric density of HB3089 as indicated by the protruding 3-morpholinopropyloxy group only resting on one side of the agonist (Supplementary Fig. S11e). Intriguingly, the density map and local B-factor analysis of the HB3089-bound STING showed that the outer transmembrane helices were much more flexible, an indication of vibration, as compared with the inner ones, and this vibration seems of asymmetric (Supplementary Fig. S11d). Likewise, the 3.75 Å C1 structure obtained from the SAVI-related mutant V147L also exhibited the asymmetric vibration of the outer transmembrane helices that is similar to that of the HB3089-bound STING (Supplementary Fig. S11g), substantiating that the asymmetric vibration should be the intrinsic feature of STING. Interestingly, the outer transmembrane helices in the agonist-binding form had obviously higher B*-*factor (Supplementary Fig. S11f, h), together with the fact that agonist-stimulated STING signaling was stronger than that of the mutant V147L, empowering a presumption that the vibration of the outer transmembrane helices may play a role in the activation of STING (Fig. 6c).

Upon binding with agonists, STING translocates from the ER to the Golgi, where STING forms oligomer and a conserved PLPLRT/SD motif within the C-terminal tail of STING binds to the dimer interface of TBK1, then TBK1 phosphorylates the tails of neighboring STING molecules^26,27^. Phosphorylation of STING at the ^363^LXIS^366^ motif constitutes an IRF3-binding motif and allows STING to recruit IRF-3, which brings IRF-3 and TBK1 into proximity and thus facilitates activation of IRF-3^11,41^. Based on our results and the existing literatures, the process of STING activation contains two distinct steps: the first step is the translocation from the ER to the Golgi; the second step is the polymerization of STING for recruiting and activating TBK1. Recent studies have shown that STING aggregated in the Golgi apparatus is self-activating independent of agonists^15,42,43^. Glycosaminoglycans in the Golgi apparatus are necessary and enough to drive STING polymerization and activation^24^. These studies indicate that the role of agonists should be to promote STING translocation rather than to promote the polymerization of STING dimer as proposed by two previous structural studies^23,25^. Consistently, our results show that the agonist induces the conformational change of the TMD, and this change is necessary for the translocation. Previous study showed that STING is associated with the ER-resident protein stromal interaction molecule 1 (STIM1) through their transmembrane domains, which contributes to the ER-retention of STING^44^. Either induced by agonists or caused by the SAVI-related mutation, the conformational change of the TMD, including the vibration of the outer helices, likely facilitate the disassociation of STING from STIM1, ultimately leading to the translocation and polymerization of STING.

In summary, this study developed a potent STING agonist HB3089 showing potent and durable anti-tumor effect against a variety of tumors. By analyzing the structural changes of HB3089-bound STING, we revealed a new working model of STING activation that similarly occurs in some clinically relevant gain-of-function STING mutants. Our study provides useful structural information for the rational design of therapies for treating cancers or autoimmune disorders, through promoting or limiting the conformational changes of the TMD of STING.

## Materials and methods

THP1-Dual cells (Cat. code: thpd-nfis), THP1-Dual KO-STING cells (Cat. code: thpd-kostg), 293T-Dual hSTING-H232 cells (Cat. code: 293d-h232), 293T-Dual hSTING-R232 cells (Cat. code: 293d-r232), Raw-Lucia (Cat. code: rawl-isg) and Raw-Lucia-KO-STING (Cat. code: rawl-kostg) were purchased from InvivoGen (San Diego, USA). All the cells were incubated following manufacturer’s protocols at 37 ^o^C in a humidified atmosphere of 5% CO_2_. QUANTI-Luc solution (Cat. code: rep-qlc2) and QUANTI-Blue solution (Cat. code: rep-qbs2) were purchased from InvivoGen (San Diego, USA). Antibodies against phospho-TBK1 (Ser172) (Cat. code: 5483S), TBK1 (Cat. code: 3504S), phospho-IRF3 (Ser396) (Cat. code: 4947S), IRF3 (Cat. code: 4302S), and GAPDH (Cat. code: 5174S) were purchased from Cell Signaling Technology (Beverly, MA). diABZI-compound 3 (Cat. code: S8796) and MSA-2 (Cat. code: S9681) were purchased from Selleck (Houston, USA).

### Differential scanning fluorimetry (DSF)

DSF assay was used to evaluate the binding of compound to STING protein. Cytosolic fragment (140-379) of human STING protein (R232, H232, 293Q, AQ) and fragment (139-378) of mouse STING (R231) were obtained from NOVOPROTEIN (Shanghai, China). SYPRO Orange protein gel stain (5000×) (Cat.code: S6651, invitrogen), STING protein (5 μM) and compound (50 μM) were mixed in the 40 μL of reaction buffer which containing 10 mM HEPES (pH 7.5) and 150 mM NaCl. Then reaction mixture was transferred to a 96-well plate and measured by CFX96 Real-Time System (Bio-Rad, CA, USA). Briefly, HEX fluorescence signal was monitored as run program, the initiate temperature at 25 °C, followed by a temperature gradient in which the samples are heated at a scan rate of 0.5 °C per minute until to a final temperature of 80°C. Export the melt curve derivative result using the miner delta fluorescence unit’s data to generate the figures, and the thermal shift (ΔTm) for HB3089 or diABZI-compound 3 was calculated by the differential average denaturing temperature of the STING with vehicle from the average denaturing temperature of the STING-HB3089 or STING-diABZI (compound 3) complex.

### ISG reporter assay

THP1-Dual cells or THP1-Dual KO-STING cells (1 × 10^5^ cells/well), 293T-Dual hSTING-H232 or 293T-Dual hSTING-R232 (0.8 × 10^5^ cells/well), Raw-Lucia or Raw-Lucia-KO-STING cells (0.8 × 10^5^ cells/well) were suspended in 180 µL medium and added to 96-well plates, then indicated concentrations of compound (20 μL) was added to the cells for 24 h. QUANTI-Luc or QUNTI-Blue detection reagent were reconstituted and utilized following manufacturer’s protocol to measure luciferase or secreted embryonic alkaline phosphatase (SEAP) of cell supernatant. The luminescence and absorbance at 650 nm were measured on the SPARK Multimode microplate reader (TECAN) and SpectraMAX plus 384 (Molecular Devices, Sunnyvale, CA), respectively. The ISG-fold change was calculated relative to the vehicle control.

### Western blotting

THP1-Dual (8 × 10^5^ cells/well) were seeded into a 6 well plate and treated with HB3089 (100 nM) for different times or doses of HB3089 or vehicle as indicated. Cell lysates were harvested by 1× SDS-PAGE Sample Loading Buffer (Cat. code: P0015, Beyotime, China), boiled at 100 °C for 30 min. Cell lysates were run on 10% SDS-PAGE gels and transferred onto NC membranes. The membranes were blocked with 5% BSA for 1 h and incubated with antibodies against TBK1, phospho-TBK1 (Ser172), IRF3, phospho-IRF3 (Ser396) or GAPDH for overnight at 4 °C. Then, membranes were incubated with horseradish peroxidase-conjugated secondary antibodies. Finally, the membrane was detected using enhanced chemiluminescence (ECL Plus, Cat. code: 17050622, BioRad).

### Enzyme-linked immunosorbent assay

THP1-Dual cells were seeded in 96-well plates at a density of 1 × 10^5^ cells per well and treated with indicated concentrations of HB3089 for 24 h. Cell supernatants were collected, and the levels of cytokines were determined by human IP10 kit (Cat. code: 550926, BD) or human IFN-β kit (Cat. code: 70-EK1236-96, MultiSciences) according to the manufacturer’s instructions. The cytokine concentration (pg/mL) was calculated according to the standard curve. The test was carried out in triplicates.

### Animal studies

Animal procedures were approved by the Institutional Animal Care and Use Committee of the Shanghai Institute of Materia Medica. Balb/c, C57BL/6, and Kunming female mice (6-week-old) were purchased from LingChang Biotechnology (Shanghai, China). C57BL/6 mice with the stable knockout of the STING gene were purchased from MODEL ORGANISMS (Shanghai, China). B16F10 cells (0.5 × 10^5^), 4T1 (5 × 10^5^), EMT6 (2.5 × 10^5^), CT26 cells (2.5 × 10^5^), RENCA (2.5 × 10^5^), LLC (2.5 × 10^5^), U14 (5 × 10^5^), H22 (2.5 × 10^5^) were injected subcutaneously into the right armpit of each mouse. When the tumors grew to approximately 50–100 mm^3^, the mice were assigned into vehicle control groups and treatment groups randomly (*n* = 6–8). The mice were intermittently treated with compounds by intravenous (IV), intraperitoneal (IP) or intratumoral (IT) injection as indicated, and vehicle control was given with 40% PEG400.

EMT6 and LLC rechallenge model: the mice bearing EMT6 or LLC tumors that completely regressed after compound HB3089 treatment, after 2 months of tumor-free survival, were reinoculated with same tumor cells, and naïve mice were used as control.

Tumor volume and body weight were measured twice per week. Tumor volume (TV) was calculated as the formula: V = (a × b^2^)/2 (a, length; b, width).

### Flow cytometry analysis

4T1 breast tumor was established in BALB/c mice. When the tumor volume grew to around 400 mm^3^, mice were treated with vehicle (40% PEG400) or HB3089 (2 mg/kg) for 24 h and 72 h, then mice were sacrificed, and the blood and tumor tissues were collected. The blood samples were lysed by 1× RBC Lysis Buffer (10×, Cat. code 420301, Biolegend). The tumor samples were excised scissors and then digested in digestion buffer (RPMI-1640, 0.1% IV collagenase, 0.01% Dnase). Purified single cells were obtained and passed through a 70-μm strainer and followed by lysis of red blood cells. To identity live cells, cells were stained with LIVE/DEAD cell stain kit (BD, 564407/565388) for 10 min in the dark. Cells were resuspended with FACS buffer and stained with surface antibodies for 30 min on ice. Including antibodies: CD3 (BV510 Cat. code: 563024, BD Bioscience), CD4 (BUV737 Cat. code: 741704, BD Bioscience), CD8 (BUV395 Cat. code: 563786, BD Bioscience), CD11b (BUV395 Cat. code: 563553, BD Bioscience), Ly-6G (PE Cat. code: 551461, BD Bioscience), Ly-6C (Percp-CY5.5 Cat. code: 560525, BD Bioscience), and CD86 (PE-CY7, Cat. code: 105014, Biolegend). For intracellular protein staining, cells were fixed, permeabilized (Cat. code: 562574, BD Bioscience), and stained with antibodies against intracellular molecules, including antibodies against Granzyme B (BV421 Cat. code:396414, Biolegend), and IFNγ (percy5.5 Cat. code: 505822, Biolegend). Next, cells were fixed with 4% PFA in the dark for 15 min, then individual samples were washed and resuspended in FACS buffer. Finally, samples analyzed by using BD LSRFortessa, and the raw data was processed by Flowjo software.

### Protein expression and purification

The coding sequence of human full-length STING was cloned into the pCAG vector with a carboxy-terminal Flag tag. HEK293F cells (Thermo Fisher Scientific) were maintained in Freestyle 293 medium (Thermo Fisher Scientific) at 37 ^o^C with 5% CO_2_ and used for the overexpression of STING. When cell density reached 2.5 × 10^6^ cells/mL, the cells were transiently transfected with the expression plasmids using polyethylenimines (PEI, Polysciences). In details, about 1 mg expression plasmids were pre-mixed with 3 mg PEI in the fresh culture medium and incubated for 15 min, and the mixture was then added to 1 L of cell culture. After 16 h post-infection, 10 mM sodium butyrate was added to the culture medium and the culture temperature was shifted to 30 °C. Cells were collected 60 hours after infection for the purification of STING.

Cell pellets were resuspended in the buffer containing 20 mM HEPES (pH 7.4), 150 mM NaCl and protease inhibitor cocktail (Bimake). The cells were lysed by high pressure homogenization and centrifuged at 8000× *g* at 4°C to remove the cell debris. The supernatant was further centrifuged at 100,000× *g* for 30 min at 4 °C to enrich the membrane fraction. The membrane pellet was then solubilized using 20 mM HEPES (pH 7.4), 150 mM NaCl, 0.5% (w/v) lauryl maltose neopentylglycol (LMNG, Anatrace) and 0.1% (w/v) cholesteryl hemisuccinate (CHS, Anatrace) for 2 h at 4 °C. Insolubilized material was removed by ultracentrifugation at 100,000× *g* for 30 min and the solubilized fraction was immobilized by batch binding to Flag affinity resins (Genscript Biotech). After that, the resins were packed and washed with washed with 20-column volumes of 20 mM HEPES (pH 7.4), 150 mM NaCl, 0.01% (w/v) LMNG, and 0.002% (w/v) CHS. The target protein captured by affinity resins was then eluted in the buffer containing Flag peptide and concentrated using an Amicon Ultra Centrifugal Filter. The concentrate was subjected to size-exclusion chromatography (SEC) on a Superdex 6 Increase 10/300 column (GE Healthcare) equilibrated with buffer consisting of 20 mM HEPES (pH 7.4), 150 mM NaCl, 0.00075% LMNG, 0.00025% glyco-diosgenin (GDN, Anatrace) and 0.0002% CHS to separate target protein from contaminants. Fractions from SEC were evaluated by sodium dodecyl sulfate polyacrylamide gel electrophoresis and those containing target protein were pooled and concentrated for cryo-EM experiments.

### Cryo-EM grid preparation and data acquisition

Peak fractions collected from SEC were concentrated to 15 mg/mL and 3 µL of the purified sample were applied onto a glow-discharged Quantifoil R1.2/1.3 300-mesh gold holey carbon grid. The grids were blotted for 3 s under 100% humidity at 4 °C and then plunge-frozen in liquid ethane cooled by liquid nitrogen using a Vitrobot Mark IV (Thermo Fisher Scientific).

Movies were collected on a 300 kV Titan Krios (FEI) equipped with a Gatan image filter and a K3 Summit detector (Gatan). SerialEM was used to automatically acquire movies at a calibrated pixel size of 1.071 Å and defocus values ranging from −1.5 μm to −3 μm. Each movie with 40 frames was collected at a total dose of 70 e−/Å^2^ over an exposure time of 3 s. Totals of 7906 and 25782 movies for the WT STING bound with HB3089 and the SAVI-related mutant V147L were collected, respectively.

### Image processing and 3D reconstruction

All 40 frames in each movie were aligned and dose weighted using MotionCor2^45^. Gctf was used for estimating the defocus values and astigmatism parameters of the contrast transfer function (CTF)^46^. Micrographs were chosen for further processing. About 8000 particles were initially picked by ManualPick in RELION 3.0^47^ from selected micrographs. The particle images binned 2 times (128 × 128) were extracted with a pixel size of 2.142 Å and were subjected to reference-free 2D classification. The good reference-free 2D classes were selected and used as templates for automated particle picking. Totals of 8293361 and 19777436 particles for the WT STING bound with HB3089 and the SAVI-related mutant are picked, respectively. These picking particles were extracted and processed with 2D classifications. An initial model for 3D classifications was generated de novo from selected particles using the stochastic gradient descent algorithm with low pass filtered to 30 Å and was used as the reference model for 3D classification. In the 2D or 3D classification, those classes without high-resolution and interpretable features were considered as “bad class” and were discarded. Finally, particles from a 3D class showing the best secondary structural features in the transmembrane domain were selected for further 3D refinement. The overall resolution was estimated based on the gold-standard FSC at 0.143 criterion. The local resolution was calculated by ResMap^48^. C2 symmetry was imposed during 3D classifications and refinements otherwise indicated.

The C1 structure determination of the HB3089-bound STING was illustrated in Supplementary Fig. S11a. Briefly, the particle dataset used for the final C2 reconstruction was expanded with 2-fold symmetry. The symmetry-expanded dataset was then subjected to a round of 3D classification without alignment using a mask encompassing only the STING molecule and ligand generated from the C2 map. One of the three converged classes, accounting for 43.51% of the symmetry-expanded dataset, was selected for further process. After removing the redundant particles using the flag _rlnMaxValueProbDistribution in particle.star file, we finally obtained the C1 reconstruction of HB3089-bound STING at a resolution of 3.72 Å from a total of 46,810 particle images (Supplementary Fig. S11c, d). Unlike the C2 reconstruction that shows the two-fold symmetrically arranged densities of the 3-morpholinopropyloxy group of HB3089 (Supplementary Fig. S6b, c), the C1 map shows the 3-morpholinopropyloxy group was located only at one side of the reconstruction (Supplementary Fig. S11e).

The C1 structure determination of the mutant V147L was similar to that of the HB3089-bound STING (Supplementary Fig. S11b). The symmetry-expanded dataset was subjected a round of 3D classification without alignment using a mask encompassing only the STING molecule generated with the C2 map. One of the four converged classes, accounting for 34.07% of the symmetry-expanded dataset, was selected for further process. After removing the redundant particles, we finally obtained the C1 reconstruction of the mutant at a resolution of 3.75 Å from a total of 35,428 particle images (Supplementary Fig. S11b, c, g). The density map of the outer transmembrane helices of the mutant C1 reconstruction was asymmetrically arranged in a way similar to that of the HB3089-bound STING (Supplementary Fig. S11f, h).

### Model building and refinement

The atomic structure of human STING in the apo state (PDB 6NT5) was docked into the final density map using Chimera^49^ and was used as an initial template for model building. The model was then subjected to several rounds of manual adjustment and refinement using Coot^50^ and Phenix^51^, respectively. The model statistics were validated using Molprobity^52^. All of the structural figures were prepared using Chimera^49^, ChimeraX^53^, and PyMOL (https://pymol.org/2/).

### Constructs for cell-based assays

The STING gene was cloned into pUNO1 vector (Cat. code: puno1-hstingwt, InvivoGen) without any tags. All the mutations used in this study were created through standard PCR-based mutagenesis method and confirmed by DNA sequencing. The resulted plasmids containing WT or mutated STING genes were used for cell-based assays.

### Cell transfection and STING activity assay

293T-Dual-Null cells (Cat. code: 293d-null, InvivoGen) contain a secreted embryonic alkaline phosphatase (SEAP) as an ISG reporter under the control of interferon regulatory factor (IRF). The cells were plated into 6-well at density of 5 × 10^5^/well and grew to 70%–90% confluency at transfection. Dilute 5 μL Lipofectamine 3000 Reagent (Cat. code: L3000015, Invitrogen) into 125 μL Opti-MEM Medium and mixed. Meanwhile, prepare master mix of each plasmid containing the WT or mutated STING gene by diluting 2.5 μg of each plasmid in 125 μL Opti-MEM Medium, then add 5 μL P3000 Reagent (Cat. code: L3000015, Invitrogen) and mix. Add each diluted plasmid to diluted Lipofectamine 3000 Reagent (1:1 ratio) and incubate for 15 min at room temperature to form DNA-lipid complex. Add each plasmid-lipid complex (250 μL) to 293T-Dual-Null reporter cells and incubate for 6 h at 37 °C, then replace the supernatant with 2 mL fresh DMEM medium containing 10% FBS. After 48 h incubation, cells (2 × 10^4^ cells/well) were suspended in 180 μL medium and added to 96-well plates, then 20 μL of HB3089 (100 nM) or diABZI (compound 3) (100 nM) or MSA-2 (50 μM) or vehicle (0.01% DMSO) was added to the cells for 24 h. Add 20 μL of cell supernatant to 180 μL of QUANTI-blue solution per well in a flat-bottom 96-well plate and the absorbance at 650 nm was measured by Spectramax Plus 384 (Molecular Devices). The ISG-fold change was calculated relative to the vehicle control group.

### Immunofluorescence assay

The 293 T cells (Cat. code: CRL-3216, ATCC) were transfected with human STING or its mutated residues for 48 h, the protocol was the same as indicated above. Then, cells were transferred to 24-well plates at 1.2 × 10^5^ per well on poly-lysine coated coverslips. On the next day, cells were treated with HB3089 (100 nM) or vehicle (0.1% DMSO) for 2 h. After the treatment, cells were washed with phosphate-buffered saline containing 0.05% Tween 20 (PBST) and then fixed with 4% paraformaldehyde for 15 min at room temperature. Being washed again with PBST on a shaker for 5 min, cells were permeabilized for 20 min in 0.25% Triton X-100 in PBST and then washed three times in PBST for 5 min each time on a shaker. Subsequently, cells were put into blocking buffer, which is comprised of 3% bovine serum albumin (BSA) in Tris-buffered saline with 0.2% Tween 20 (TBST), for 1 h at room temperature. Cells were incubated with primary antibodies including anti-STING rabbit monoclonal antibody (1:100, Cat. code: ab239074, Abcam, USA) and anti-GM130 mouse polyclonal antibody (1:200, Cat. code: ab169276, Abcam, USA) at 4 °C overnight, which diluted in TBST with 3% BSA. The following day cells were wash three times with TBST, and then incubated with secondary antibodies at room temperature for 1 h and DAPI staining solution (Cat. code: C1005, Beyotime, China) for 30 min after three times washes with TBST. The secondary antibodies including cy3 anti-rabbit IgG-H&L (1:200, Cat. code: AS007, Abclonal, China) for labeling STING and FITC anti-mouse IgG-H&L (1:200, Cat. code: AS001, Abclonal, USA) for labeling GM130 (Golgi). Cells were sealed with anti-fade mounting medium (Cat. code: P0128S, Beyotime, China) and images were acquired using Lecia TCS SPS CFSMP with a 100× oil-immersed objective.

## Supplementary information

Structural insights into a shared mechanism of human STING activation by a potent agonist and an autoimmune disease-associated mutation

## Data Availability

The density maps of HB3089-bound STING and the mutant V147L have been deposited in the Electron Microscopy Bank under accession codes EMD-34245 and EMD-34244, respectively. The corresponding atomic coordinate have been deposited in the Protein Data Bank under accession code 8GT6 and 8GSZ, respectively.

## References

[1] Ishikawa H, Barber GN (2008). STING is an endoplasmic reticulum adaptor that facilitates innate immune signalling. Nature.

[2] Jin L (2008). MPYS, a novel membrane tetraspanner, is associated with major histocompatibility complex class II and mediates transduction of apoptotic signals. Mol. Cell Biol..

[3] Sun W (2009). ERIS, an endoplasmic reticulum IFN stimulator, activates innate immune signaling through dimerization. Proc. Natl. Acad. Sci. USA.

[4] Zhong B (2008). The adaptor protein MITA links virus-sensing receptors to IRF3 transcription factor activation. Immunity.

[5] Sun L, Wu J, Du F, Chen X, Chen ZJ (2013). Cyclic GMP-AMP synthase is a cytosolic DNA sensor that activates the type I interferon pathway. Science.

[6] Woodward JJ, Iavarone AT, Portnoy DA (2010). c-di-AMP secreted by intracellular Listeria monocytogenes activates a host type I interferon response. Science.

[7] Ablasse RA (2013). cGAS produces a 2’-5’-linked cyclic dinucleotide second messenger that activates STING. Nature.

[8] McWhirter SM (2009). A host type I interferon response is induced by cytosolic sensing of the bacterial second messenger cyclic-di-GMP. J. Exp. Med..

[9] Diner EJ (2013). The innate immune DNA sensor cGAS produces a noncanonical cyclic dinucleotide that activates human STING. Cell Rep..

[10] Eaglesham JB, Kranzusch PJ (2020). Conserved strategies for pathogen evasion of cGAS-STING immunity. Curr. Opin. Immunol..

[11] Liu S (2015). Phosphorylation of innate immune adaptor proteins MAVS, STING, and TRIF induces IRF3 activation. Science.

[12] Tanaka Y, Chen ZJ (2012). STING specifies IRF3 phosphorylation by TBK1 in the cytosolic DNA signaling pathway. Sci. Signal.

[13] Jin L (2011). MPYS is required for IFN response factor 3 activation and type I IFN production in the response of cultured phagocytes to bacterial second messengers cyclic-di-AMP and cyclic-di-GMP. J. Immunol..

[14] Yu X (2021). The STING phase-separator suppresses innate immune signalling. Nat. Cell Biol..

[15] Mukai K (2021). Homeostatic regulation of STING by retrograde membrane traffic to the ER. Nat. Commun..

[16] Ahn J, Gutman D, Saijo S, Barber GN (2012). STING manifests self DNA-dependent inflammatory disease. Proc. Natl. Acad. Sci. USA.

[17] Dobbs N (2015). STING activation by translocation from the ER is associated with infection and autoinflammatory disease. Cell Host Microbe.

[18] Jeremiah N (2014). Inherited STING-activating mutation underlies a familial inflammatory syndrome with lupus-like manifestations. J. Clin. Invest..

[19] Wang L, Wang FS, Gershwin ME (2015). Human autoimmune diseases: a comprehensive update. J. Intern. Med..

[20] Saldanha RG (2018). A Mutation outside the dimerization domain causing atypical STING-associated vasculopathy with onset in infancy. Front. Immunol..

[21] Melki I (2017). Disease-associated mutations identify a novel region in human STING necessary for the control of type I interferon signaling. J. Allergy Clin. Immunol..

[22] Liu Y (2014). Activated STING in a vascular and pulmonary syndrome. N. Engl. J. Med..

[23] Ergun SL, Fernandez D, Weiss TM, Li L (2019). STING polymer structure reveals mechanisms for activation,hyperactivation, and Inhibition. Cell.

[24] Fang R (2021). Golgi apparatus-synthesized sulfated glycosaminoglycans mediate polymerization and activation of the cGAMP sensor STING. Immunity.

[25] Shang G, Zhang C, Chen ZJ, Bai XC, Zhang X (2019). Cryo-EM structures of STING reveal its mechanism of activation by cyclic GMP-AMP. Nature.

[26] Zhang C (2019). Structural basis of STING binding with and phosphorylation by TBK1. Nature.

[27] Zhao B (2019). A conserved PLPLRT/SD motif of STING mediates the recruitment and activation of TBK1. Nature.

[28] Shu C, Yi G, Watts T, Kao CC, Li P (2012). Structure of STING bound to cyclic di-GMP reveals the mechanism of cyclic dinucleotide recognition by the immune system. Nat. Struct. Mol. Biol..

[29] Taguchi T, Mukai K (2019). Innate immunity signalling and membrane trafficking. Curr. Opin. Cell Biol..

[30] Ramanjulu JM (2018). Design of amidobenzimidazole STING receptor agonists with systemic activity. Nature.

[31] Ouyang S (2012). Structural analysis of the STING adaptor protein reveals a hydrophobic dimer interface and mode of cyclic di-GMP binding. Immunity.

[32] Gao P (2013). Structure-function analysis of STING activation by c[G(2’,5’)pA(3’,5’)p] and targeting by antiviral DMXAA. Cell.

[33] Zhang X (2013). Cyclic GMP-AMP containing mixed phosphodiester linkages is an endogenous high-affinity ligand for STING. Mol. Cell.

[34] Saitoh T (2009). Atg9a controls dsDNA-driven dynamic translocation of STING and the innate immune response. Proc. Natl. Acad. Sci. USA.

[35] Li T, Chen ZJ (2018). The cGAS-cGAMP-STING pathway connects DNA damage to inflammation, senescence, and cancer. J. Exp. Med..

[36] Chen Q, Sun L, Chen ZJ (2016). Regulation and function of the cGAS-STING pathway of cytosolic DNA sensing. Nat. Immunol..

[37] Ishikawa H, Ma Z, Barber GN (2009). STING regulates intracellular DNA-mediated, type I interferon-dependent innate immunity. Nature.

[38] Munoz J (2015). Stimulator of interferon genes-associated vasculopathy with onset in infancy: a mimic of childhood granulomatosis with polyangiitis. JAMA Dermatol..

[39] Hansen AL (2019). STING palmitoylation as a therapeutic target. Cell. Mol. Immunol..

[40] Lu D (2022). Activation of STING by targeting a pocket in the transmembrane domain. Nature.

[41] Zhao B (2016). Structural basis for concerted recruitment and activation of IRF-3 by innate immune adaptor proteins. Proc. Natl. Acad. Sci. USA.

[42] Deng Z (2020). A defect in COPI-mediated transport of STING causes immune dysregulation in COPA syndrome. J. Exp. Med..

[43] Lepelley A (2020). Mutations in COPA lead to abnormal trafficking of STING to the Golgi and interferon signaling. J. Exp. Med..

[44] Srikanth S (2019). The Ca(2+) sensor STIM1 regulates the type I interferon response by retaining the signaling adaptor STING at the endoplasmic reticulum. Nat. Immunol..

[45] Zheng SQ (2017). MotionCor2: anisotropic correction of beam-induced motion for improved cryo-electron microscopy. Nat. Methods.

[46] Zhang K (2016). Gctf: Real-time CTF determination and correction. J. Struct. Biol..

[47] Zivanov J (2018). New tools for automated high-resolution cryo-EM structure determination in RELION-3. Elife.

[48] Kucukelbir A, Sigworth FJ, Tagare HD (2014). Quantifying the local resolution of cryo-EM density maps. Nat. Methods.

[49] Pettersen EF (2004). UCSF Chimera-a visualization system for exploratory research and analysis. J. Comput. Chem..

[50] Emsley P, Cowtan K (2004). Coot: model-building tools for molecular graphics. Acta Crystallogr. D. Biol. Crystallogr..

[51] Adams PD (2010). PHENIX: a comprehensive Python-based system for macromolecular structure solution. Acta Crystallogr. D. Biol. Crystallogr..

[52] Williams CJ (2018). MolProbity: More and better reference data for improved all-atom structure validation. Protein Sci..

[53] Pettersen EF (2021). UCSF ChimeraX: Structure visualization for researchers, educators, and developers. Protein Sci..

