# Supplementary material for: Structural insights into a shared mechanism of human STING activation by a potent agonist and an autoimmune disease-associated mutation
Source: Cell Discov. 2022 Dec 13;8:133. doi: 10.1038/s41421-022-00481-4 (PMC9747920; doi:10.1038/s41421-022-00481-4)
Supplement: Supplementary file 1 — Structural insights into a shared mechanism of human STING activation by a potent agonist and an autoimmune disease-associated mutation [file 41421_2022_481_MOESM1_ESM.pdf]

Supplementary Information

Chemical synthesis and characterization:

a. Synthesis of compound HB3089

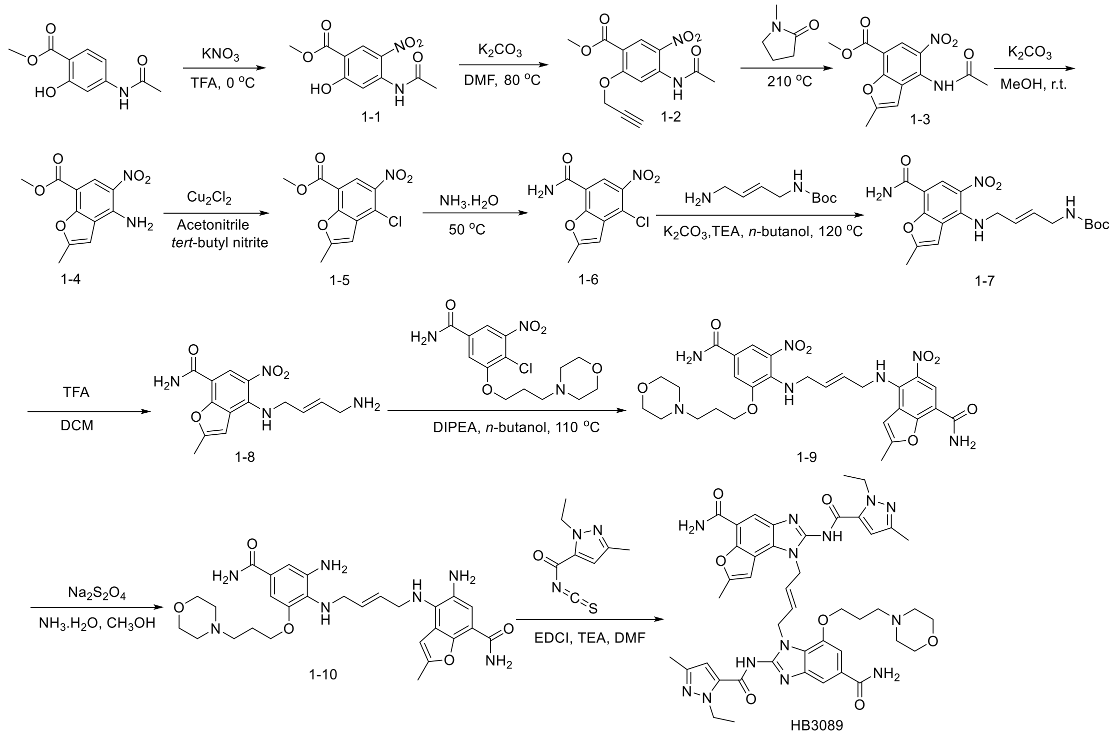

Methyl 4-acetylamino-2-hydroxy-5-nitrobenzoate (1-1): to a mixture of methyl 4-acetamido-2-hydroxybenzoate (5.0 g, 23.9 mmol) in trifluoroacetic acid (35 mL) was added slowly potassium nitrate (3.14 g, 31.1 mmol) at 0 °C. The reaction was warmed to room temperature and stirred for 4 h. The reaction mixture was poured into ice water (800 mL). The resulting yellow solid was filtered and washed with water to provide methyl 4-acetylamino-2-hydroxy-5-nitrobenzoate (6.1 g, 100% yield). ^{1}H NMR (400 MHz, DMSO-*d6*) δ ppm 10.43 (s, 1H), 8.45 (s, 1H), 7.76 (s, 1H), 3.86 (s, 3H), 2.17 (s, 3H). ^{13}C NMR (151 MHz, DMSO-*d6*) δ ppm 169.20, 165.71, 163.61, 138.22, 131.27, 129.46, 110.37, 109.59, 52.50, 24.47. LCMS (m/z): 255.05 $[M + H]^+$.

Methyl 4-acetamido-5-nitro-2-(prop-2-yn-1-yloxy)benzoate (1-2): a mixture of methyl 4-acetylamino-2-hydroxy-5-nitrobenzoate (1 g, 3.93 mmol) and potassium carbonate (1.6 g, 15.74 mmol) in DMF (20 mL) was heated to 80 °C, and then to the mixure was added 3-bromopropyne (0.52 g, 4.33 mmol) dropwise. After addition, the mixture was refluxed at 80 °C for 2 h. The mixture was poured into ice water (200 mL) and stirred for 1 h. The resulting solid was collected by filtration to afford methyl 4-acetamido-5-nitro-2-(prop-2-yn-1-yloxy)benzoate (0.85 g, 74% yield). ^{1}H NMR (400 MHz, DMSO-*d6*) δ ppm 10.84–10.69 (m, 1H), 8.37 (s, 1H), 7.81 (s, 1H), 4.98 (d, *J*=2.4 Hz, 2H), 3.81 (s, 3H), 3.75 (d, *J*=2.3 Hz, 1H), 2.15 (s, 3H). ^{13}C NMR (151 MHz, DMSO-*d6*) δ ppm 169.06, 163.45, 160.12, 137.16, 133.34, 129.11, 115.10, 107.58,

79.72, 77.59, 56.94, 52.34, 24.07. LCMS (m/z): 293.10 $[M + H]^+$.

Methyl 4-acetamido-2-methyl-5-nitrobenzofuran-7-carboxylate (1-3): a solution of methyl 4-acetamido-5-nitro-2-(prop-2-yn-1-yloxy)benzoate (28 g, 95.8 mmol) in 1-methyl-2-pyrrolidinone (200 mL) was stirred at 210 °C for 4 h. The mixture was cooled to room temperature and quenched with ice water. The suspension was extracted with ethyl acetate three times. The organic phases were dried over anhydrous sodium sulfate and evaporate to obtain methyl 4-acetamido-2-methyl-5-nitrobenzofuran-7-carboxylate (14 g, 50% yield) as a yellow solid. ^{1}H NMR (400 MHz, DMSO-d_6) δ ppm 11.17 (s, 1H), 8.22 (s, 1H), 7.11 (d, J=1.3 Hz, 1H), 3.93 (s, 3H), 2.53 (d, J=1.1 Hz, 3H), 2.16 (s, 3H). ^{13}C NMR (151 MHz, DMSO-d_6) δ ppm 168.65, 162.82, 158.64, 153.67, 138.71, 127.79, 126.57, 122.22, 110.26, 102.99, 52.57, 22.98, 13.87. LCMS (m/z): 293.10 $[M + H]^+$.

Methyl 4-amino-2-methyl-5-nitrobenzofuran-7-carboxylate (1-4): to a mixture of methyl 4-acetamido-2-methyl-5-nitrobenzofuran-7-carboxylate (14 g, 47.9 mmol), methanol (180 mL), and potassium carbonate (33 g, 239.5 mmol) was stirred at room temperature overnight. The resulting solid was collected by filtration and washed with water to afford methyl 4-amino-2-methyl-5-nitrobenzofuran-7-carboxylate (9 g, 80% yield) as a yellow solid. ^{1}H NMR (400 MHz, DMSO-d_6) δ ppm 8.50 (s, 1H), 8.32 (s, 2H), 7.15 (s, 1H), 3.86 (s, 3H), 2.46 (d, 3H). ^{13}C NMR (151 MHz, DMSO-d_6) δ ppm 163.18, 155.26, 153.95, 143.45, 125.69, 125.52, 118.47, 103.84, 102.31, 51.92, 13.73. LCMS (m/z): 251.10 $[M + H]^+$.

Methyl 4-chloro-2-methyl-5-nitrobenzofuran-7-carboxylate (1-5): to a mixture of Cu_2Cl_2 (5.34 g, 53.9 mmol) in acetonitrile (50 mL) was added *tert*-butyl nitrite (6.5 mL 53.9 mmol) dropwise at room temperature. The mixture was heated to 50 °C and stirred for 30 min, to the reaction mixture was added slowly a solution of methyl 4-amino-2-methyl-5-nitrobenzofuran-7-carboxylate (7.5 g, 29.9 mmol) in acetonitrile (40 mL). After addition, the mixture was stirred for 5 h, and the mixture was cooled to room temperature and filtered. The filtrate was extracted with ethyl acetate three times, the organic phases was washed with water and brine, dried over sodium sulfate and concentrated in vacuo to obtain methyl 4-chloro-2-methyl-5-nitrobenzofuran-7-carboxylate (6.8 g, 84% yield) as a brown solid. ^{1}H NMR (400 MHz, $CDCl_3$) δ ppm 8.79 (s, 1H), 6.46 (s, 1H), 3.96 (s, 3H), 2.55 (s, 3H). LCMS (m/z): 270.00 $[M + H]^+$.

4-Chloro-2-methyl-5-nitrobenzofuran-7-carboxamide (1-6): a mixture of methyl 4-chloro-2-methyl-5-nitrobenzofuran-7-carboxylate (6.8 g, 25.2 mmol) in $NH_3{\cdot}H_2O$ (80 mL) was stirred at 50 °C overnight. After cooling to room temperature, the resulting mixture was filtered, and the filtering cake was dried to give 4-chloro-2-methyl-5-nitrobenzofuran-7-carboxamide (5 g, 78% yield). ^{1}H NMR (400 MHz, DMSO-d_6) δ ppm 8.26 (s, 1H), 8.07 (s, 1H), 7.91 (s, 1H), 6.91 (d, J=1.4 Hz, 1H), 2.56 (d, J=1.2 Hz, 3H). ^{13}C NMR (151 MHz, DMSO-d_6) δ ppm 162.72, 161.00, 151.47, 142.36, 130.82, 121.20, 119.93, 117.81, 102.52, 13.86. LCMS (m/z): 255.00 $[M + H]^+$.

***Tert*-butyl-(*E*)-(4-((7-carbamoyl-2-methyl-5-nitrobenzofuran-4-yl)amino)but-2-en-1-yl)carbamate (1-7):** to a sealed tube was added 4-chloro-2-methyl-5-nitrobenzofuran-7-carboxamide (prepared according to literature procedures[1]) (0.32 g, 1.28 mmol), 1-*N*-Boc-2-trans-butene-1, 4-diamine (prepared according to literature procedures[1]) (0.38g, 2.04mmol), potassium carbonate (0.35g, 2.56mmol), triethylamine (0.39 g, 3.87 mmol), and *n*-butanol (6.0 mL). The resulting mixture was stirred at 120 °C for 8 h. The mixture was diluted with water, and the resulting solids were filtered to afford *tert*-butyl-(*E*)-(4-((7-carbamoyl-2-methyl-5-nitrobenzofuran-4-yl)amino)but-2-en-1-yl)carbamate. (0.31 g, 78.1 % yield) as an orange solid. ^{1}H NMR (400 MHz, DMSO-d_6) δ ppm 9.05 (t, *J*=6.0 Hz, 1H), 8.51 (s, 1H), 7.70 (s, 1H), 7.53 (s, 1H), 7.01 (d, *J*=8.0 Hz, 2H), 5.83–5.62 (m, 2H), 4.34 (t, *J*=5.3 Hz, 2H), 3.55 (d, *J*=6.1 Hz, 2H), 2.47–2.45 (m, 3H), 1.35 (s, 9H). ^{13}C NMR (151 MHz, DMSO-d_6) δ ppm 163.42, 155.41, 154.82, 154.46, 143.05, 130.00, 126.99, 126.14, 124.86, 115.44, 108.30, 105.04, 77.62, 45.60, 41.09, 28.21, 28.21, 28.21, 13.43. LCMS (m/z): 427.50 [M + Na]$^{+}$.

(*E*)-4-((4-Aminobut-2-en-1-yl)amino)-2-methyl-5-nitrobenzofuran-7-carboxamide (1-8): to a solution of compound **1-7** (5.40 g, 13.35 mmol) in dry DCM (10 mL) was added trifluoracetic acid (TFA) (15.20g, 133.31 mmol) dropwise and stirred at room temperature for 4 h. The resulting suspension was filtered to afford the light orange solid (*E*)-4-((4-aminobut-2-en-1-yl)amino)-2-methyl-5-nitrobenzofuran-7-carboxamide (3.55g, 87.3 % yield). ^{1}H NMR (400 MHz, DMSO-d_6) δ ppm 9.07 (t, *J*=6.0 Hz, 1H), 8.49 (s, 1H), 7.71 (s, 1H), 7.54 (s, 1H), 7.02 (s, 1H), 6.12–5.94 (m, 1H), 5.89–5.64 (m, 1H), 4.38 (d, *J*=5.6 Hz, 2H), 3.40 (d, *J*=6.3 Hz, 2H), 2.46 (s, 3H). ^{13}C NMR (151 MHz, DMSO-d_6) δ ppm 163.43, 154.83, 154.71, 143.04, 131.36, 127.09, 124.97, 124.78, 115.49, 108.42, 105.05, 45.49, 40.06, 13.48. LCMS (m/z): 305.10 [M + H]$^{+}$.

(*E*)-4-((4-((4-Carbamoyl-2-(3-morpholinopropoxy)-6-nitrophenyl)amino)but-2-en-1-yl)amino)-2-methyl-5-nitrobenzofuran-7-carboxamide (1-9): to a sealed tube was added compound **3** (0.20 g，0.58 mmol) , 4-chloro-3-(3-morpholinopropoxy)-5-

nitrobenzamide (0.26 g，0.76 mmol), and DIPEA (1 mL, 0.66 mmol) , and n-butanol (6.0 mL). The reaction mixture was stirred at 110 °C for 10 h. After cooling to room temperature, the reaction mixture was diluted with hexanes, and the solids were collected by filtration to give (*E*)-4-((4-((4-carbamoyl-2-(3-morpholinopropoxy)-6-nitrophenyl)amino) but-2-en-1-yl)amino)-2-methyl-5-nitrobenzofuran-7-carboxamide (0.22 g, 54.2 % yield) as a red solid. ^{1}H NMR (400 MHz, DMSO-d_6) δ ppm 9.09 (s, 1H), 8.50 (s, 1H), 8.16 (s, 1H), 8.08 (s, 1H), 7.84 (s, 1H), 7.71 (s, 1H), 7.52–7.46 (m, 2H), 7.33 (s, 1H), 6.87 (s, 1H), 5.77 (s, 2H), 4.31 (s, 2H), 4.17 (s, 2H), 3.94 (s, 2H), 3.51 (d, *J*=6.6 Hz, 4H), 2.39 (s, 3H), 2.27 (s, 6H), 1.86–1.68 (m, 2H). LCMS (m/z): 612.35 $[M + H]^+$.

(*E*)-5-Amino-4-((4-((2-amino-4-carbamoyl-6-(3-morpholinopropoxy)phenyl)amino)but-2-en-1-yl)amino)-2-methylbenzofuran-7-carboxamide (1-10): to a solution of compound **1-9** (0.19 g, 0.31 mmol) in MeOH (15.0 mL) was added sodium dithionite (1.0 g, 5.74 mmol) and $NH_3.H_2O$ (1.0 mL, 31.10 mmol), and the resulting mixture was stirred at room temperature for 2 h. The reaction mixture was filtered, and the organic phase was concentrated in vacuo. The residue was purified by silica gel chromatography (MeOH: DCM=1:15) to afford (*E*)-5-amino-4-((4-((2-amino-4-carbamoyl-6-(3-morpholinopropoxy)phenyl)amino)but-2-en-1-yl)amino)-2-methylbenzofuran-7-carboxamide (0.082 g, 48.1 % yield) as a yellow solid. ^{1}H NMR (400 MHz, Methanol-d_4) δ ppm 7.32 (s, 1H), 6.92 (d, *J*=1.9 Hz, 1H), 6.77 (d, *J*=1.9 Hz, 1H), 6.44 (d, *J*=1.3 Hz, 1H), 5.82–5.75 (m, 2H), 4.11–4.00 (m, 2H), 3.81 (t, *J*=6.0 Hz, 2H), 3.73 (t, *J*=4.7 Hz, 4H), 3.68–3.67 (m, 2H), 2.69 (s, 6H), 2.38 (d, *J*=1.1 Hz, 3H), 1.96–1.83 (m, 2H). ^{13}C NMR (151 MHz, Methanol-d_4) δ ppm 172.44, 169.29, 154.31, 152.42, 150.99, 142.07, 136.78, 131.15, 130.70, 128.67, 128.58, 128.42, 116.77, 116.31, 110.31, 105.52, 103.20, 102.66, 66.66, 65.85, 56.20, 55.20, 54.81, 53.68, 48.43, 47.15, 25.60, 13.84. LCMS (m/z): 552.40 $[M + H]^+$.

(*E*)-1-(4-(5-Carbamoyl-2-(1-ethyl-3-methyl-1H-pyrazole-5-carboxamido)-7-(3-morpholinopropoxy)-1H-benzo[d]imidazol-1-yl)but-2-en-1-yl)-2-(1-ethyl-3-methyl-1H-pyrazole-5-carboxamido)-7-methyl-1H-benzofuro[4,5-d]imidazole-5-carboxamide (HB3089): to a solution of compound **1-10** (0.065 g, 0.15 mmol) in DMF

(4.0 mL) was added 1-ethyl-3-methyl-1H-pyrazole-5-carbonyl isothiocyanate (prepared according to literature procedures[1]) in 1, 4-dioxane (1.5 mL, 0.45mmol) at 0 °C. The mixture was stirred at 0 °C for 35 min, then EDCI (0.085 g, 0.45 mmol) and triethylamine (0.09 g, 0.89 mmol) was added. The mixture was warmed up to room temperature and stirred overnight for 12 h. The reaction mixture was diluted with water, and the resulting solids were filtered and purified by silica gel chromatography (MeOH: DCM=1:15) to afford the title compound HB3089 (0.056 g, mmol, 54.4% yield) as a white solid in 99.1% purity. ^{1}H NMR (600 MHz, DMSO-*d*$_6$) δ ppm 12.88 (s, 1H), 12.82 (s, 1H), 7.95 (s, 1H), 7.90 (s, 1H), 7.79 (s, 1H), 7.63 (s, 1H), 7.50 (s, 1H), 7.32 (s, 1H), 7.11 (s, 1H), 6.58 (d, *J*=4.6 Hz, 2H), 6.54 (s, 1H), 5.82 (d, *J*=4.5 Hz, 2H), 5.00–4.93 (m, 2H), 4.90–4.82 (m, 2H), 4.55 (t, *J*=7.1 Hz, 4H), 3.64 (t, *J*=6.2 Hz, 2H), 3.43 (t, *J*=4.6 Hz, 4H), 2.23 (s, 3H), 2.13 (d, *J*=2.8 Hz, 6H), 2.11 (s, 6H), 1.40 (q, *J*=7.2, 6.7 Hz, 2H), 1.30 (t, *J*=7.2 Hz, 6H). ^{13}C NMR (151 MHz, DMSO-*d*$_6$) δ ppm 167.62, 166.90, 166.70, 164.78, 164.78, 156.10, 152.01, 152.01, 148.89, 145.08, 145.08, 144.08, 139.88, 139.77, 130.05, 129.92, 128.28, 127.24, 123.23, 119.45, 113.30, 112.91, 109.34, 109.34, 108.65, 105.12, 105.12, 99.83, 66.66, 66.03, 66.03, 54.75, 53.09, 53.09, 45.70, 45.66, 44.34, 43.30, 25.41, 16.19, 16.19, 13.53, 13.18, 13.18. LCMS (m/z): 874.60 $[M + H]^+$. HRMS (ESI/Q-TOF) m/z: $[M+H]^+$ Calcd for $C_{44}H_{51}N_{13}O_7$ 874.4107; Found 874.4107.

b. Characterization of compound HB3089

Compound 1-1 ^{1}H NMR:

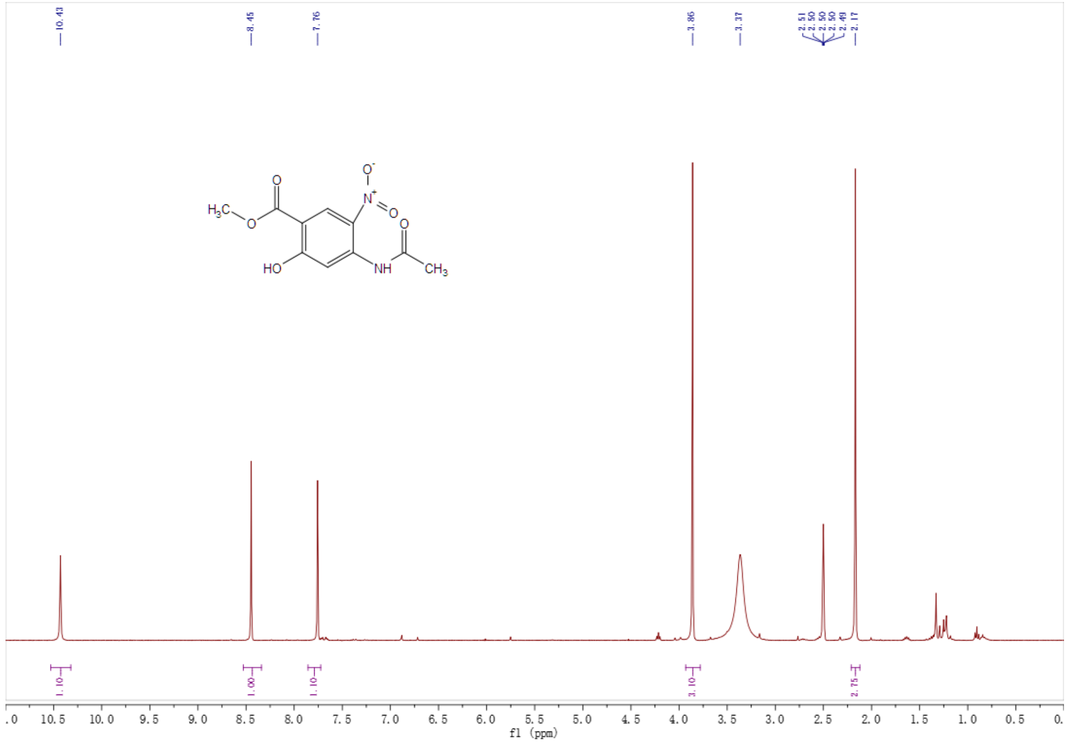

Compound 1-1 ^{13}C NMR:

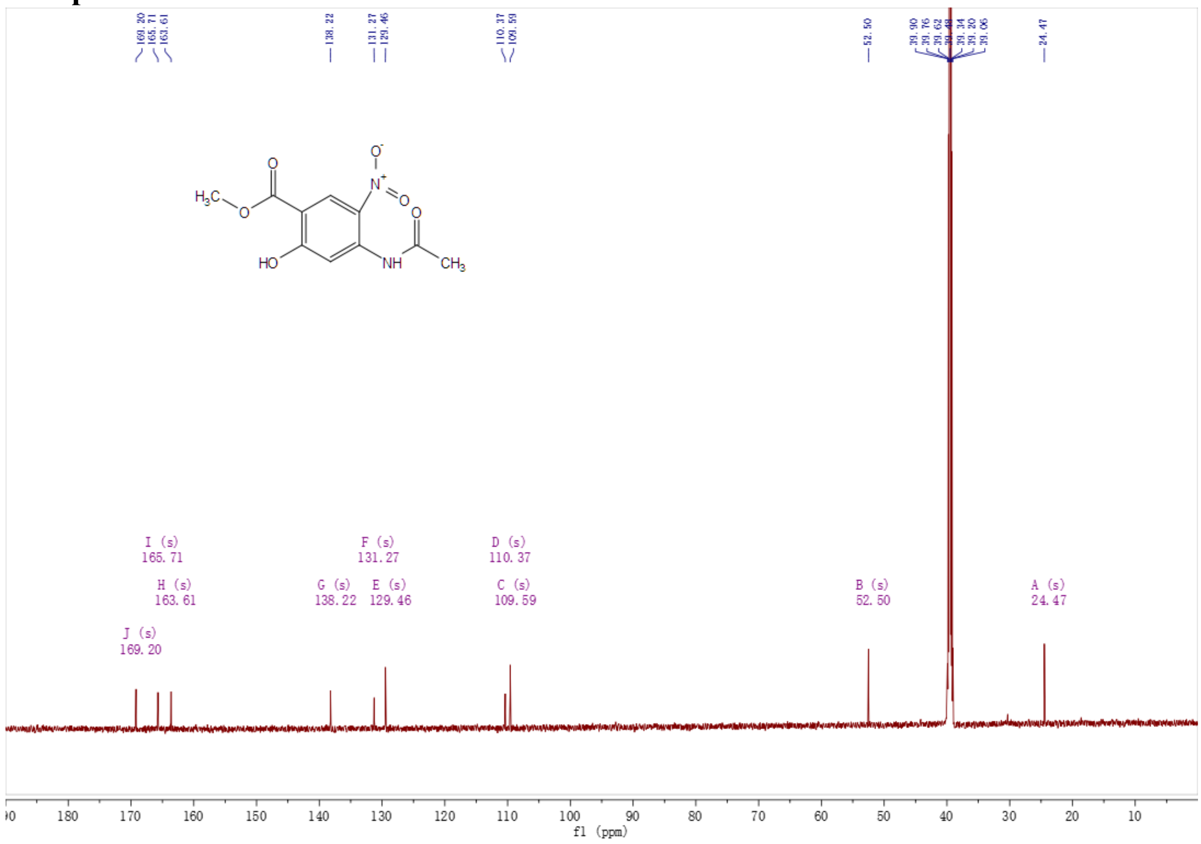

Compound 1-2 ^{1}H NMR:

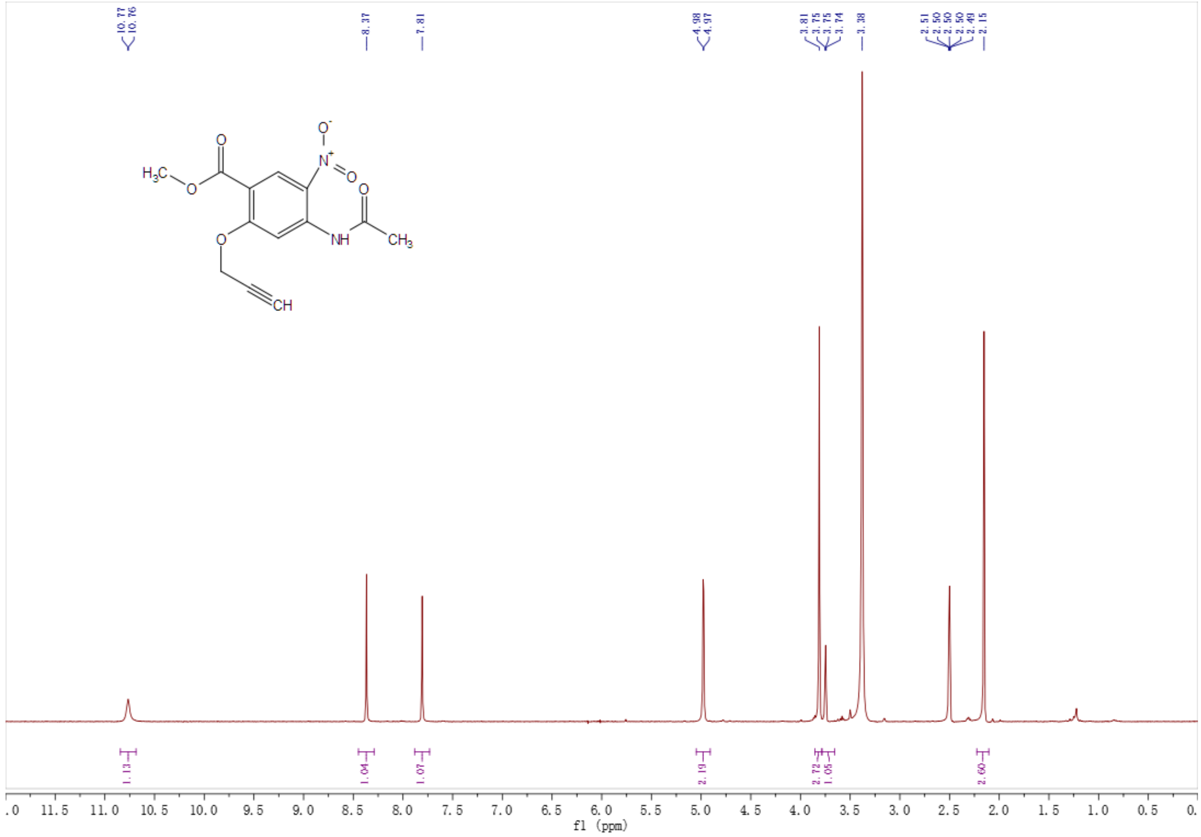

Compound 1-2 ^{13}C NMR:

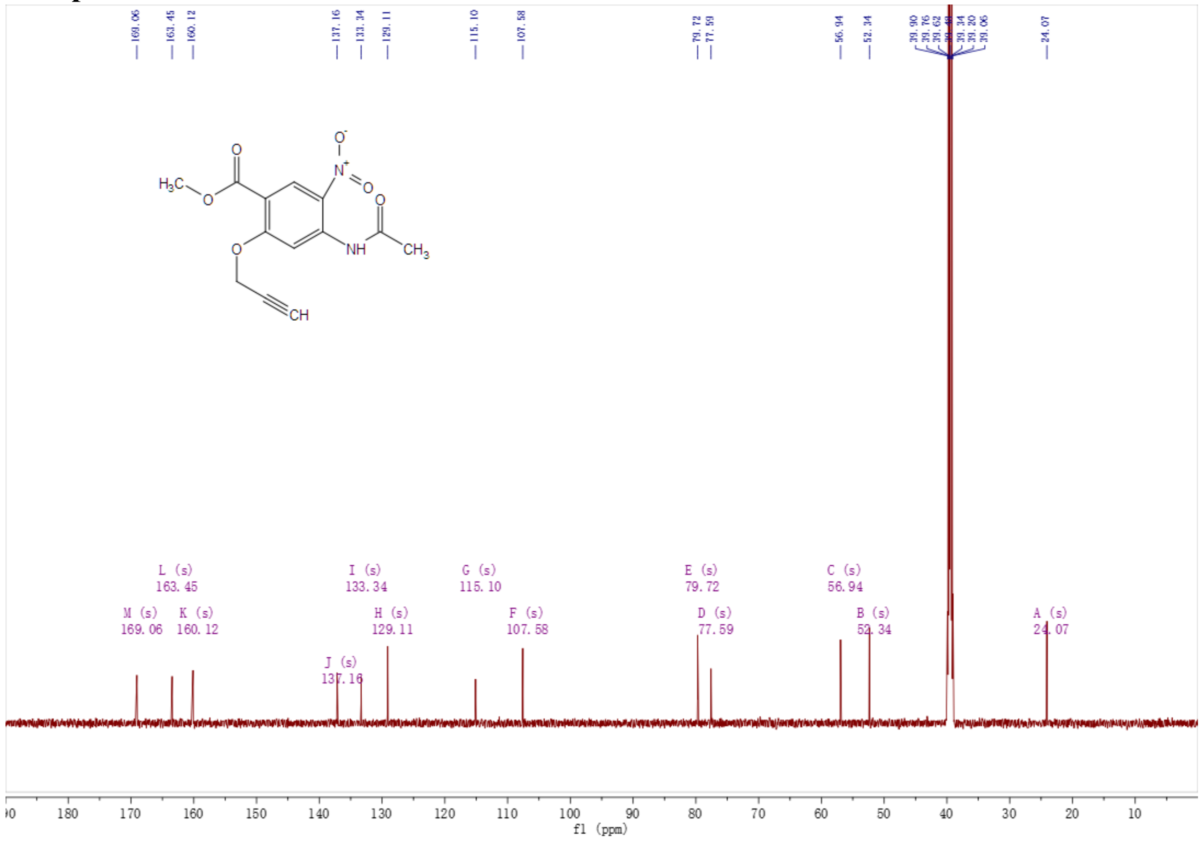

Compound 1-3 ^{1}H NMR:

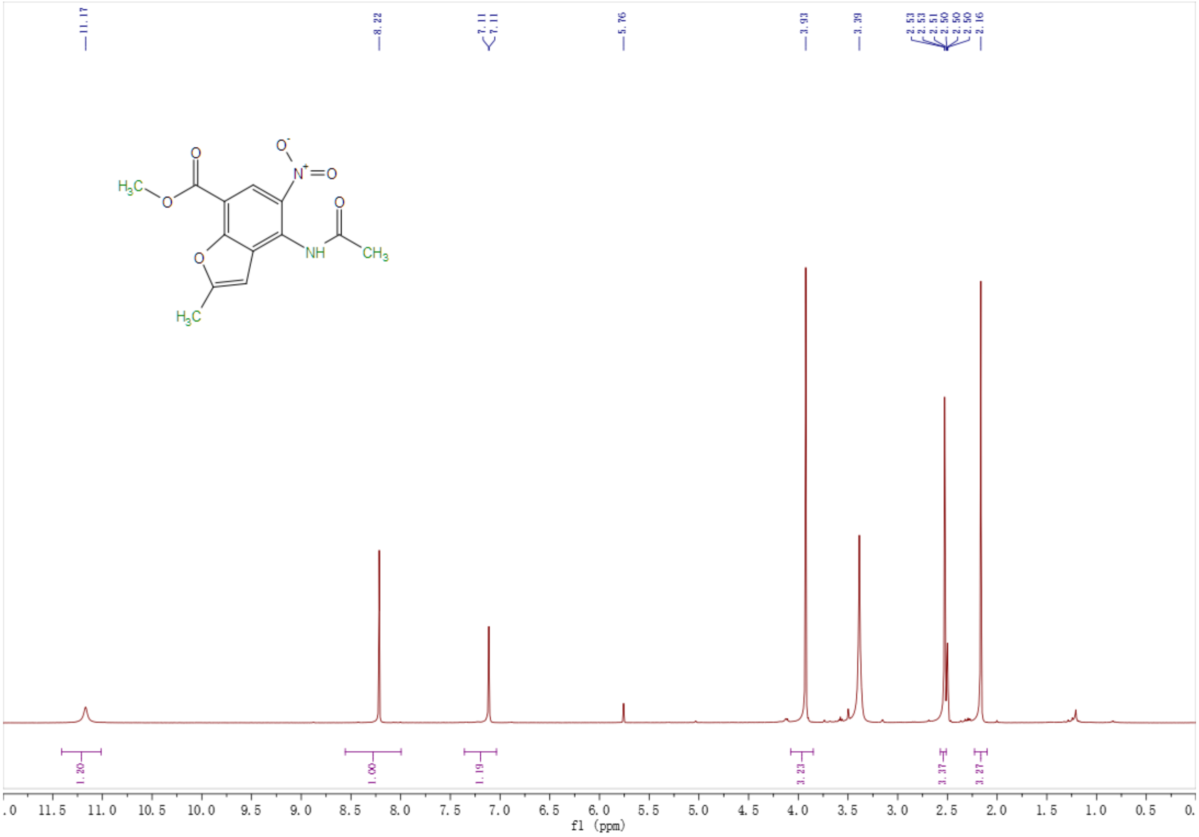

Compound 1-3 ^{13}C NMR:

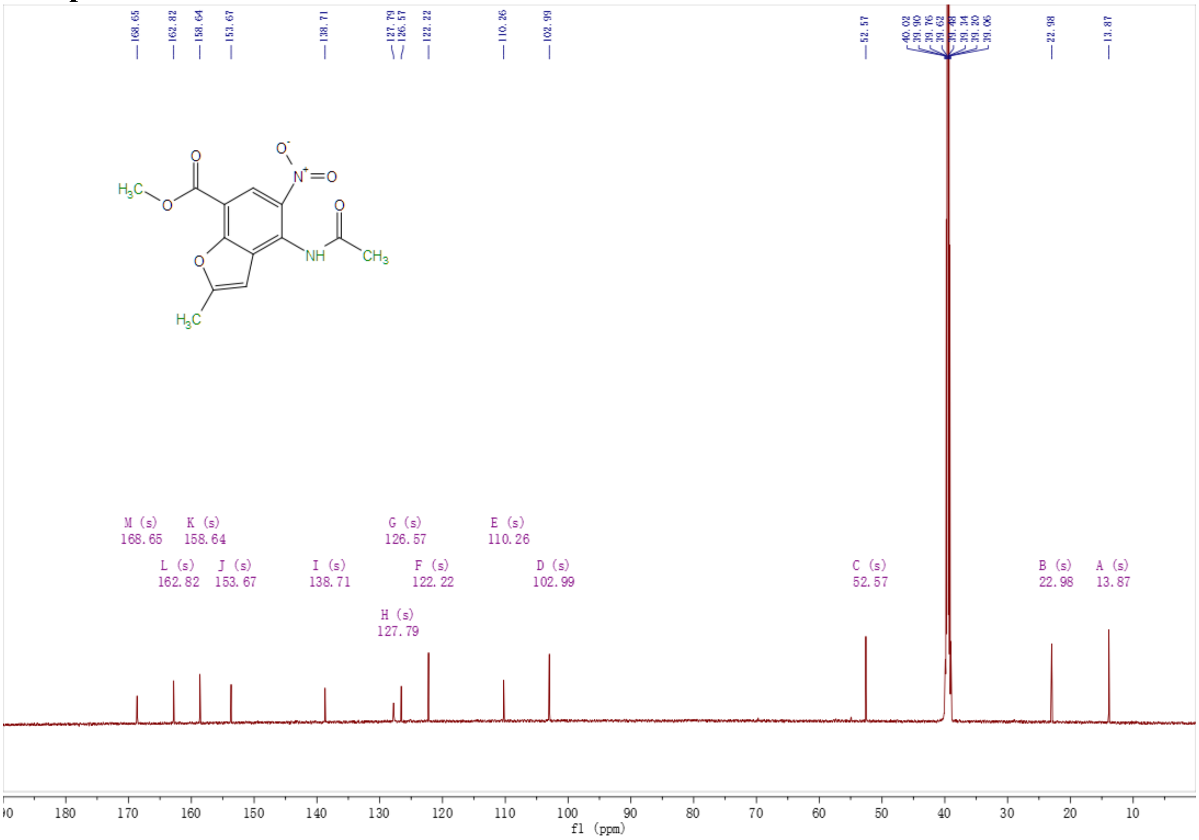

Compound 1-4 ^{1}H NMR:

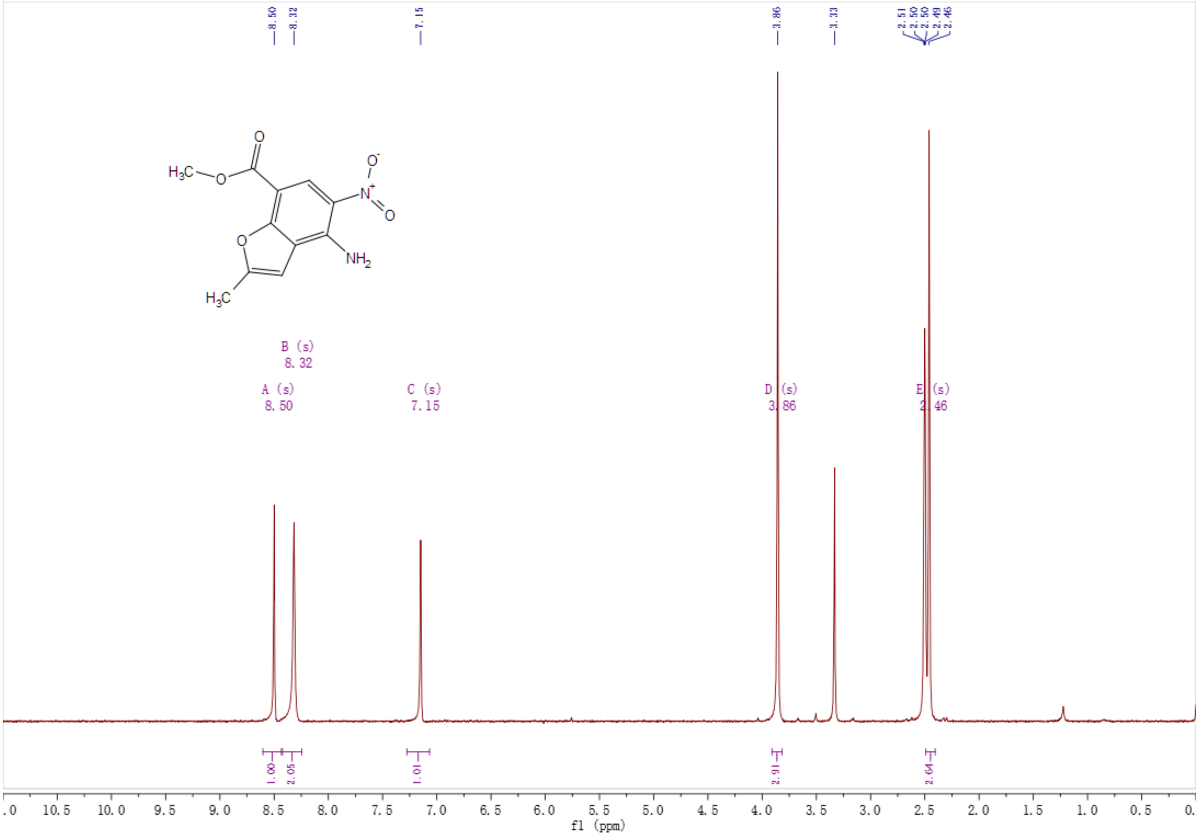

Compound 1-4 ^{13}C NMR:

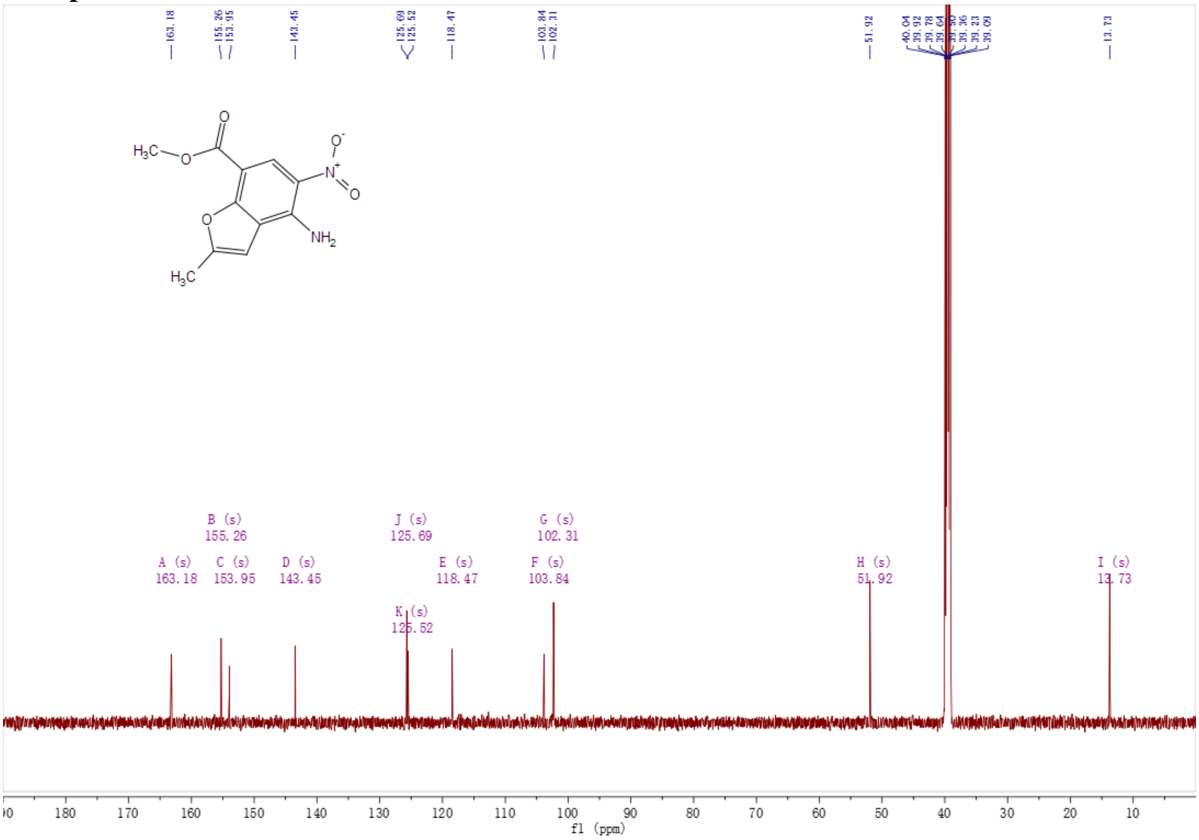

Compound 1-5 ^{1}H NMR:

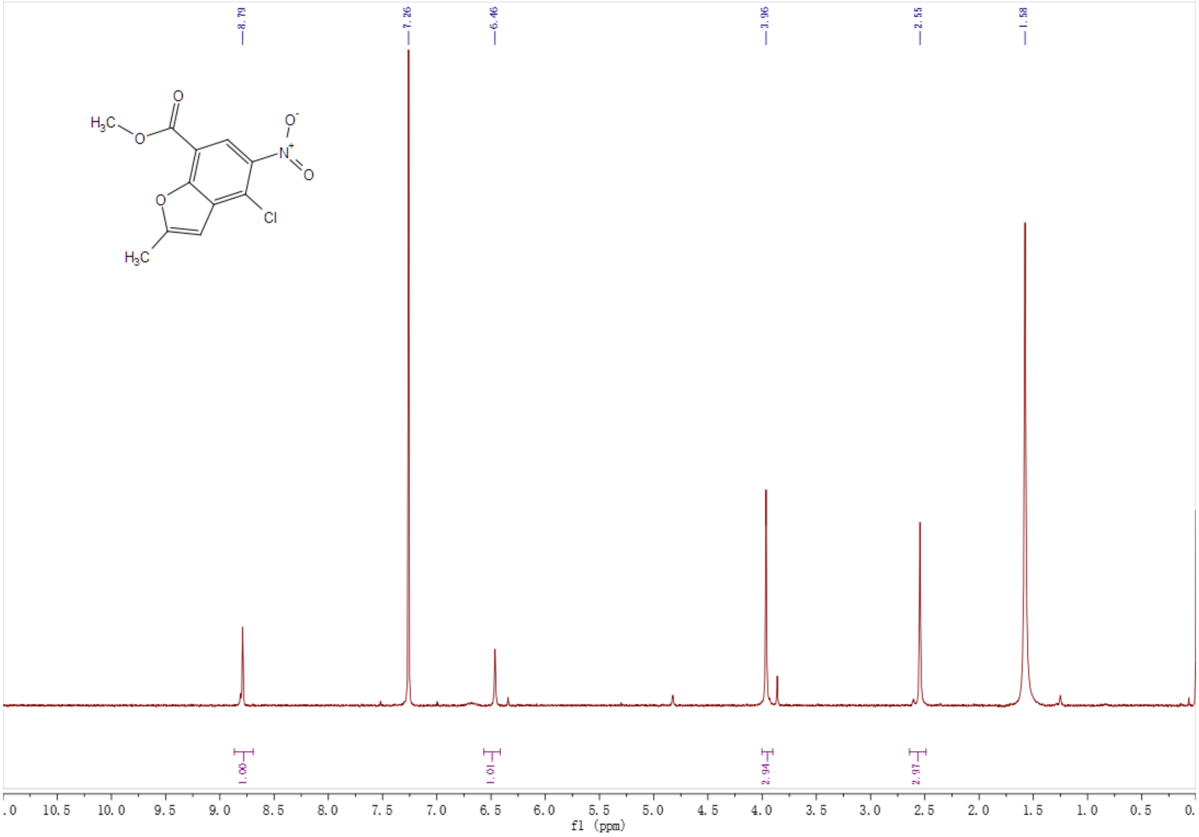

Compound 1-6 ¹H NMR:

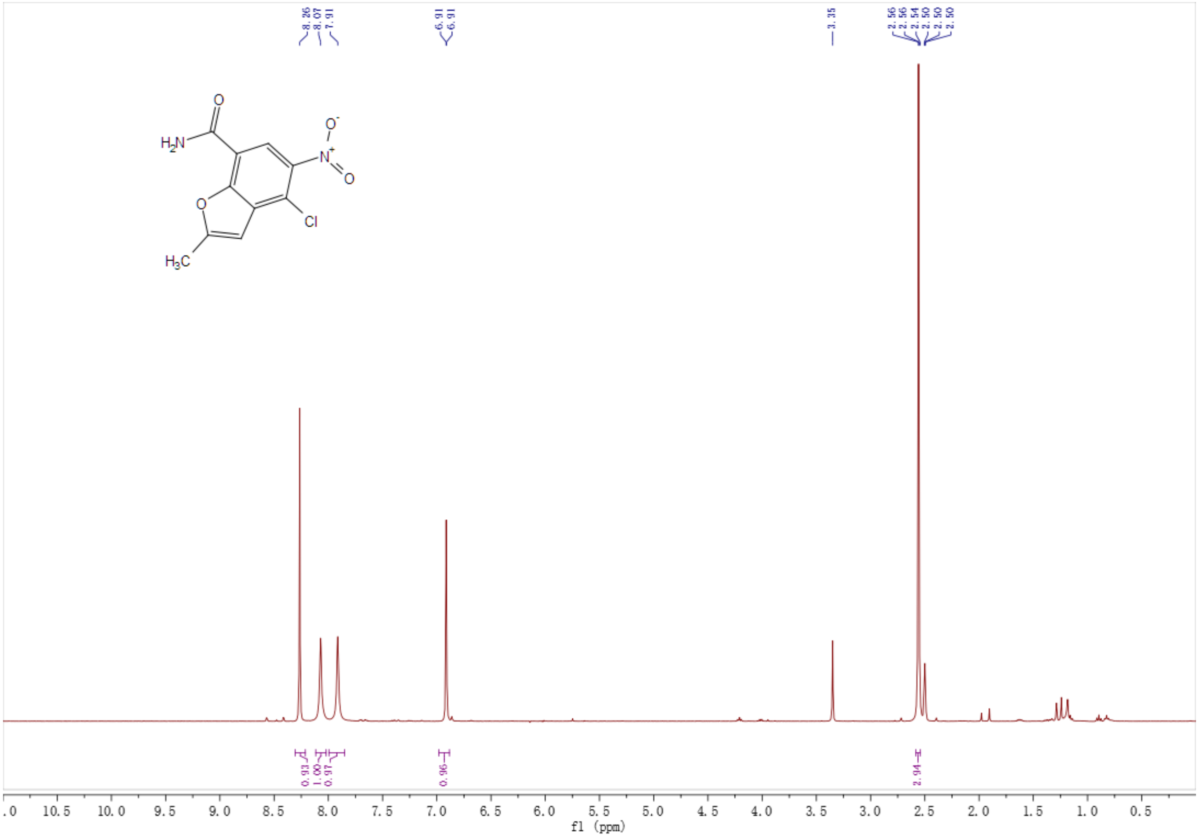

Compound 1-6 ¹³C NMR:

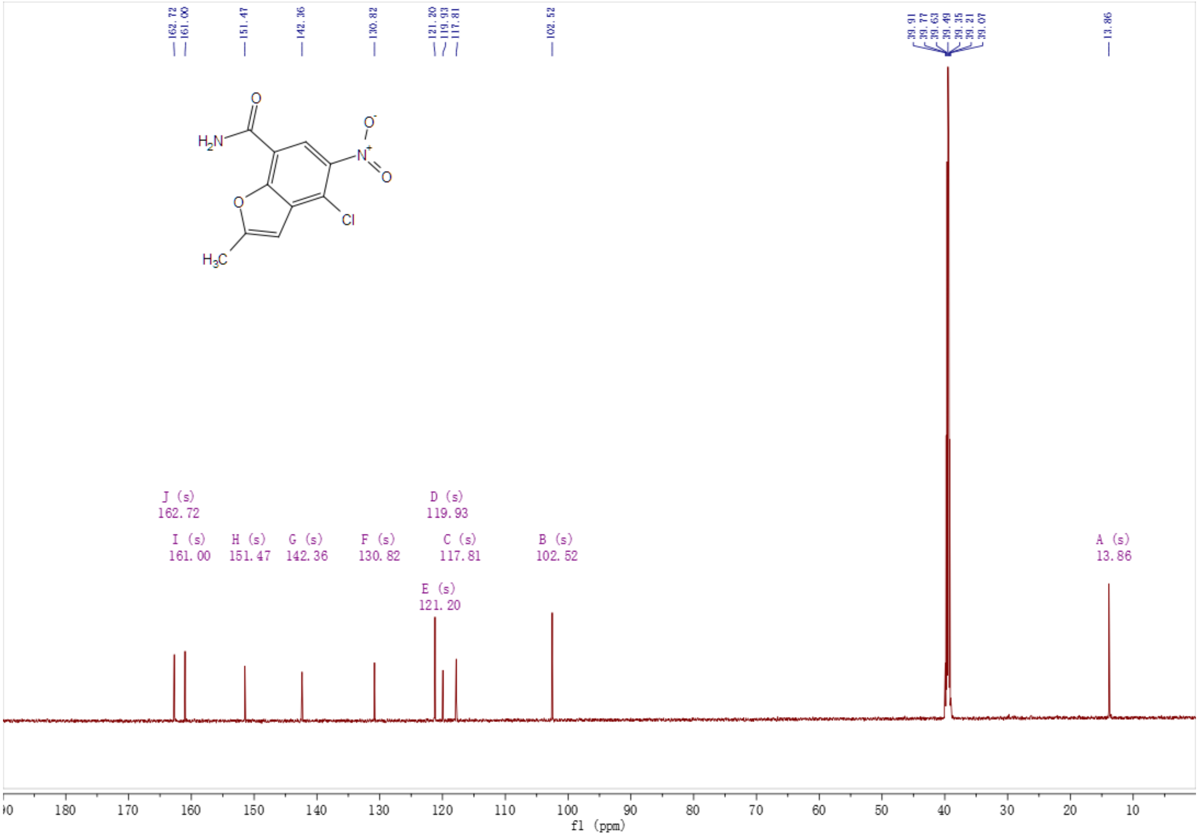

Compound 1-7 - ^{1}H NMR:

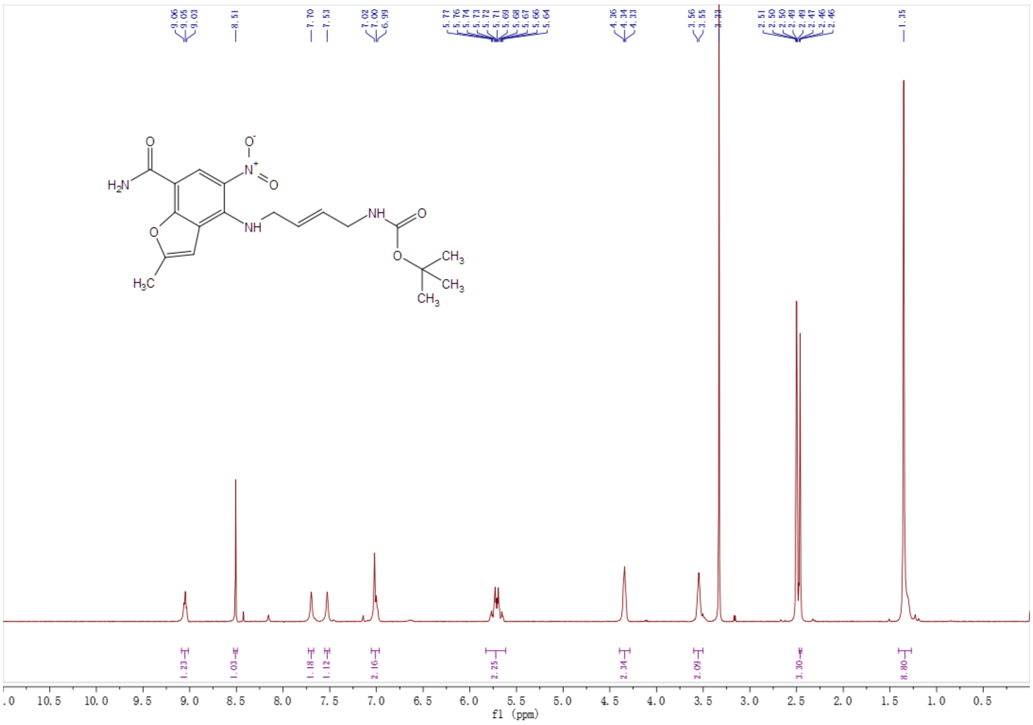

Compound 1-7 – ^{13}C NMR:

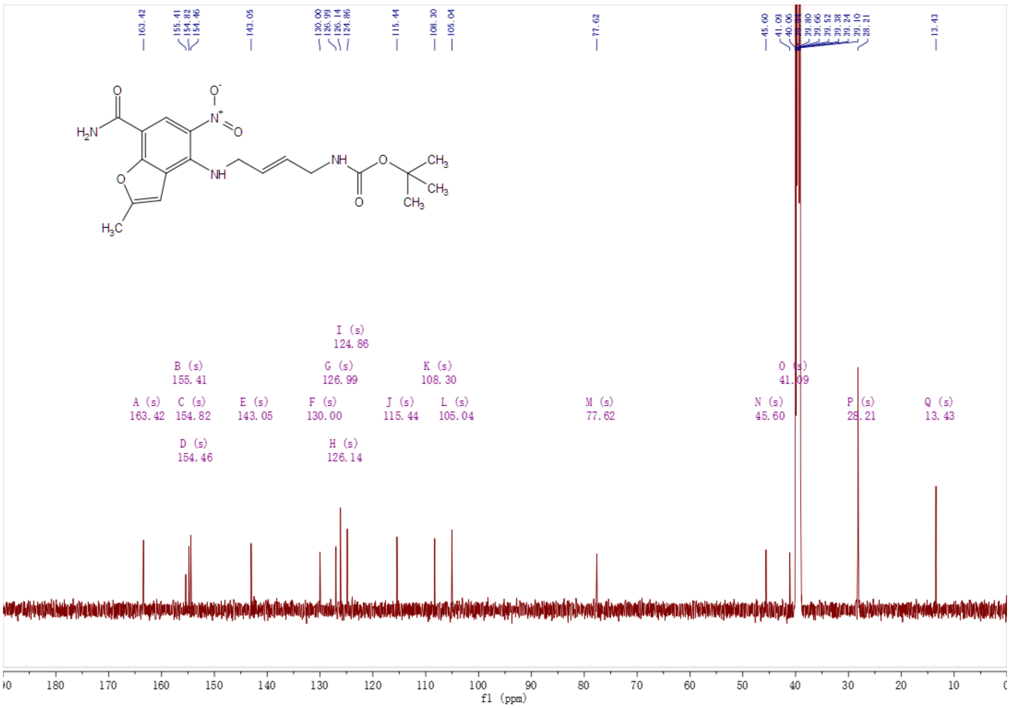

Compound 1-7 – HRMS:

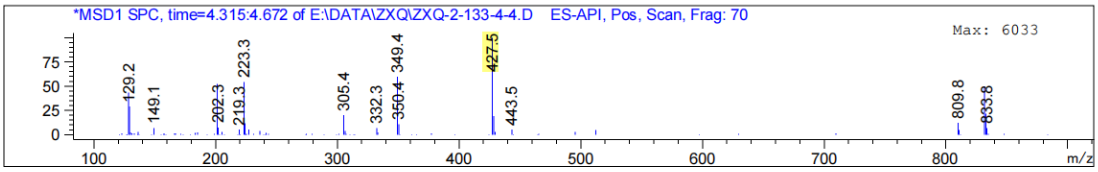

Compound 1-8 - ^{1}H NMR:

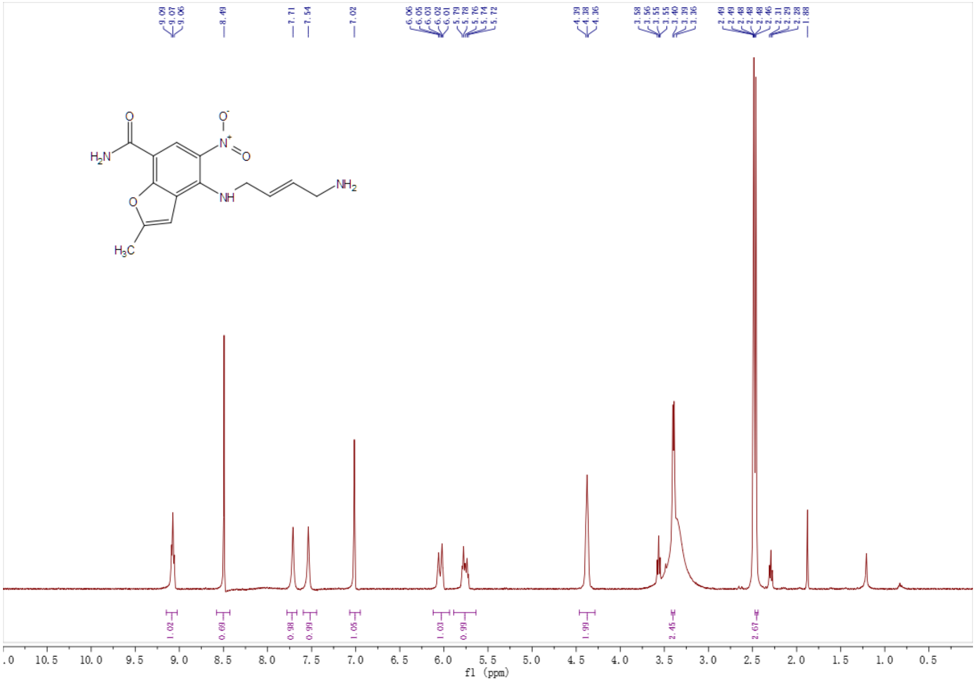

Compound 1-8– ^{13}C NMR:

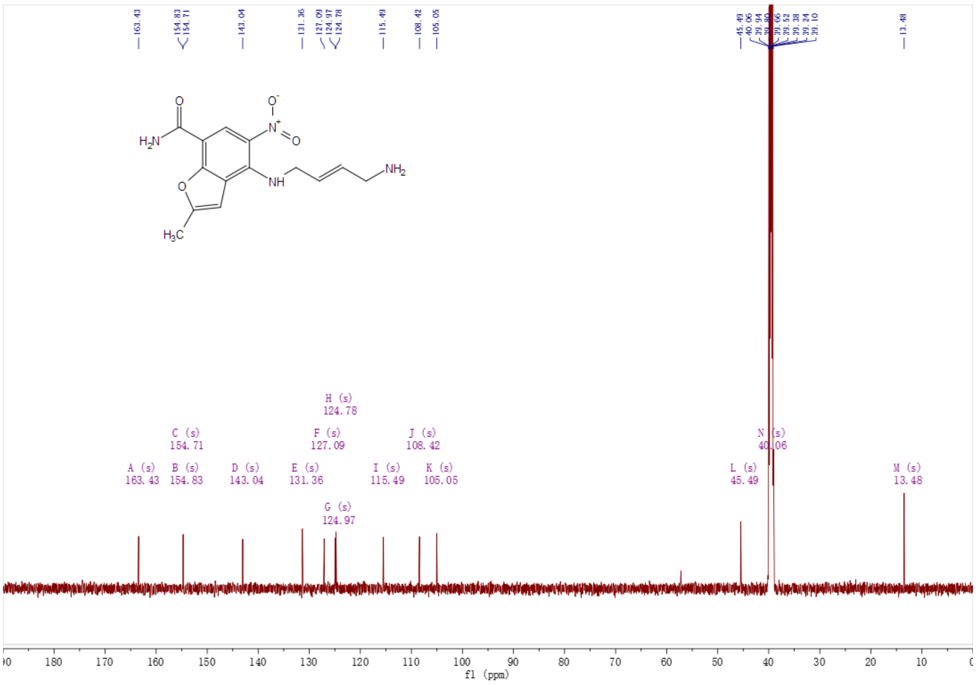

Compound 1-8 – HRMS:

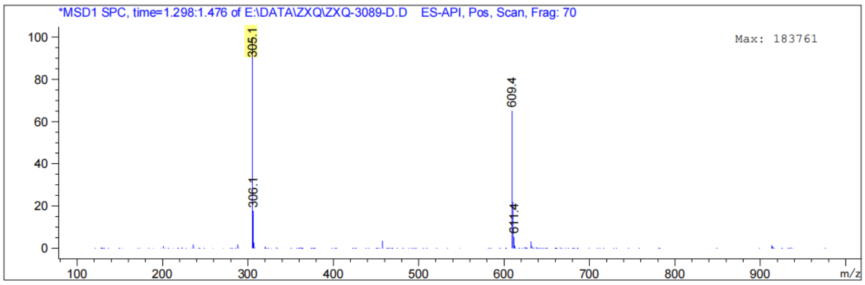

Compound 1-9 - ^{1}H NMR:

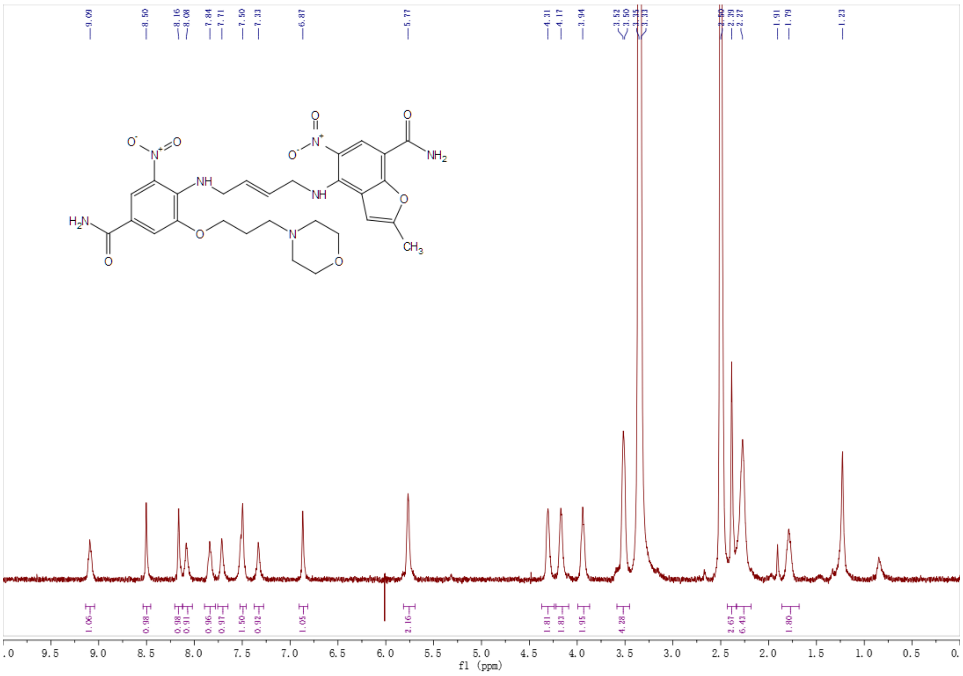

Compound 1-9– HRMS:

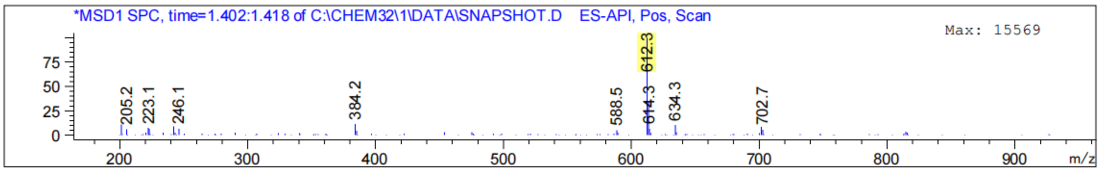

Compound 1-10 - ^{1}H NMR:

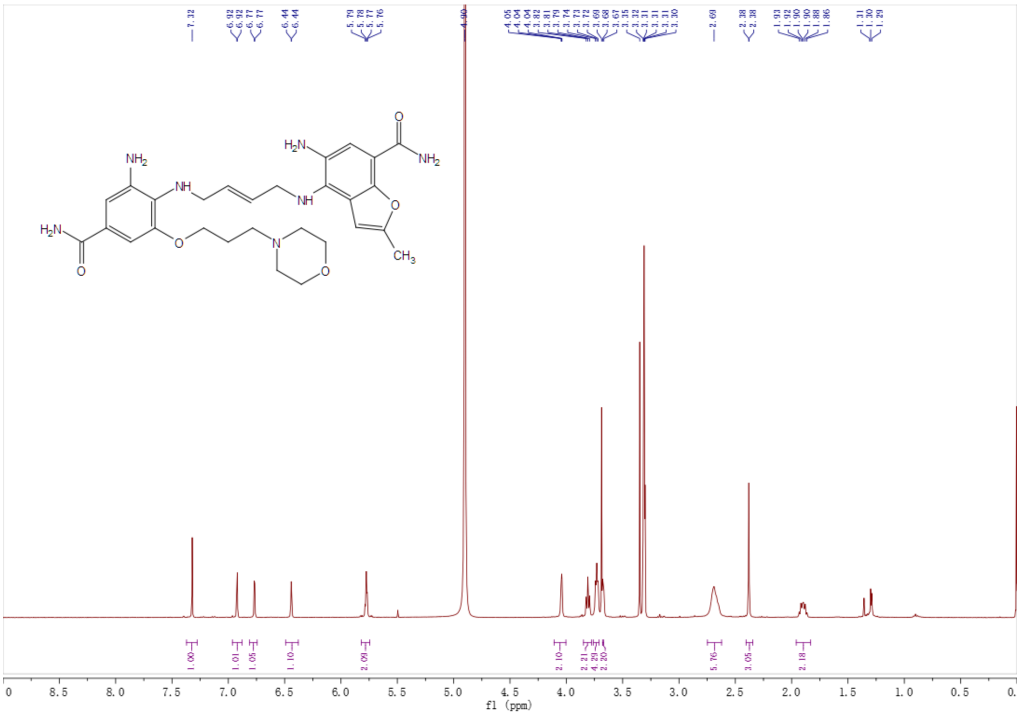

Compound 1-10 – ^{13}C NMR:

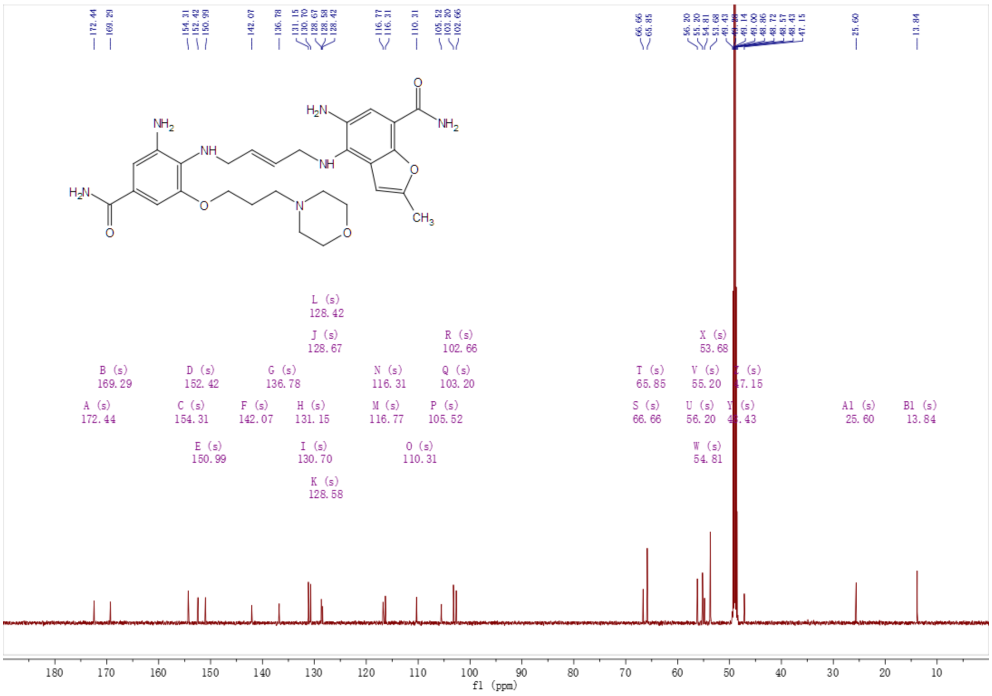

Compound 1-10 – HRMS:

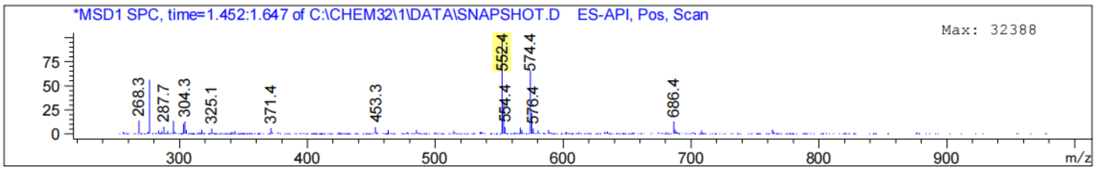

Compound HB3089 - ^{1}H NMR:

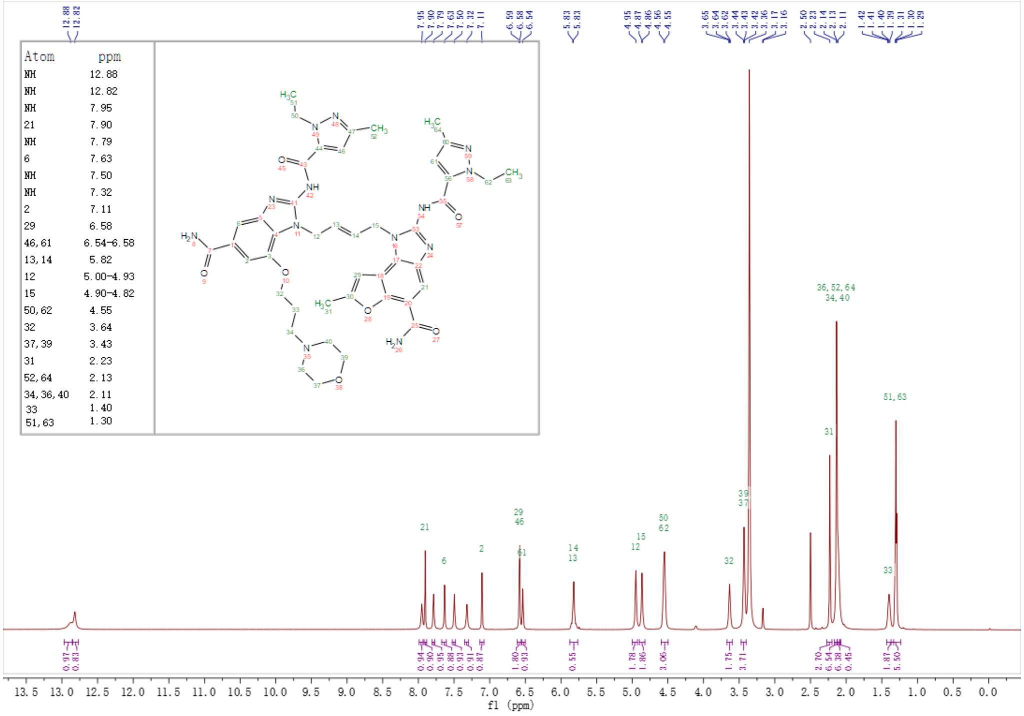

Compound HB3089 – ^{13}C NMR:

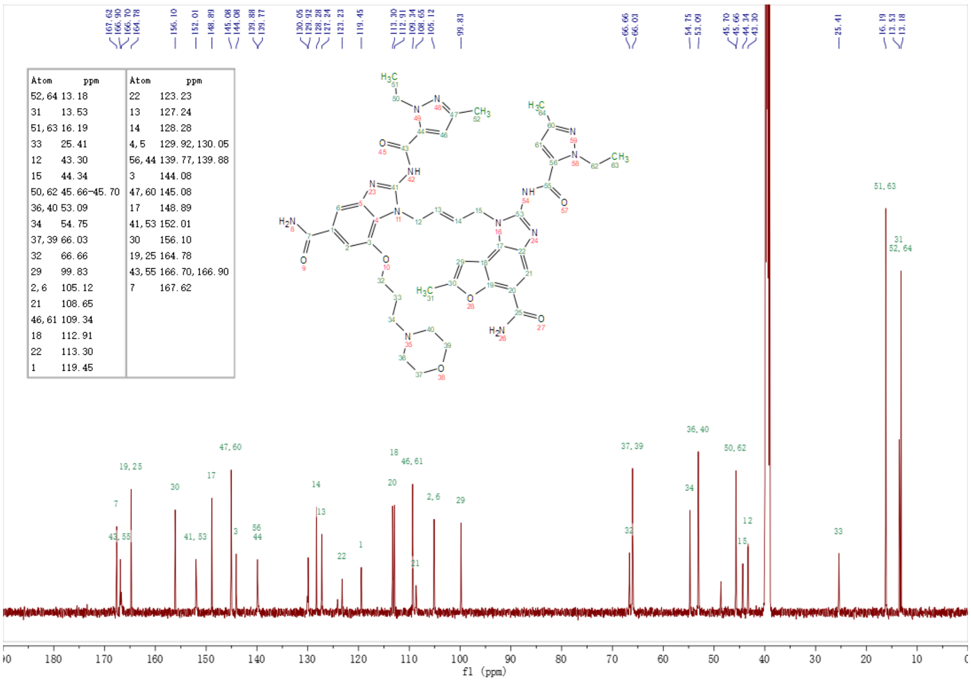

Compound HB3089 DEPT 135:

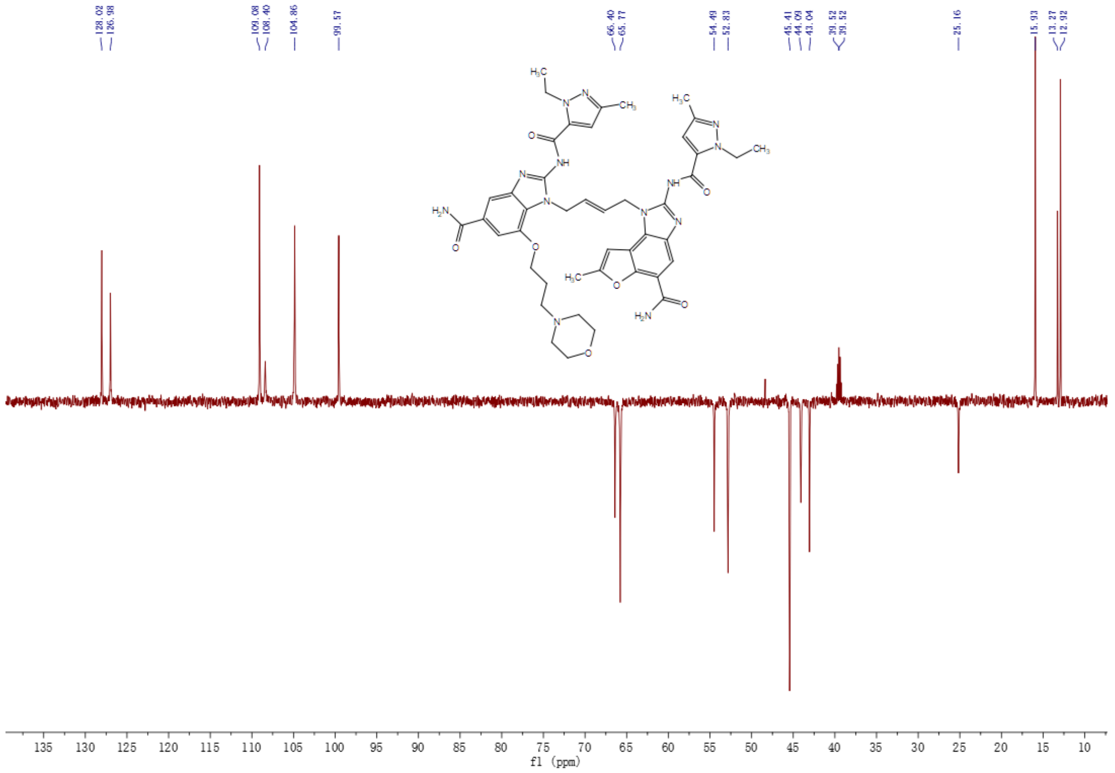

Compound HB3089 COSY:

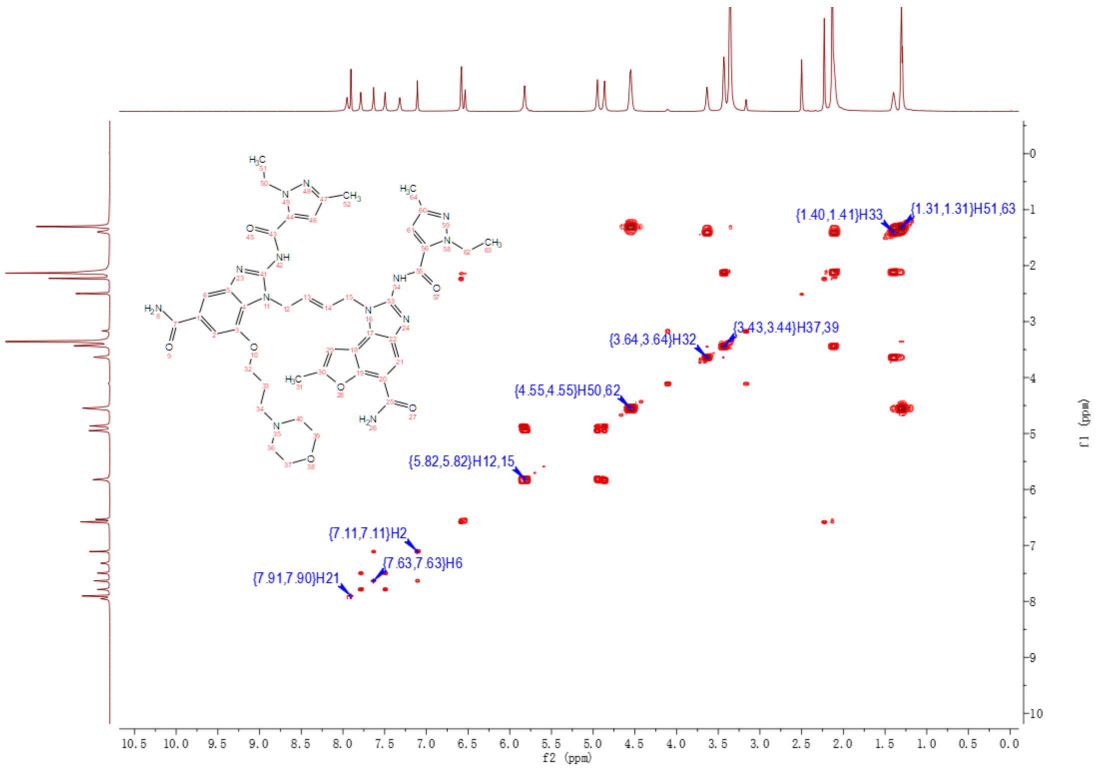

Compound HB3089 HSQC:

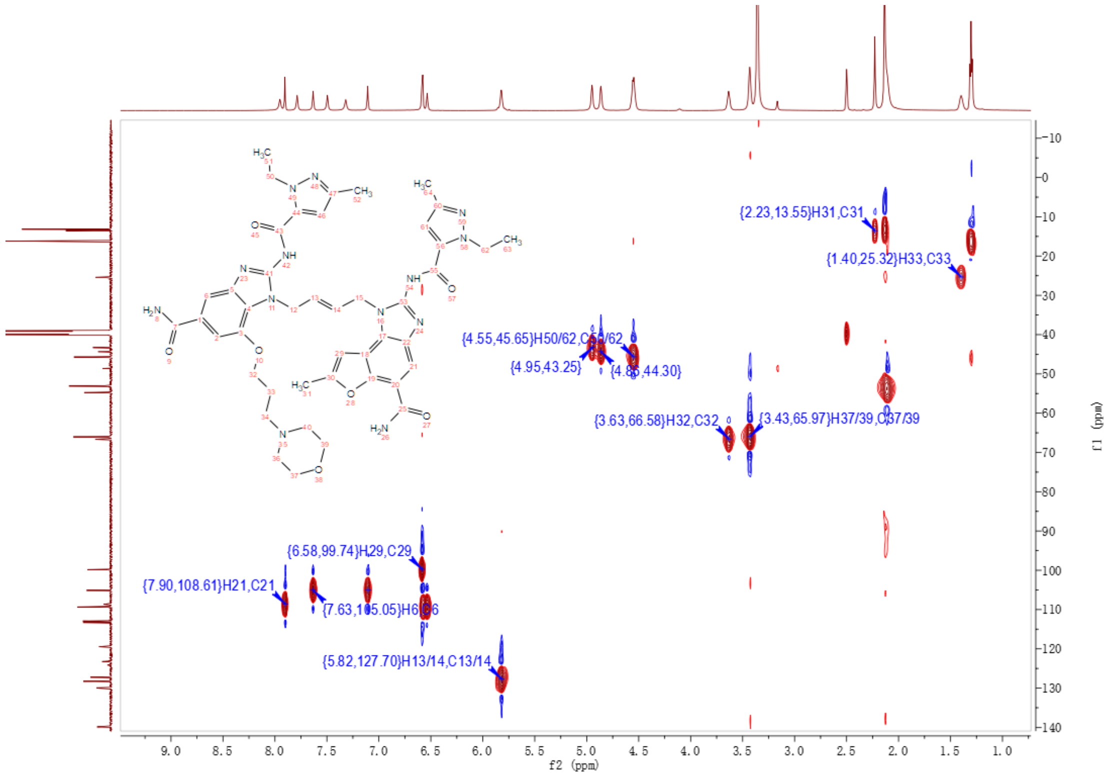

Compound HB3089 HMBC:

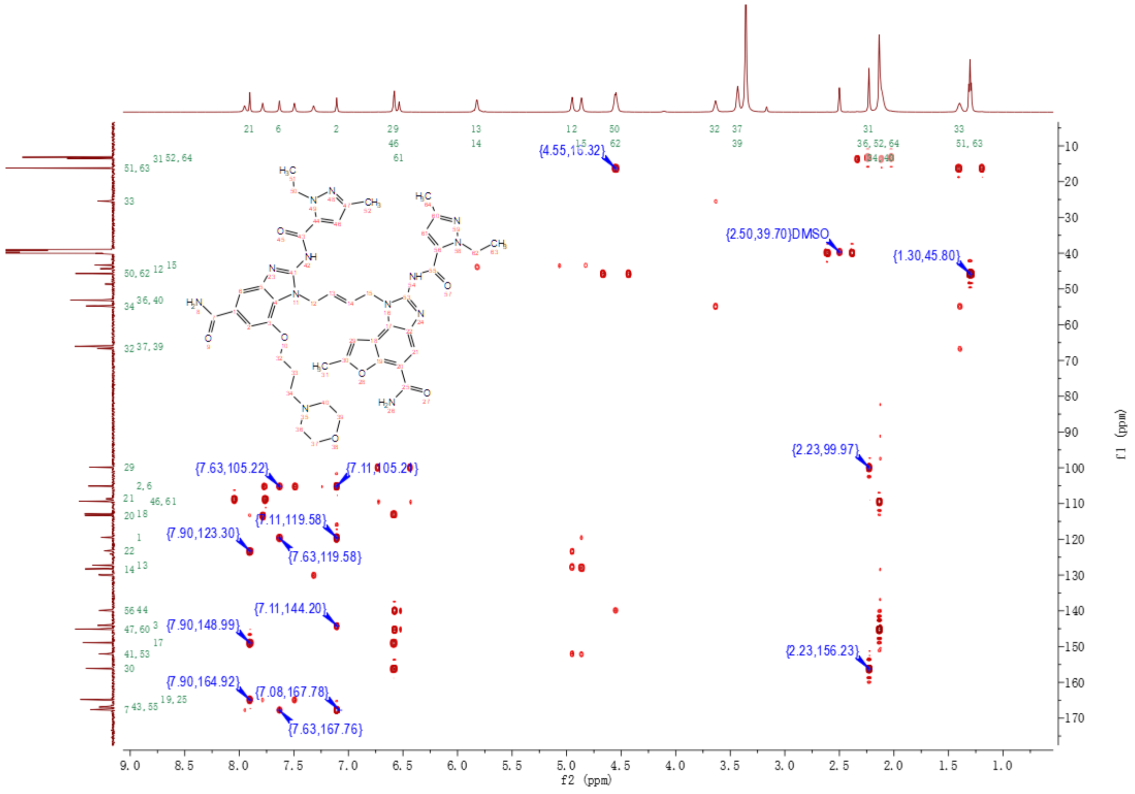

Compound HB3089 HRMS Data：

Qualitative Analysis Report

**Data Filename**	ESIH202104401.d	**Sample Name**	A4-ZXQ-3089
**Sample ID**		**Position**	P1-D4
**Instrument Name**	Agilent G6520 Q-TOF	**Acq Method**	20160322_MS_ESIH_POS_1min.m
**Acquired Time**	9/24/2021 18:15:42	**IRM Calibration Status**	Success
**DA Method**	small molecular data analysis method.m	**Comment**	ESIH by fangsu

User Spectra

Fragmentor Voltage	Collision Energy	Ionization Mode
175	0	ESI

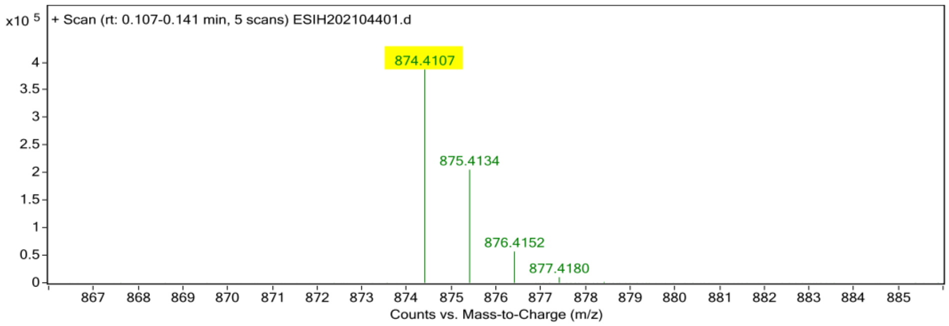

Formula Calculator Results

m/z	Calc m/z	Diff (mDa)	Diff (ppm)	Ion Formula	Ion
874.4107	874.4107	0.03	0.04	C44 H52 N13 O7	(M+H)+

--- End Of Report ---

1 Ramanjulu, J. M. et al. Design of amidobenzimidazole STING receptor agonists with systemic activity. Nature 564, 439-443, doi:10.1038/s41586-018-0705-y (2018).

Supplemental figure legends

Fig. S1 Chemical design and binding of compounds to STING variants. a Structural optimization of HB3089. **b** HB3089 and diABZI (compound 3) increased the thermal stability of various human STING (AQ, 293Q, H232) and mouse STING (R231) in differential scanning fluorimetry (DSF) assay. The data were shown as the mean value of d(RFU)/dt, n=3. **c** ΔTm values of HB3089 and diABZI binding to human STING variants and mouse STING.

Fig. S2 HB3089 activated the STING signaling. **a** HB3089 activated the phosphorylation of TBK1/IRF3 in dose-dependent manner. **b** HB3089 activated the phosphorylation of TBK1/IRF3 in time-dependent manner. **c, d** HB3089 dose-dependently increased the secretion of IP-10 and IFN-β in THP1-Dual cells after 24 h treatment. **e, f** HB3089 activated the ISG reporter in 293T-Dual-hSTING-R232 and 293T-Dual-hSTING-H232 cells after 24 h treatment.

Fig. S3 Anti-tumor effect of HB3089 in multiple allograft tumor models. a Body weight of 4T1 tumor-bearing Balb/c mice of Fig.1h. **b** Body weight of EMT6 tumor-bearing Balb/c mice of Fig. 1i. **c** HB3089 significantly inhibited the growth of B16F10 melanoma in C57BL/6 wild-type mice by intraperitoneal (IP) administration, n=10. **d** Body weight of B16F10 tumor-bearing C57BL/6 mice of Fig. S3c. **e** HB3089 could not inhibit the growth of B16F10 melanoma in C57BL/6 KO-STING mice by IP administration, n=6. **f** Body weight of B16F10 tumor-bearing C57BL/6 KO-STING mice of Fig. S3e. **g** HB3089 inhibited the growth of CT26 colon tumor in Balb/c mice by intravenous (IV) administration, n=6. **h** Body of CT26 tumor-bearing Balb/c mice

of Fig. S3g. **i** HB3089 dramatically inhibited the growth of LLC lung tumor in C57 mice by intratumoral (IT) administration, n=6. **j** Body of LLC tumor-bearing C57BL/6 mice of Fig. S3i. **k** HB3089 dramatically inhibited the growth of H22 liver tumor in C57 mice by either IV or IT administration, n=6. **l** Body of H22 tumor-bearing C57BL/6 mice of Fig. S3k. **m** HB3089 dramatically inhibited the growth of U14 cervical tumor in Kunming mice by either IV or IT administration, while diABZI did not significantly inhibit the growth of U14 cervical tumor by IV administration, n=6. **n** Body of U14 tumor-bearing Kunming mice of Fig. S3m. **o** HB3089 dramatically inhibited the growth of RENCA renal tumor in Balb/c mice by IT administration, n=6. **p** Body of RENCA tumor-bearing Balb/c mice of Fig. S3o. **q** LLC tumor growth in naïve mice and rechallenged mice of Fig. S3i (complete tumor regression by HB3089 treatment and 2 months tumor-free), the number of LLC tumor-bearing mice and total mice were shown, respectively. Arrow indicates the day of administration. $^{***}P<0.001$, $^{****}P<0.0001$, Two-way ANOVA.

Fig. S4 Effect of HB3089 on the immune subsets of blood and tumor tissues in 4T1 tumor-bearing Balb/c mice. a-c Frequency of monocytes, neutrophils and CD8T cells in blood (24 h) and tumor tissues (24 h and 72 h) of 4T1 tumor-bearing Balb/c mice. HB3089 was administrated by IV at 2 mg/kg. ns, not significant; $^{*}P<0.05$, $^{****}P<0.0001$, two-tailed t-test; n=8.

Fig. S5 Sample preparation and image processing of HB3089-bound STING. a A representative cryo-EM micrograph. **b** Flow chart of the image processing. **c** Representative 2D classes. **d** Gold-standard Fourier shell correlation (FSC) curves of

the final 3D reconstruction.

Fig. S6 Local resolution and representative density maps of our reconstruction of HB3089-bound STING. a Local resolution distribution of the reconstruction estimated by ResMap. **b** Density maps (mesh) and models of α-helices and β-sheets (ribbon). **c** The C2-reconstruction density maps of HB3089 with the high contour level. **d** The C2-reconstruction density maps (mesh) of HB3089 with fitting models. **e** The C2-reconstruction density maps of connector in HB3089-bound STING with the high contour level. **f** The density map (mesh) of the connector in HB3089-bound STING with fitting models.

Fig. S7 The binding mode of HB3089 in full-length human STING. a, b A side (**a**) and a top (**b**) views showing that HB-3089 rests into the binding pocket of LBD. Two monomers of human STING are colored in red and purple, respectively. The atomic model of HB3089 fits into its yellow-colored density map. **c** The map of non-symmetric agonist HB3089 displaying a two-fold symmetry appearance due to the imposed C2 average. **d** The binding surface of LBD to the agonist HB3089 is mainly hydrophobic. The binding pocket is showed in the surface mode which is colored according to its lipophilicity potential. The colors on the surface rang from dark cyan (most hydrophilic) to white to dark goldenrod (most lipophilic). **e** The detail interactions between the agonist HB3089 and its binding pocket in human STING. **f** The effects of STING mutations in binding pocket on HB3089-induced expression of the Interferon-stimulated gene (ISG) reporter. ISG fold change was calculated relative to Null vehicle. Data are presented as the mean ± SD of triplicate experiments.

Fig. S8 Structural comparisons of ligand-binding domains (LBDs). **a**, **b** Alignment of LBDs from previous structures. Full-length apo human STING (PDB 6NT5), apo isolated LBD (PDB 4EF5) and diABZI-bound isolated LBD (PDB 6DXL) are colored in grey, yellow and blue, respectively. One monomer of LBD is aligned and appears blurred for clarify. The compared models showed with a side (**a**) and a top (**b**) view. **c, d** The inter-Cα distance between residues His185 at LBDα1 of isolated LBD in the apo state (**c**) or diABZI-bound state (**d**). **e** The superposition of HB3809-bound LBD and compound 2-bound isolated LBD. **f** Close view of the red box region in **e**. **g** Close view of the blue box region in **e**.

Fig. S9 Sample preparation and image processing of SAVI-related mutant V147L. **a** A representative cryo-EM micrograph. **b** Flow chart of image processing. **c** Representative 2D classes. **d** Gold-standard Fourier shell correlation (FSC) curves of the final 3D reconstruction.

Fig. S10 Local resolution and representative density maps of our reconstruction of SAVI-related mutant V147L. **a** Local resolution distribution of the reconstruction estimated by ResMap. **b** Density maps (mesh) and models of α-helices and β-sheets (ribbon). **c** The C2-reconstruction density maps of connector in mutant V147L with the high contour level. **d** The density map (mesh) of the connector in in mutant V147L with fitting models.

Fig. S11 The outer transmembrane helices of STING are vibrational. **a** The C1 structure determination of the HB3089-bound STING. **b** The C1 structure determination of the mutant V147L. **c** Gold-standard Fourier shell correlation (FSC)

curves of the final C1 3D reconstruction of STING with HB3089 and the mutation V147L, respectively. **d** The side views showing the C1 density map of HB3089-bound STING. Two protomers of STING are colored in purple and red, respectively. It shows that the outer transmembrane helices (TM1s and TM3s) are vibrational asymmetrically, as compared with the inner ones. **d** The asymmetric density of HB3089 clearly showing the C1 structure of HB3089. **f** Local B-factor analysis of the C1 STING with HB3089 showing that the outer transmembrane helices are vibrational. **g** The side view showing the C1 density map of STING with the mutation V147L. Two protomers of STING are colored in green and blue, respectively. It shows that its outer transmembrane helices (TM1s and TM3s) are vibrational asymmetrically, as compared with the inner ones. **h** Local B-factor analysis of the C1 STING with the mutation V147L showing that its outer transmembrane helices are vibrational.

Fig. S1

a

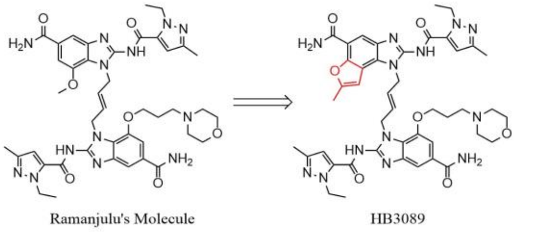

b

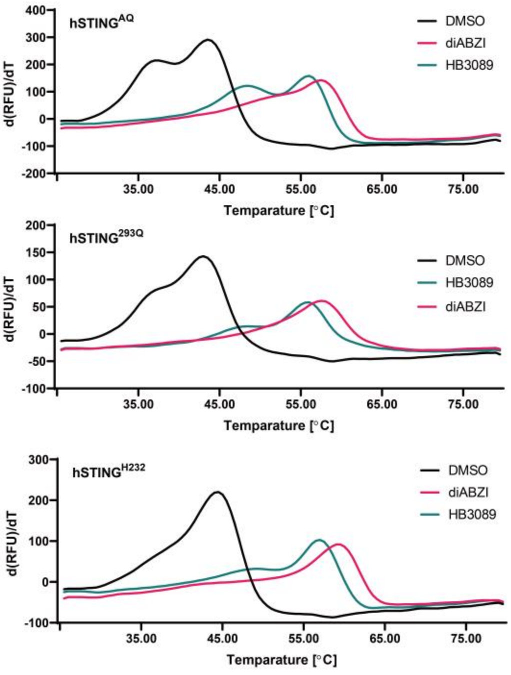

c

Ligand	ΔTm (℃) hSTING				ΔTm (℃) mSTING
	R232	AQ	293Q	H232	R231
diABZI	16	14	14.5	15	16.5
HB3089	11.5	12.5	13	12.5	15

Fig. S2

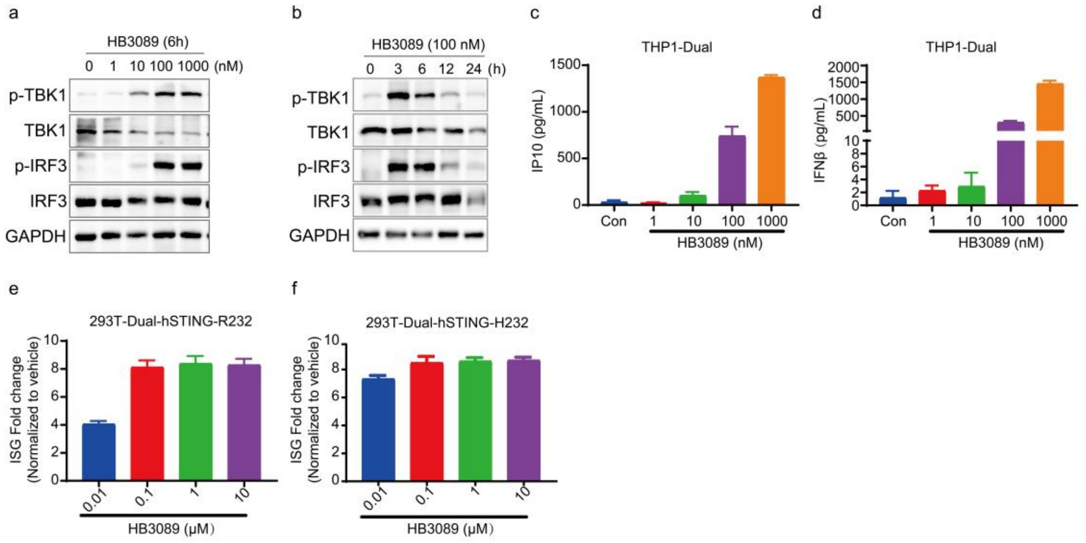

a
HB3089 (6h)
0 1 10 100 1000 (nM)
p-TBK1
TBK1
p-IRF3
IRF3
GAPDH
b
HB3089 (100 nM)
0 3 6 12 24 (h)
p-TBK1
TBK1
p-IRF3
IRF3
GAPDH
c
THP1-Dual
IP10 (pg/mL)
Con 1 10 100 1000
HB3089 (nM)
d
THP1-Dual
IFNβ (pg/mL)
Con 1 10 100 1000
HB3089 (nM)
e
293T-Dual-hSTING-R232
ISG Fold change (Normalized to vehicle)
0.01 0.1 1 10
HB3089 (μM)
f
293T-Dual-hSTING-H232
ISG Fold change (Normalized to vehicle)
0.01 0.1 1 10
HB3089 (μM)

Fig. S3

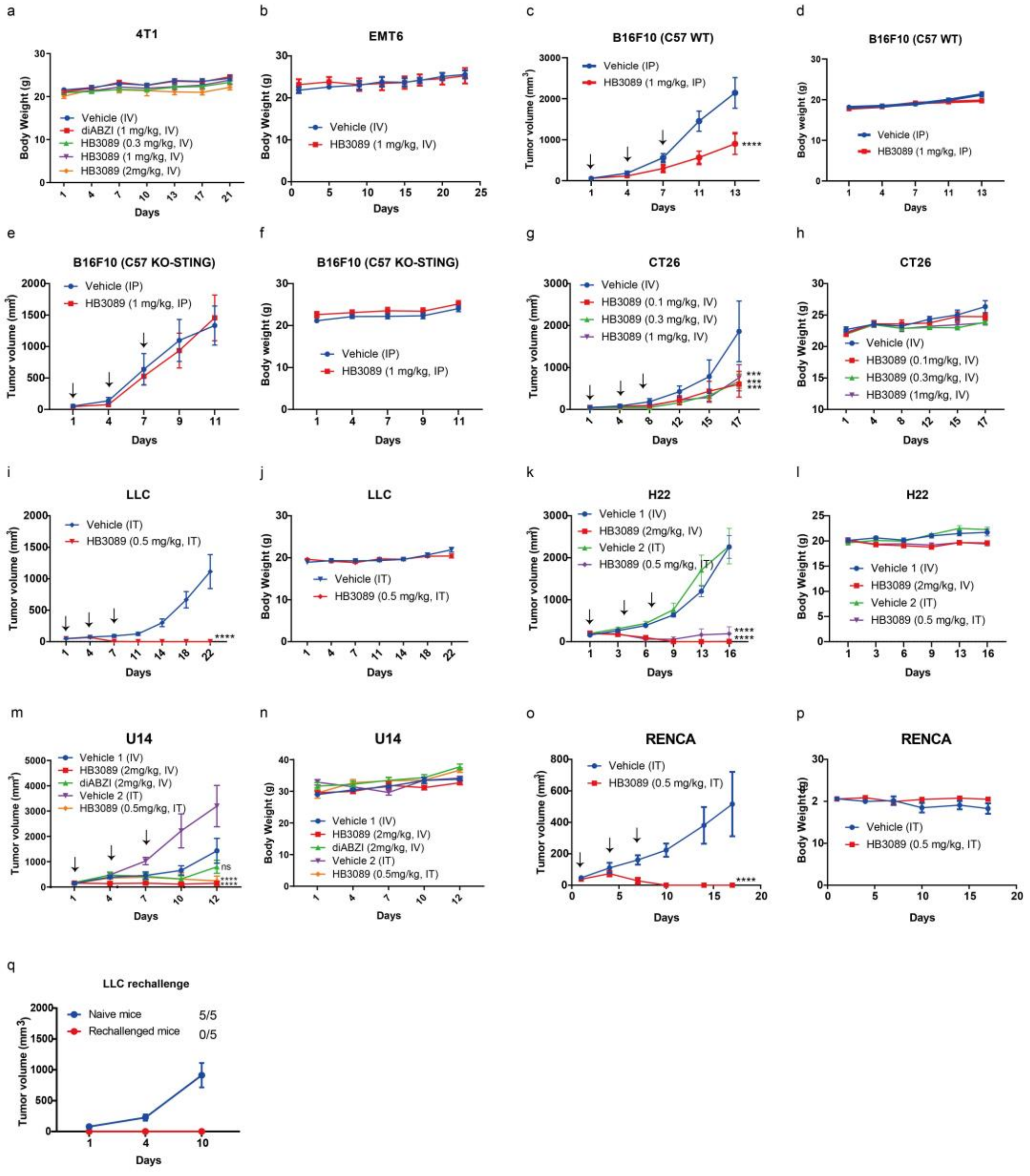
a
4T1
Body Weight (g)
Vehicle (IV)
diABZI (1 mg/kg, IV)
HB3089 (0.3 mg/kg, IV)
HB3089 (1 mg/kg, IV)
HB3089 (2mg/kg, IV)
Days
b
EMT6
Body Weight (g)
Vehicle (IV)
HB3089 (1 mg/kg, IV)
Days
c
B16F10 (C57 WT)
Tumor volume (mm3)
Vehicle (IP)
HB3089 (1 mg/kg, IP)
****
Days
d
B16F10 (C57 WT)
Body weight (g)
Vehicle (IP)
HB3089 (1 mg/kg, IP)
Days
e
B16F10 (C57 KO-STING)
Tumor volume (mm3)
Vehicle (IP)
HB3089 (1 mg/kg, IP)
Days
f
B16F10 (C57 KO-STING)
Body weight (g)
Vehicle (IP)
HB3089 (1 mg/kg, IP)
Days
g
CT26
Tumor volume (mm3)
Vehicle (IV)
HB3089 (0.1 mg/kg, IV)
HB3089 (0.3 mg/kg, IV)
HB3089 (1 mg/kg, IV)
***
Days
h
CT26
Body Weight (g)
Vehicle (IV)
HB3089 (0.1mg/kg, IV)
HB3089 (0.3mg/kg, IV)
HB3089 (1mg/kg, IV)
Days
i
LLC
Tumor volume (mm3)
Vehicle (IT)
HB3089 (0.5 mg/kg, IT)
****
Days
j
LLC
Body Weight (g)
Vehicle (IT)
HB3089 (0.5 mg/kg, IT)
Days
k
H22
Tumor volume (mm3)
Vehicle 1 (IV)
HB3089 (2mg/kg, IV)
Vehicle 2 (IT)
HB3089 (0.5 mg/kg, IT)
****
Days
l
H22
Body Weight (g)
Vehicle 1 (IV)
HB3089 (2mg/kg, IV)
Vehicle 2 (IT)
HB3089 (0.5 mg/kg, IT)
Days
m
U14
Tumor volume (mm3)
Vehicle 1 (IV)
HB3089 (2mg/kg, IV)
diABZI (2mg/kg, IV)
Vehicle 2 (IT)
HB3089 (0.5mg/kg, IT)
ns
****
Days
n
U14
Body Weight (g)
Vehicle 1 (IV)
HB3089 (2mg/kg, IV)
diABZI (2mg/kg, IV)
Vehicle 2 (IT)
HB3089 (0.5mg/kg, IT)
Days
o
RENCA
Tumor volume (mm3)
Vehicle (IT)
HB3089 (0.5 mg/kg, IT)
****
Days
p
RENCA
Body Weight (g)
Vehicle (IT)
HB3089 (0.5 mg/kg, IT)
Days
q
LLC rechallenge
Tumor volume (mm3)
Naive mice 5/5
Rechallenged mice 0/5
Days

Fig. S4

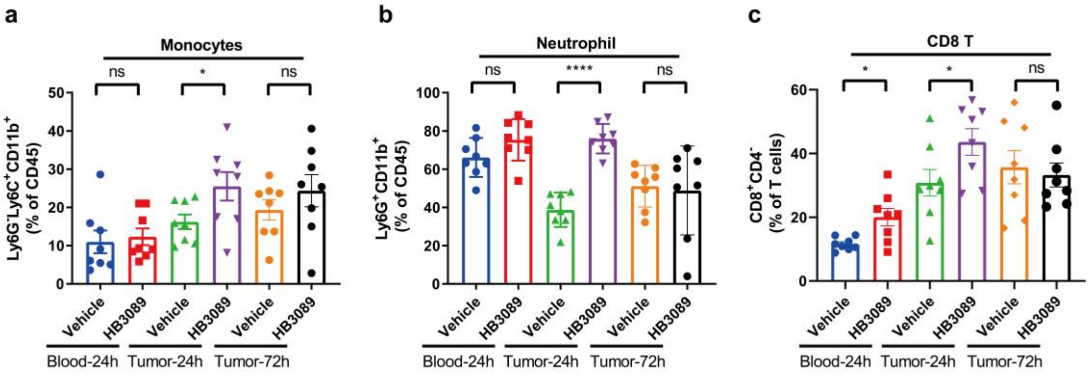
a
Monocytes
ns
*
ns
Ly6G⁻Ly6C⁺CD11b⁺ (% of CD45)
Vehicle
HB3089
Blood-24h
Tumor-24h
Tumor-72h
b
Neutrophil
ns
****
ns
Ly6G⁺CD11b⁺ (% of CD45)
c
CD8 T
*
*
ns
CD8⁺CD4⁻ (% of T cells)

Fig. S5

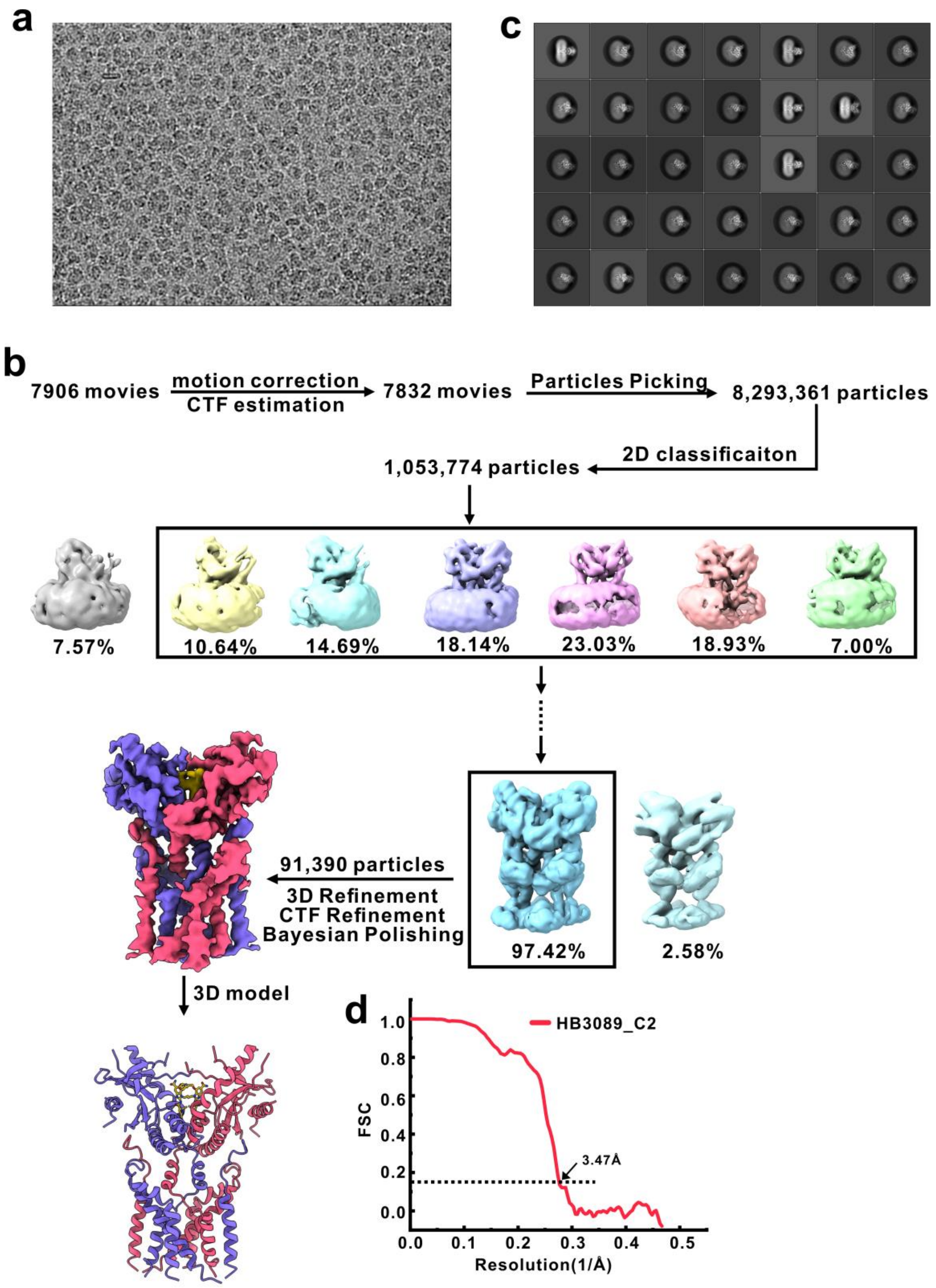
a
c
b
7906 movies
motion correction
CTF estimation
7832 movies
Particles Picking
8,293,361 particles
2D classificaiton
1,053,774 particles
7.57%
10.64%
14.69%
18.14%
23.03%
18.93%
7.00%
91,390 particles
3D Refinement
CTF Refinement
Bayesian Polishing
97.42%
2.58%
3D model
d
HB3089_C2
FSC
1.0
0.8
0.6
0.4
0.2
0.0
3.47Å
0.0
0.1
0.2
0.3
0.4
0.5
Resolution(1/Å)

Fig. S6

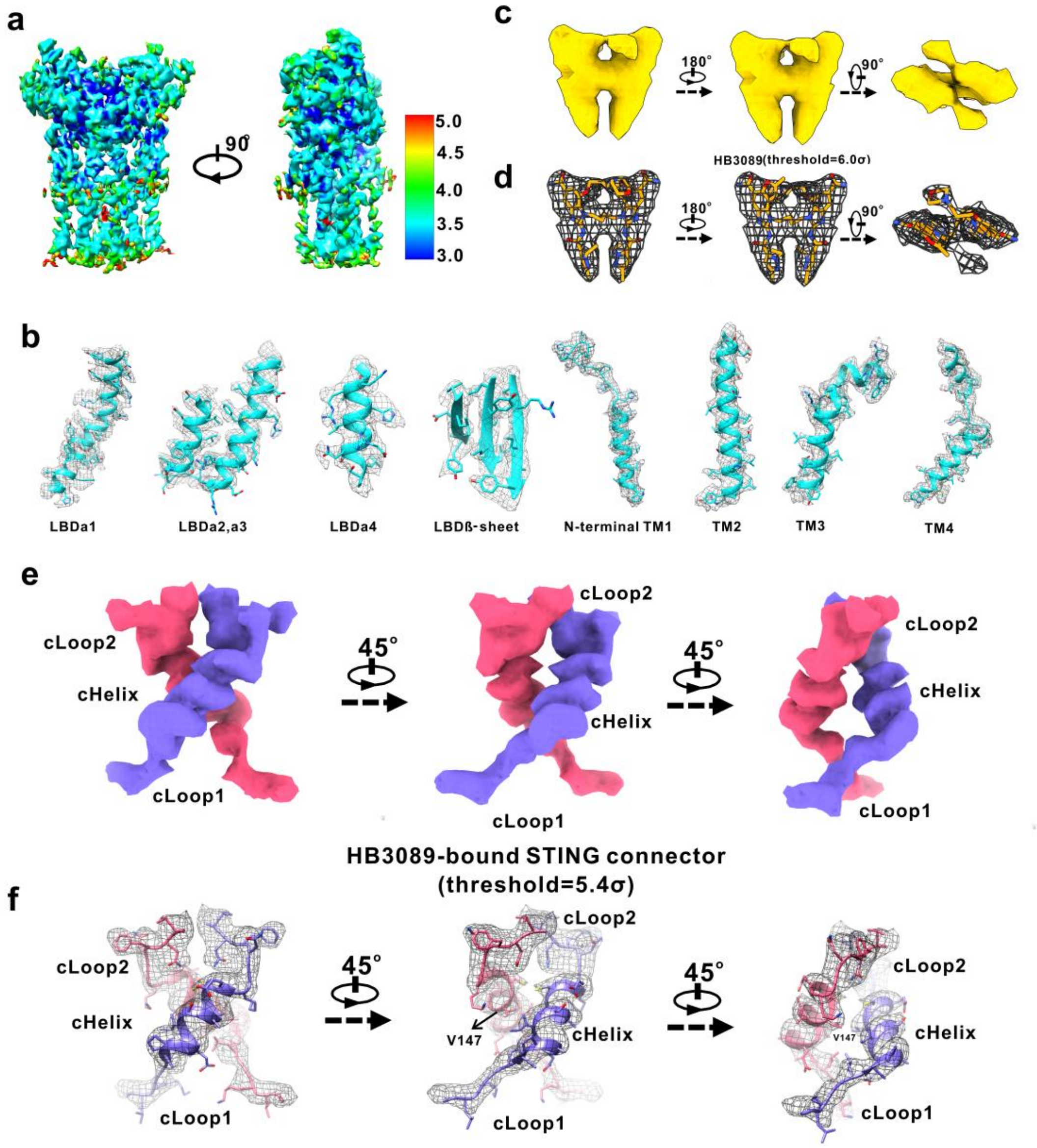

a
90°
5.0
4.5
4.0
3.5
3.0
c
180°
90°
HB3089(threshold=6.0σ)
d
180°
90°
b
LBDa1
LBDa2,a3
LBDa4
LBDß-sheet
N-terminal TM1
TM2
TM3
TM4
e
cLoop2
cHelix
cLoop1
45°
cLoop2
cHelix
cLoop1
45°
cLoop2
cHelix
cLoop1
HB3089-bound STING connector
(threshold=5.4σ)
f
cLoop2
cHelix
cLoop1
45°
cLoop2
V147
cHelix
cLoop1
45°
cLoop2
V147
cHelix
cLoop1

Fig. S7

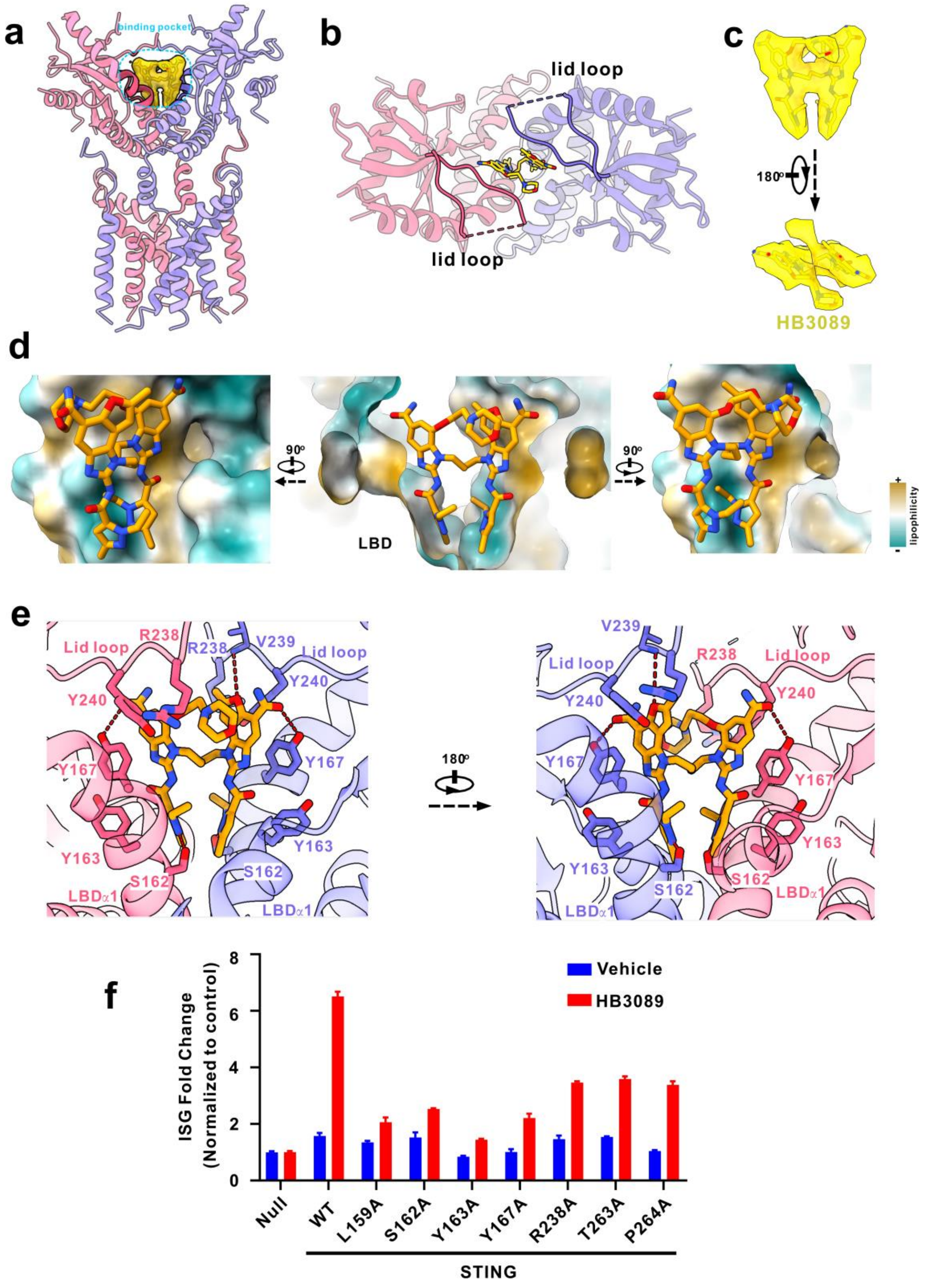

a
binding pocket
b
lid loop
lid loop
c
180°
HB3089
d
90°
90°
LBD
+
lipophilicity
-
e
Lid loop
R238
R238
V239
Lid loop
Y240
Y240
Y167
Y167
Y163
Y163
S162
S162
LBDα1
LBDα1
180°
f
Vehicle
HB3089
ISG Fold Change
(Normalized to control)
8
6
4
2
0
Null
WT
L159A
S162A
Y163A
Y167A
R238A
T263A
P264A
STING

Fig. S8

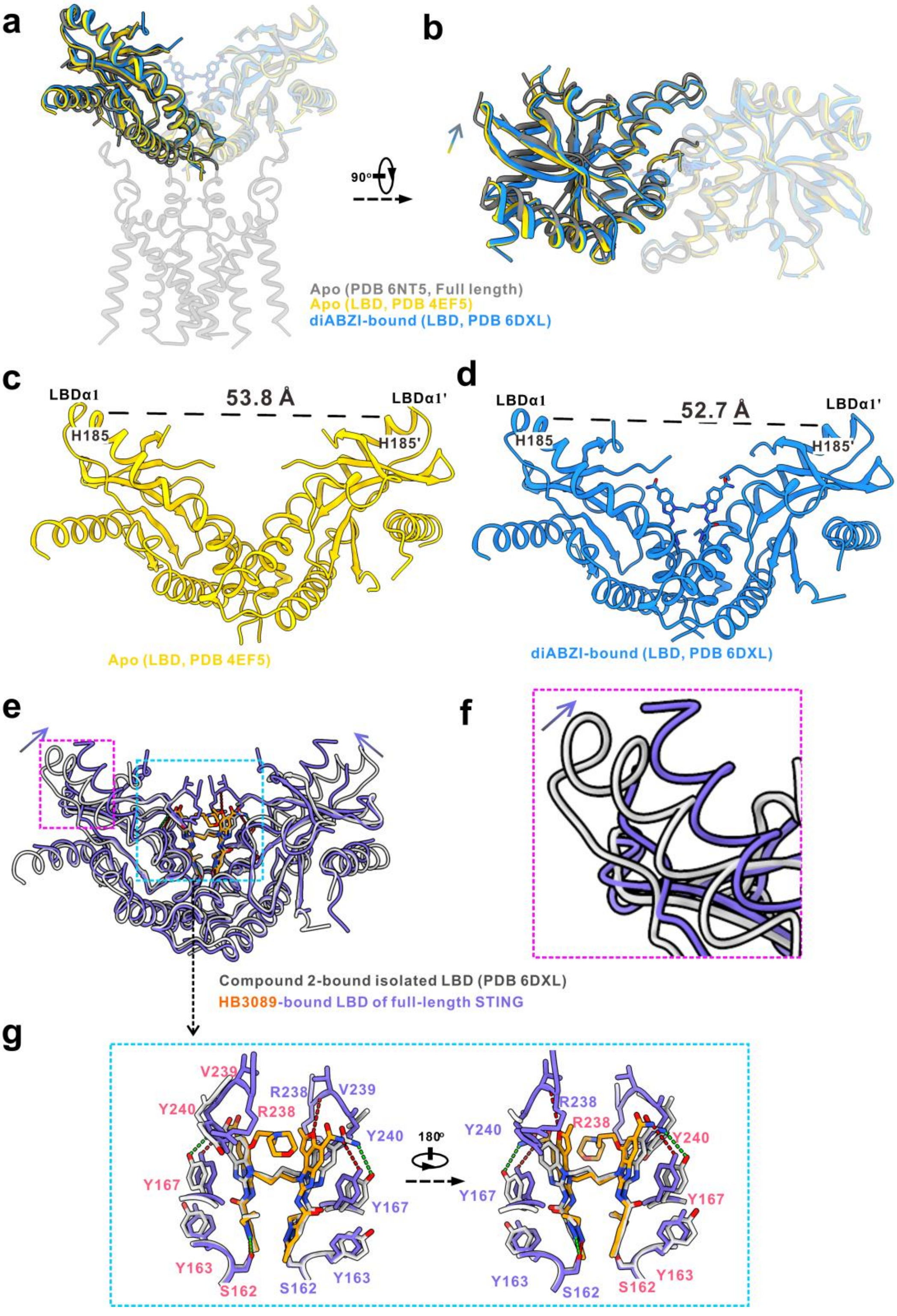
a
b
90°
Apo (PDB 6NT5, Full length)
Apo (LBD, PDB 4EF5)
diABZI-bound (LBD, PDB 6DXL)
c
LBDα1
53.8 Å
LBDα1'
H185
H185'
Apo (LBD, PDB 4EF5)
d
LBDα1
52.7 Å
LBDα1'
H185
H185'
diABZI-bound (LBD, PDB 6DXL)
e
f
Compound 2-bound isolated LBD (PDB 6DXL)
HB3089-bound LBD of full-length STING
g
V239
R238
V239
Y240
R238
Y240
Y167
Y167
Y163
S162
S162
Y163
180°
R238
Y240
R238
Y240
Y167
Y167
Y163
S162
S162
Y163

Fig. S9

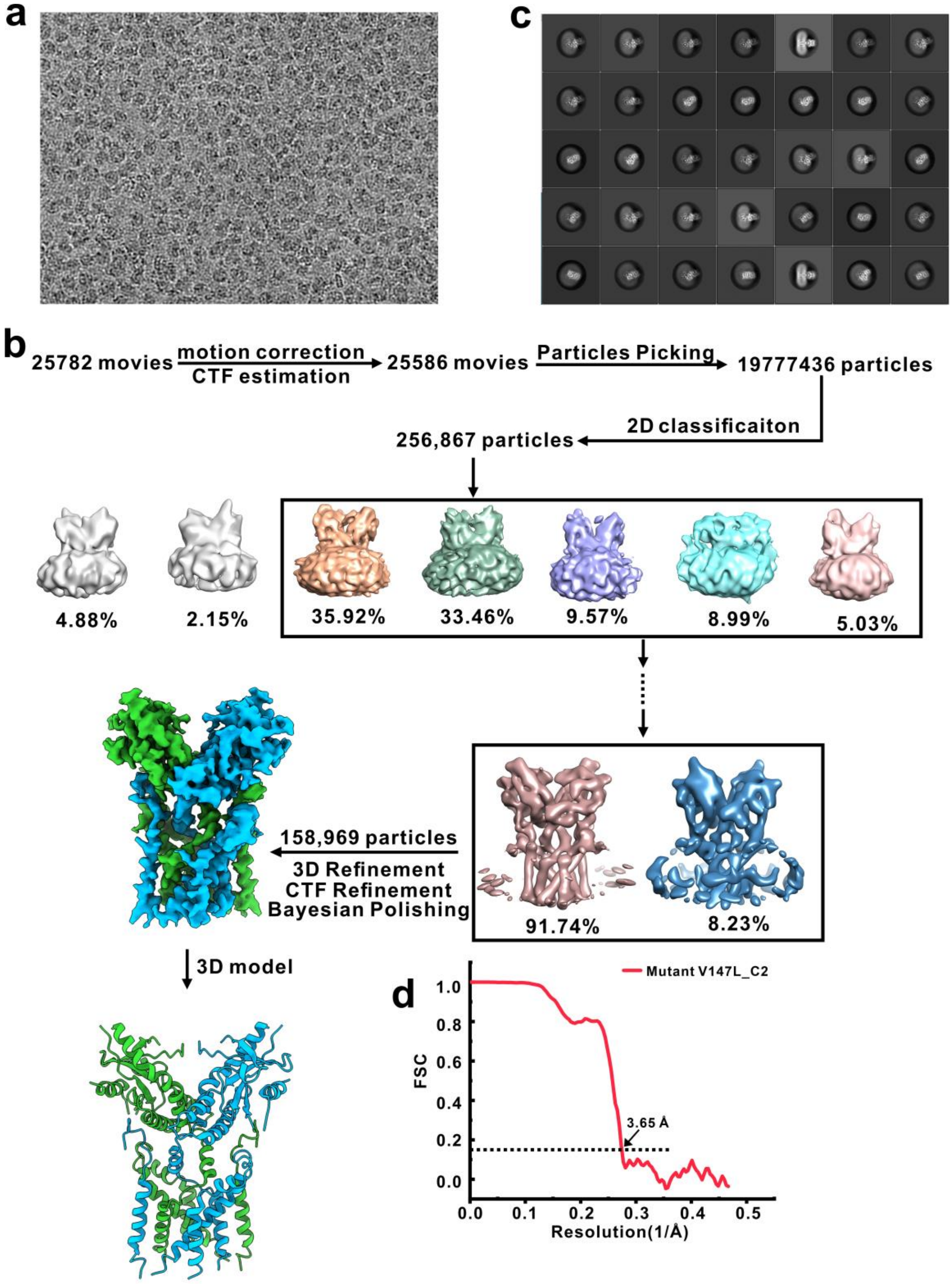
a
c
b
25782 movies
motion correction
CTF estimation
25586 movies
Particles Picking
19777436 particles
2D classificaiton
256,867 particles
4.88%
2.15%
35.92%
33.46%
9.57%
8.99%
5.03%
158,969 particles
3D Refinement
CTF Refinement
Bayesian Polishing
91.74%
8.23%
3D model
d
Mutant V147L_C2
FSC
3.65 Å
Resolution(1/Å)
1.0
0.8
0.6
0.4
0.2
0.0
0.0
0.1
0.2
0.3
0.4
0.5

Fig. S10

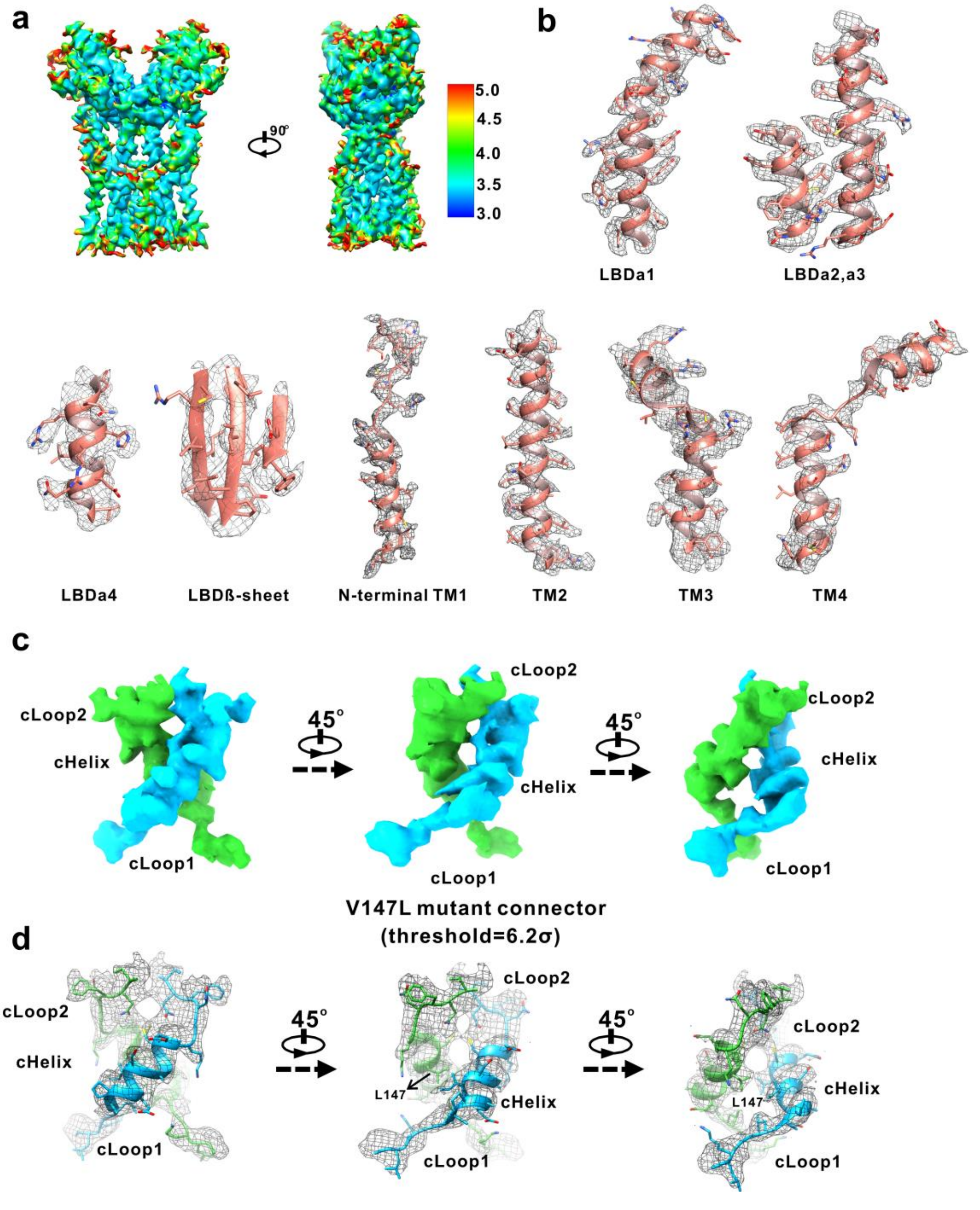
a
90°
5.0
4.5
4.0
3.5
3.0
b
LBDa1
LBDa2,a3
LBDa4
LBDß-sheet
N-terminal TM1
TM2
TM3
TM4
c
cLoop2
cHelix
cLoop1
45°
V147L mutant connector
(threshold=6.2σ)
d
L147

Fig. S11

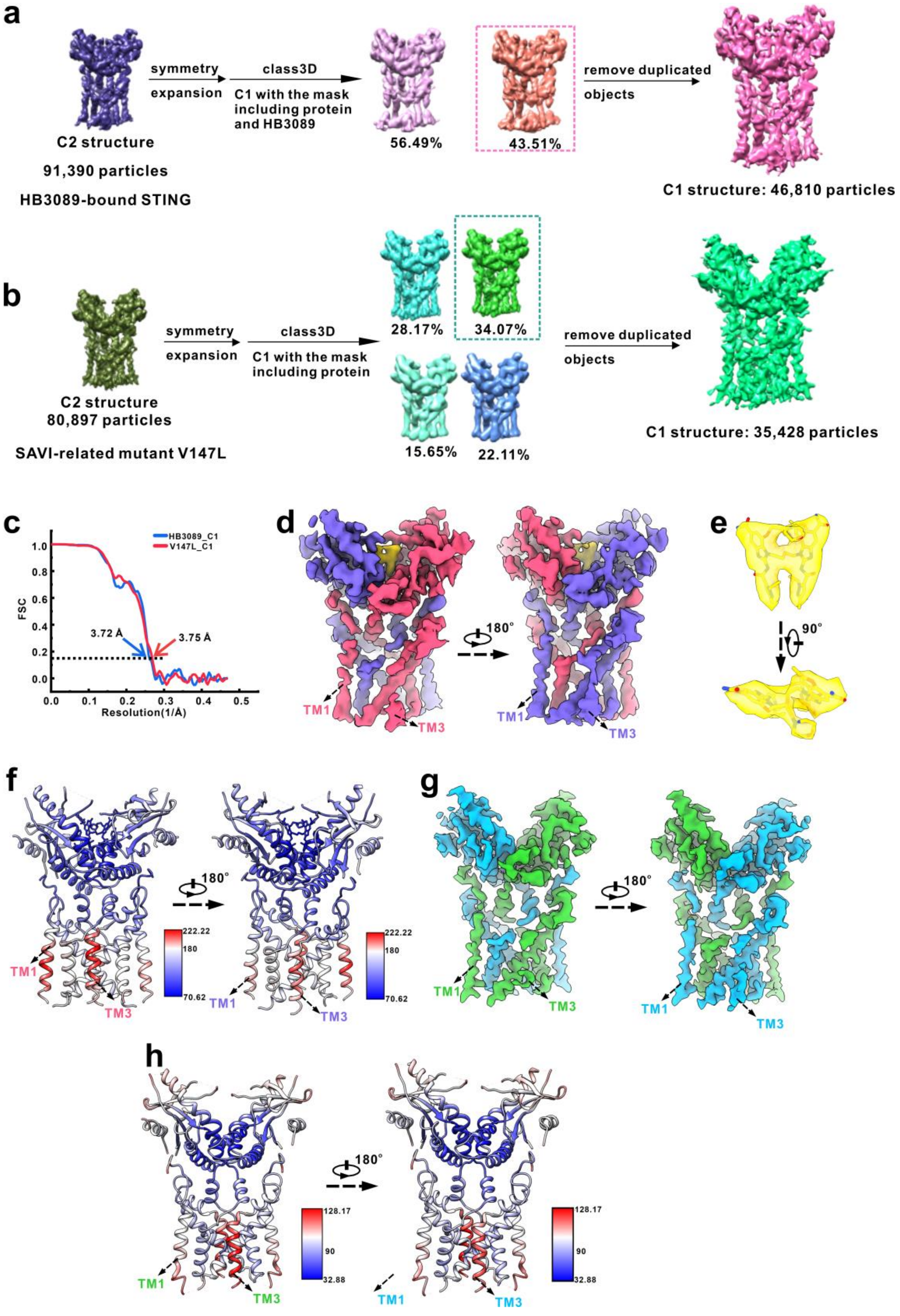
a
symmetry expansion
class3D
C1 with the mask including protein and HB3089
remove duplicated objects
C2 structure
91,390 particles
HB3089-bound STING
56.49%
43.51%
C1 structure: 46,810 particles
b
symmetry expansion
class3D
C1 with the mask including protein
28.17%
34.07%
15.65%
22.11%
remove duplicated objects
C2 structure
80,897 particles
SAVI-related mutant V147L
C1 structure: 35,428 particles
c
HB3089_C1
V147L_C1
FSC
Resolution(1/Å)
3.72 Å
3.75 Å
d
180°
TM1
TM3
e
90°
f
222.22
180
70.62
g
h
128.17
90
32.88

Table S1. Pharmacokinetics of HB3089 and diABZI in mice*.

Compound	Dose (mg/kg)	C_{max} (μg/mL)	T_{max} (h)	$T_{1/2}$ (h)	$AUC_{0\rightarrow\infty}$ (μg·h/mL)	CL (mL/min/kg)	V_{ss} (L/kg)	F (%)
**HB3089**	**2 (SC)**	**1.79±0.23**	**0.58±0.29**	**1.98±0.13**	**4.60±0.84**	**-**	**-**	**155**
	**2 (IV)**	**-**	**-**	**3.18±0.49**	**3.12±0.59**	**11.0±2.3**	**1.86±0.18**	
**diABZI (Compound3)**	**2 (SC)**	**1.06±0.08**	**0.42±0.29**	**0.87±0.28**	**1.36±0.28**	**-**	**-**	**76.4**
	**2 (IV)**	**-**	**-**	**1.36±0.17**	**1.76±0.57**	**20.3±6.0**	**0.94±0.46**	

*n=3.

Table S2. Cryo-EM data collection, refinement and validation statistics.

	STING-HB3089	STING mutant V147L
**Data collection and processing**		
Magnification	81,000	81,000
Voltage(kV)	300	300
Electron exposure(e^-/Å^2)	70	60
Defocus range(μm)	-2.0	-1.5
Pixel size(Å)	1.071	1.071
Symmetry imposed	C2	C2
Initial particle images(no.)	8,293,361	7,297,959
Final particle images(no.)	91,390	158,969
Map resolution(Å)	3.45	3.65
FSC threshold	0.143	0.143
**Refinement**		
Model resolution(Å)	3.6	3.8
FSC threshold	0.5	0.5
Map sharpening B factor(Å^2)	-21	-30
Model Composition		
Non-hydrogen atoms	4922	4870
Protein residues	618	620
Ligands	1	-
B factors(Å^2)		
Protein	118.88	72.01
Ligand	64.80	-
R.m.s.deviations		
Bond length(Å^2)	0.007	0.006
Bond angle(°)	1.082	0.851
Validation		
Molprobity score	1.52	1.74
Clashscore	4.09	12.54
Ramachandran plot		
Favored(%)	95.29	97.32
Allowed(%)	4.71	2.68
Outliers(%)	0.00	0.00
